# Scaffold-Hopping Design and Synthesis of Thieno[3,2-*d*]pyrimidines: Anticancer Activity, Apoptosis Induction, and In Silico Inhibition of CDKs

**DOI:** 10.3390/ijms26178528

**Published:** 2025-09-02

**Authors:** Zukela Ruzi, Anvarjon Buronov, Lifei Nie, Azizbek Nasrullaev, Zarifa Murtazaeva, Rustamkhon Kuryazov, Jiangyu Zhao, Thomas Efferth, Haji Akber Aisa, Khurshed Bozorov

**Affiliations:** 1 State Key Laboratory Basis of Xinjiang Indigenous Medicinal Plants Resource Utilization, Xinjiang Technical Institute of Physics and Chemistry, Chinese Academy of Sciences, South Beijing Rd 40-1, Urumqi 830011, China; 2 School of Chemistry and Chemical Engineering, Changji University, Changji 831100, China; 3 Department of Organic Synthesis and Bioorganic Chemistry, Institute of Biochemistry, Samarkand State University, University Blvd. 15, Samarkand 140104, Uzbekistan; 4 Department of Chemistry, Urgench State University, Kh. Olimjon st. 14, Urgench 220100, Uzbekistan; 5 Department of Pharmaceutical Biology, Institute of Pharmaceutical and Biomedical Sciences, Johannes Gutenberg University, Staudinger Weg 5, 55128 Mainz, Germany

**Keywords:** anticancer activity, apoptosis induction, cell cycle arrest, cyclin-dependent kinases (CDKs), CDK1, in silico study, molecular docking, scaffold-hopping synthesis, thieno[3,2-*d*]pyrimidines

## Abstract

Two series of tricyclic thieno[3,2-*d*]pyrimidines were synthesized, achieving yields of up to 97%. The tricyclic thieno[3,2-*d*]pyrimidines examined in this study are synthetic analogs of the deoxyvasicinone alkaloids, where the thiophene ring substitutes for the benzene ring. A systematic investigation was conducted on the scaffold-hopping strategy of these alkaloids, emphasizing the selective synthesis and anticancer properties of thieno[3,2-*d*]pyrimidines. The anticancer evaluation was performed on human cancer cell lines, specifically cervical HeLa and colon HT-29 carcinoma cells. Additional bioassays included cell migration analyses, cell cycle progression, apoptosis, and molecular docking analyses. Furthermore, molecular docking studies showed that the most active small molecule **6e** is likely to disrupt the cell cycle process through targeting CDKs (Cyclin-dependent kinases), leading to the inhibition of tumor cell proliferation.

## 1. Introduction

Cancer is indeed the second leading cause of death worldwide [1,2,3,4,5]. More advancements are needed in medicinal chemistry to address this issue, where synthetic and natural compounds play a crucial role [6]. Several small-molecule-based antitumor agents for cancer treatment and diagnosis have been developed thus far [7,8,9,10]. However, these methods had some limitations, as did the candidate targets identified [9]. Therefore, the preferential synthesis and anticancer activity of pharmacophore-containing compounds represent an important area of focus. Since the scaffold-hopping approach was introduced by Schneider and colleagues in 1999 [11], it has been effectively utilized in related sciences [12,13]. Scaffold hopping is a method for discovering or synthesizing structurally novel compounds by modifying the central or major core structure of known active lead compounds [14,15]. This strategy encompasses several key methods fundamental to anticancer drug design, which include bioisosteric replacement [16,17], high-throughput screening [18], fragment-based drug discovery [19], structure–activity relationships [20], and others.

**Figure 1:**
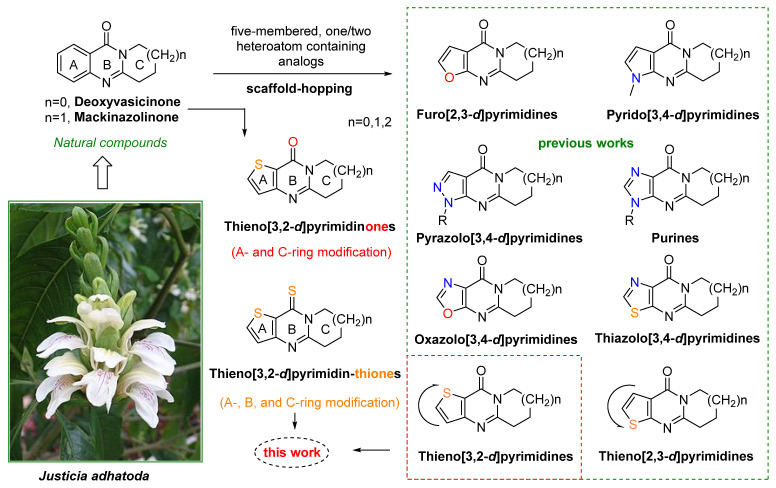
The structural scaffold-hopping approach applied in this study [17,21,23,25,26,31,34,35,36].

Recently, we conducted a scientific investigation into scaffold-hopping research [21,22,23,24,25,26] of the synthetic analogs of deoxyvasicinone [27] and mackinazolinone [28], which were natural alkaloids isolated from the medicinal plants *Justicia adhatoda* [29], *Adhatoda vasica* [30], and *Mackilaya subulata* Philipson [31], respectively (Figure 1). Most of these pyrimidine-based small molecules exhibited potential antitumor activity. In these studies, the benzene ring of deoxyvasicinone and mackinazolinone was replaced with five-membered heterocycles [24], yielding a novel class of bi/tricyclic condensed pyrimidines [32,33], including thieno[2,3-*d*]pyrimidine [23], pyrrolo[2,3-*d*]pyrimidine [34], furo[2,3-*d*]pyrimidine, pyrazolo[3,4-*d*]pyrimidine [35,36], oxazolo[5,4-*d*]pyrimidine [17], thiazolo[5,4-*d*]pyrimidine [26,37], and purine [21] scaffolds. Here, we continued our systematic research on the scaffold-hopping strategy of the alkaloids described above and studied the preferential synthesis and anticancer activity of thieno[3,2-*d*]pyrimidines, which feature a thiophene ring oriented in a reverse position compared to the earlier synthesized thieno[2,3-*d*]pyrimidine system. Thus, the tricyclic thieno[3,2-*d*]pyrimidinones that are the focus of this study are synthetic analogs of the deoxyvasicinone alkaloids, with the thiophene substituting the benzene ring (A-ring) (Figure 1).

**Figure 2:**
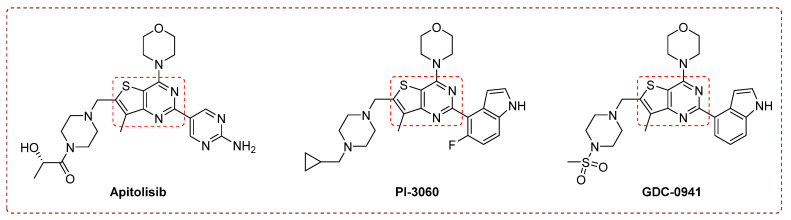
The structural scaffold-hopping approach applied in this study (continuation).

The introduction of the thiophene fragment is a common feature in most annulated thiophene-related pyrimidines successfully utilized in medicinal chemistry. Likewise, thieno[3,2-*d*]pyrimidines represent an important class of chemical compounds with significant therapeutic potential for human treatment [38,39]. For example, apitolisib (Figure 2), a bicyclic thienopyrimidine-based drug, is in clinical phases for the treatment of solid cancers [40,41,42,43], and two other modified analogs of apitolisib (PI-3060 and GDC-0941, Figure 2) are currently undergoing clinical trials [44,45].

Recent studies highlight that CDK7 and CDK9, unlike classical CDKs, regulate transcription and support cancer cell survival independently of cyclin binding [46,47,48,49]. In several cancer types, these kinases demonstrate altered expression patterns or enzymatic dysregulation, which can affect the transcription of survival-related genes and contribute to malignancy [50,51]. For this reason, they have attracted increasing interest as potential molecular targets in the development of small-molecule inhibitors aimed at modulating gene expression and inducing apoptosis in tumor cells. In this context, we designed and synthesized a series of tricyclic thieno[3,2-*d*]pyrimidines and investigated their potential binding interactions with selected CDKs using molecular docking tools. We selected a rigid fused-ring system to ensure a favorable orientation in the ATP-binding pocket, with particular attention to hydrogen bonding and π–π stacking interactions that could support kinase inhibition [52,53,54]. Given the structural resemblance of our scaffold to known CDK-binding chemotypes and its rigid planar framework, which favors optimal fit within transcriptional CDK active sites, we hypothesized that the obtained compounds could modulate CDK activity, thereby interfering with transcriptional regulation and inducing apoptosis in cancer cells.

Additionally, further investigations using this class of compounds may reveal other interesting findings, as follows:

1. The total synthesis of other tricyclic analogs of deoxyvasicinone could provide novel tricyclic annulated heterocyclic systems for further research of pharmacological interest in related fields.
2. Convenient synthetic methods for these heterocycles could be used to modify quinazolinone scaffolds via a scaffold-hopping strategy.

The one-pot synthesis of tricyclic thieno[3,2-*d*]pyrimidinones focused on the condensation reaction of cyclic lactams and thieno[3,2-*d*]pyrimidin-thiones with Lawesson’s reagent. Anticancer evaluations were performed using human cancer cell lines, including HeLa cervical and HT-29 colon carcinoma cells. Additional bioassays were performed using cell migration, cell cycle, apoptosis, and molecular docking analyses. It should be noted that earlier studies demonstrated that the parent alkaloids deoxyvasicinone and mackinazolinone exhibited only modest or weak anticancer activity. In contrast, our scaffold-hopped analogs, particularly those with thiophene rings, showed significantly more potent cytotoxicity and selectivity toward the HeLa and HT-29 cell lines. These results confirm that natural products are an effective foundation for designing superior synthetic analogs.

## 2. Results and Discussion

### 2.1. Synthesis

**Scheme 1:**
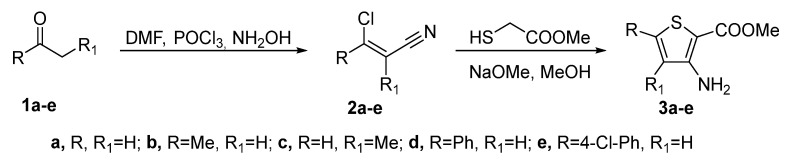
Synthesis of 3-amino-thiophene-2-carboxylate synthons **3a-e**.

The most common synthetic pathways for thieno[3,2-*d*]pyrimidinones (**5a-o**) involve cyclizing 3-amino-thiophene-2-carboxylate derivatives with a one-carbon source [55,56,57], such as formic acid, triethyl orthoformate, or a primary amine. In this study, 3-amino-thiophene-2-carboxylate synthons (**3a-e**) were synthesized in two steps (Scheme 1). First, substituted chloronitriles (**2a-e**) were produced from nucleophilic reagents (dimethylformamide (DMF) and phosphorus oxychloride (POCl_3_;)) and the appropriate aldehydes or ketones (**1a-e**). The reaction of methyl 2-mercaptoacetate with nitriles (**2a–e**) under basic conditions using NaOMe yielded the target 3-amino-thiophene-2-carboxylates in satisfactory yields (85–91%).

The synthesis of thieno[3,2-*d*]pyrimidinone began with the optimization of the reaction conditions [31,34]. This was done using **3a** and 2-pyrrolidone (**4a**), as well as phosphorus oxychloride (POCl_3_;) in dichloromethane (DCM), while stirring at reflux (see Table 1, entry 1). After five hours of refluxing, tricyclic thieno[3,2-*d*]pyrimidinone **5a** was obtained with a yield of 42%. Annulation of **3a** occurred swiftly, producing **5a** within a few minutes as confirmed by thin-layer chromatography (TLC) when the reaction commenced in dichloroethane (DCE) at 80 °C. After five hours, the reaction concluded with a 79% yield of **5a** (see Table 1, entry 2).

**Scheme 2:**
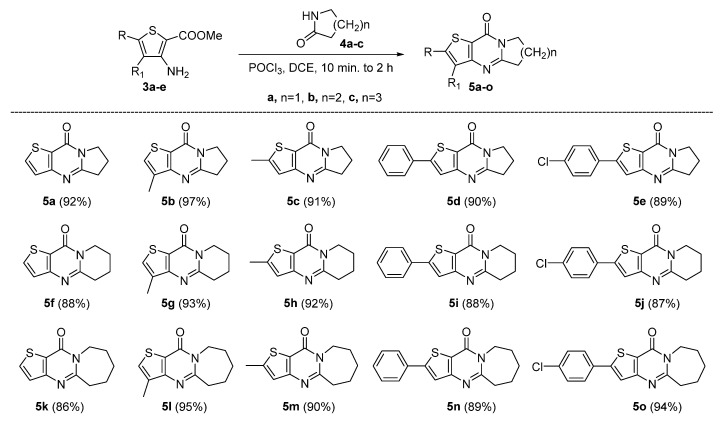
Synthetic route structures of thieno[3,2-*d*]pyrimidinones **5a–o**.

Compound **5a** was obtained with a 77% yield using dry dioxane as the solvent, but only after 8 h of reflux. A very low yield of 28% was observed if toluene was used (entry 4, Table 1). We were delighted to find that the desired cyclization product, **5a**, could be isolated with a yield of 68% (entry 5, Table 1) if the annulation reaction was carried out without solvent at 100 °C. The highest yield of the desired product (81%) was achieved after refluxing the substrate and reagent for 2 h in the absence of a solvent at 140 °C. However, the workup procedure was complicated. We preferred entry 2, which used DCE as the solvent. The product formed after only 10 min. However, we decided to maximize the yield by using a reaction time of 3–4 h. Thus, cyclization of **3a-e** to produce **5a-o** was performed under the conditions of Table 1, entry 2 (Scheme 2).

**Scheme 3:**
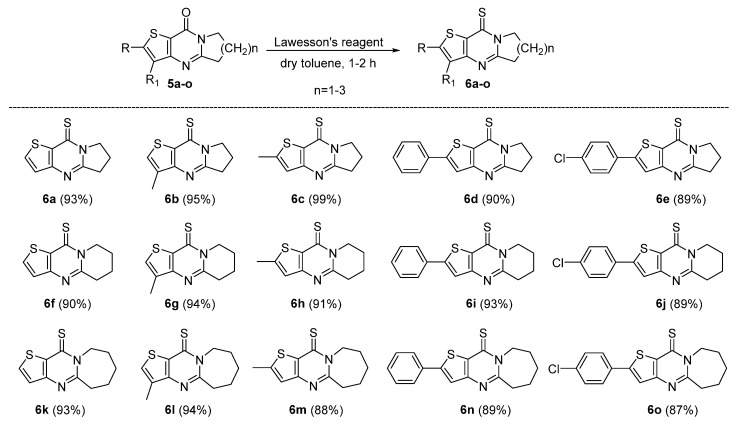
Synthetic route structures of thieno[3,2-*d*]pyrimidin-thiones **6a–o**.

An important area of medicinal and organic chemistry research involves thiones [58], which are heterocyclic compounds that form the backbone of more complex organic molecules [59]. These molecules often utilize other sites for attachment to specific types of rapidly forming molecules. We studied the formation of thiones by converting the C=O group into the C=S functional group. Pyrimidinones **5a-o** were subjected to a 2 h reflux in toluene in the presence of Lawesson’s reagent [25], resulting in the conversion of the oxygen in the carbonyl group to sulfur. Under these conditions, products **6a-o** were obtained in 84-92% yield (Scheme 3).

**Scheme 4:**
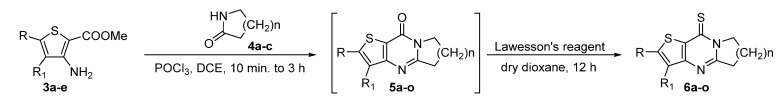
Direct one-pot synthetic route of thieno[3,2-*d*]pyrimidin-thiones **6a-o** from thiophenes **3a-e**.

The straightforward, one-pot synthesis of **6a-o** was examined (see Scheme 4). Thiophenes **3a-e** undergo condensation with lactams and POCl_3_; in dioxane to form intermediates **5a-o** after 3–4 h. Adding Lawesson’s reagent to the mixture and reacting it for an additional 2–3 h yields thiones **6a-o** in satisfactory amounts (88–99%).

### 2.2. Antiproliferative Activity

**Scheme 5:**
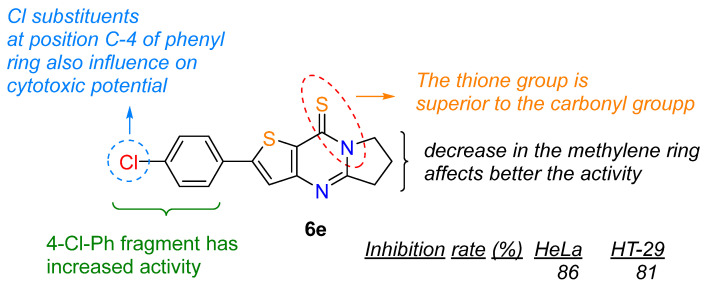
The structure–activity relationship analysis of compound **6e**.

**Figure 3:**
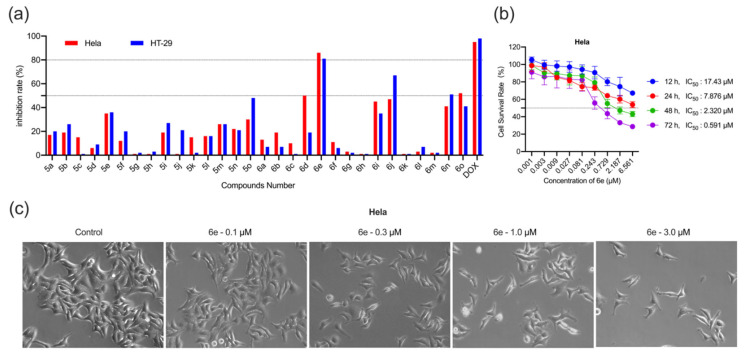
Antiproliferative activity of compounds **5a–o** and **6a–o** towards HeLa and HT-29 cell lines as measured by the MTT assay. (**a**) Cells were treated with the compounds at a fixed concentration of 5.0 μM for 48 h and the inhibition rate was calculated. For comparison, doxorubicin (DOX) was used as positive control drug. (**b**) HeLa cells were treated with **6e** at concentrations from 0 to 6.561 μM for 12, 24, 48, and 72 h, respectively, and the IC_50_ values were calculated. IC_50_ = Compound concentration required to inhibit cell growth by 50 %, was calculated with GraphPad Prism 9.0, by non-linear regression of the log-transformed data (X − Log[X]), with the Log (inhibitor) vs. normalized response–variable slope tool. Data are expressed as the mean ± SD, from dose–response curves of at least three independent experiments. (**c**) Morphological changes in HeLa cells after treatment with **6e** for 24 h. Shown are representative images photograph obtained using an inverted fluorescence microscope.

Known and novel thieno[3,2-*d*]pyrimidines (**5a-o** and **6a-o**) were evaluated for their antiproliferative activity against two human cancer cell lines (HeLa, cervical; and HT-29, colon) using the MTT assay [60]. Doxorubicin (DOX) [61] served as a positive control. The cell lines were treated with **5a-o** and **6a-o** at a concentration of 5.0 μM. Cell growth was observed following a 48-h incubation period in the presence of the compounds. The inhibition rates of compounds **5a-o** and **6a-o** are depicted in Figure 3a. Several compounds, including pyrimidinone **5o** and six pyrimidine-thiones (**6d**, **6e**, **6i**, **6j**, **6n**, and **6o**) exhibited significant activity against the HeLa and HT-29 cell lines. These compounds showed inhibition rates ranging from 47% to 86%. Specifically, pyrimidine thione **6e** demonstrated an impressive 86% inhibition of the HeLa cell line, while **6j** exhibited a 67% inhibitory effect, making it the most active derivative against the HT-29 cell line. These two thiones both incorporate a 4-chlorophenyl substituent in the thiophene ring, highlighting the importance of this fragment for antiproliferation in the thieno[3,2-*d*]pyrimidine system. Conversely, methyl substitutions in the aromatic portion of the thieno[3,2-*d*]pyrimidine series (**5g**, **5h**, **6g**, **6h**, and **6k**) showed no inhibitory effect. Additionally, adding a second sulfur atom to the thieno[3,2-d]pyrimidine framework produced more antiproliferative thieno[3,2-*d*]pyrimidine-thiones. Structure–activity relationship analysis (Scheme 5) revealed that compound **6e** exhibited a significantly higher inhibitory effect on the proliferation of HeLa and HT-29 cells. It has 86% and 81% inhibition rates, respectively, approaching the inhibition rate of the positive control, DOX. This warrants further investigation.

Thus, the HeLa cell line was selected for further biological studies because compound **6e** exhibited the most potent antiproliferative activity against HeLa cells. The dosage and exposure time of a compound can indicate its affinity for its target and, ultimately, its efficacy. Therefore, we investigated the dose- and time-dependent effects of **6e** on HeLa cell proliferation using the MTT assay. The cells were treated with various concentrations of compound **6e** (0, 0.001, 0.003, 0.009, 0.027, 0.081, 0.243, 0.729, 2.187, and 6.561 μM) for different periods (12, 24, 48, and 72 h). As shown in Figure 3b, compound **6e** inhibited HeLa cell proliferation in a concentration- and time-dependent manner. It demonstrated strong inhibitory activity, with IC_50_ value of 0.591 μM after treatment for 72 h. The selectivity index (SI) was then calculated as the ratio of the IC_50_ values for HEK-293 and HeLa cells, as shown in Table S1 (see Supplementary Materials). The IC_50_ of **6e** was 2.32 μM after 24 h of treatment. This concentration (>IC_50_ and <IC_50_) was selected for subsequent experiments.

#### 2.2.1. Effect of **6e** on Morphological Changes in HeLa Cells

Cell shrinkage [62], nuclear fragmentation [63], and chromatin condensation [64] are characteristic features of apoptosis [65,66]. We examined morphological changes in HeLa cells after 24 h treatment with **6e** and captured images with an inverted fluorescence microscope. Figure 3c shows untreated cells displayed normal morphology with tight intercellular junctions and an intact cell membrane without nuclear condensation. In contrast, HeLa cells exposed to **6e** at concentrations of 0.1 and 0.3 μM exhibited minimal effects, showing slight cell shrinkage. However, cells treated with 1 μM of **6e** exhibited varying degrees of morphological alterations and lost their original appearance. Furthermore, cells treated with **6e** at 3.0 μM exhibited the most significant morphological alterations, characterized by highly condensed nuclear dots known as apoptotic bodies. There was also a gradual reduction in cell numbers. Overall, the results regarding nuclear morphological changes indicate that the **6e** treatment triggered apoptosis in HeLa cancer cells.

#### 2.2.2. Effect of **6e** on Colony Formation, Wound Healing, and Migration Ability of HeLa Cells

Metastasis is usually identified in the advanced stages of cancer and often results in death [67,68]. According to the conventional view, tumor cell migration begins with a single tumor cell that undergoes a series of complex steps to reach and survive in distant tissues and organs [69]. This intricate, multiphase process is inefficient and involves escaping primary cancer cells into the bloodstream [70]. It culminates in colonization and growth in distant organs [71]. To evaluate the impact of **6e** on cell migration, experiments were conducted employing wound healing (scratch) and cell migration (Transwell) methodologies.

**Figure 4:**
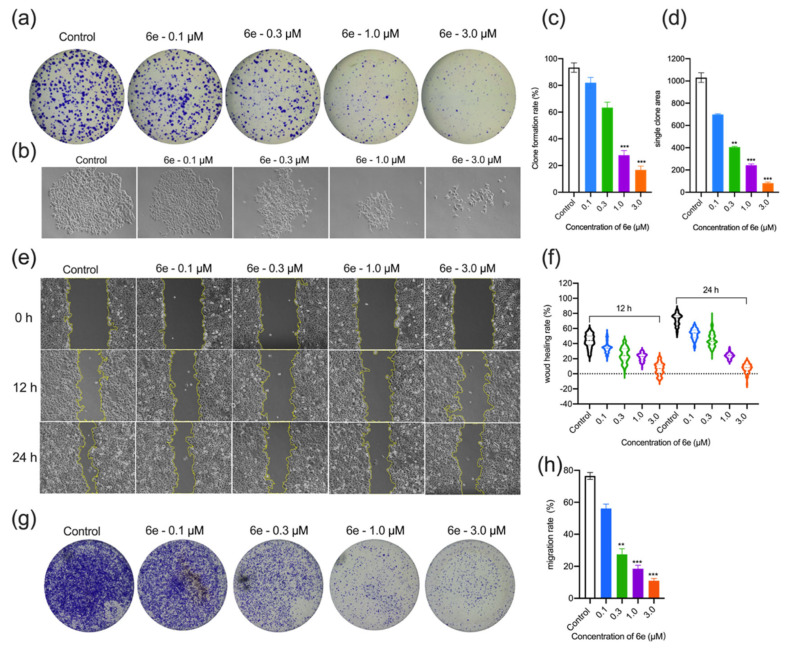
Effect of **6e** on colony formation, wound healing, and migration activity of HeLa cells. (**a**) Colony formation rate in HeLa cells after treatment with **6e**. Cells were treated with **6e** for two weeks. Representative photograph were obtained using a SMZ25 stereomicroscope (Nikon, Tokyo, Japan). (**b**) Single colony formation area in HeLa cells after treatment with **6e**. Representative photograph were obtained using a DMi8- Leica microscope. (**c**,**d**) Quantitative assessment of colony formation rate and single colony area was performed using Image J software (https://imagej.net/ij/, accessed on 14 July 2025). (**e**) Wound healing assay; HeLa cells were seeded on 6 well plate, grown to confluence and scratches made at experimental time zero, cells treated with compound **6e** (0, 0.3, 1.0, and 3.0 μM), representative photograph of initial and final scratches captured using a stereomicroscope (DMi8- Leica microscope (magnification: 100×); (**f**) Quantitative assessments were performed using ImageJ software and calculated of wound width. (**g**) Transwell cell migration assay; microscopic images of HeLa cell migration after treated with **6e** (0, 0.3, 1.0, and 3.0 μM) for 24 h: The cells migrated through the membrane were fixed, stained, and representative photographs were taken using a stereomicroscope (Nikon smz25, magnification: 5×); (**h**) Quantitative assessment of the migration rate quantified by measuring the absorbance at OD600 nm after acetic extraction and staining with crystal violet. Data are presented as the mean ± SD of three independent experiments and statistics generated with an unpaired *t*-test (GraphPad Prism soft); ** *p* < 0.01, *** *p* < 0.001 vs. control. The maximum concentration of 3 µM was chosen as a sub-toxic dose (below the 24 h IC_50_ of 7.876 µM) to assess anti-proliferative and anti-migratory effects without interference from non-specific toxicity.

The colony formation assay is a widely recognized approach to semi-quantitatively assess anticancer agents’ inhibitory effects on tumor cells’ anchorage-independent growth [72]. Another colony formation assay was then conducted to examine the inhibitory potential of **6e** on cancer cell proliferation. HeLa cells were exposed to varying concentrations of **6e** (0, 0.1, 0.3, 1.0, and 3.0 μM) for two weeks. The control group exhibited robust cell growth and colony formation, achieving 93.3% (Figure 4a,c). In contrast, the colony formation rates for the **6e** treatment groups (0.1, 0.3, 1.0, and 3.0 μM) were 82%, 63.3%, 27.7%, and 16.7%, respectively. The colony formation area decreased progressively as the treatment concentrations increased (Figure 4b,d). **6e** markedly inhibited the colony formation of HeLa cells in a dose-dependent manner, indicating its potential to disrupt the colony-forming capability of cancer cells.

HeLa cells were seeded in six-well culture plates for the wound healing assay and incubated overnight to allow them to grow to 90–95% confluence. Then, a wound (scratch) was created using a pipette tip, and images were taken with a microscope. The cells were then treated with different concentrations of **6e** (0 μM, 0.1 μM, 0.3 μM, 1.0 μM, and 3.0 μM) for 0, 12, or 24 h while incubating at 37 °C and 5% CO_2_. As shown in Figure 4e, the wound was imaged at 0, 12, and 24 h. Quantitative analysis was performed based on wound healing distance, resulting in wound healing rate calculations. The DMSO control group exhibited 50% and 80% wound healing rates after 12 and 24 h, respectively. In contrast, the **6e** treatment group showed wound healing rates of 32.4%, 22.7%, 20%, and 9% after 12 h and 52%, 40%, 21%, and 6.7% after 24 h (see Figure 4f). These results suggest that **6e** can inhibit the wound healing ability of HeLa cells in a dose- and time-dependent manner.

Additionally, Transwell migration assays were conducted to evaluate the impact of **6e** on HeLa cell migration. Twenty-four-well inserts were used for this purpose. After treating the cells with **6e** for 24 h, the cells were fixed, stained, and photographed (Figure 4g). Then, 10% acetic acid (100 μL/well) was added for extraction over 10 min. The cells were then transferred to 96-well plates and read at an optical density (OD) of 600 nm using a microplate reader. The control group’s cell migration ability was significantly better compared to the experimental groups, whose number of cells passing through the membrane decreased progressively. The migration rate of the control group was 76.5%. In contrast, the migration rates of the experimental group (0.1, 0.3, 1.0, and 3.0 μM) were reduced to 56%, 27.5%, 18.5%, and 11%, respectively (Figure 4h). These results suggest that compound **6e** inhibited the migration of HeLa cells in a dose-dependent manner.

#### 2.2.3. Cell-Cycle Arrest, Microtubule Structures, Intracellular ROS Accumulation, and Apoptosis in HeLa Cells by **6e**

**Figure 5:**
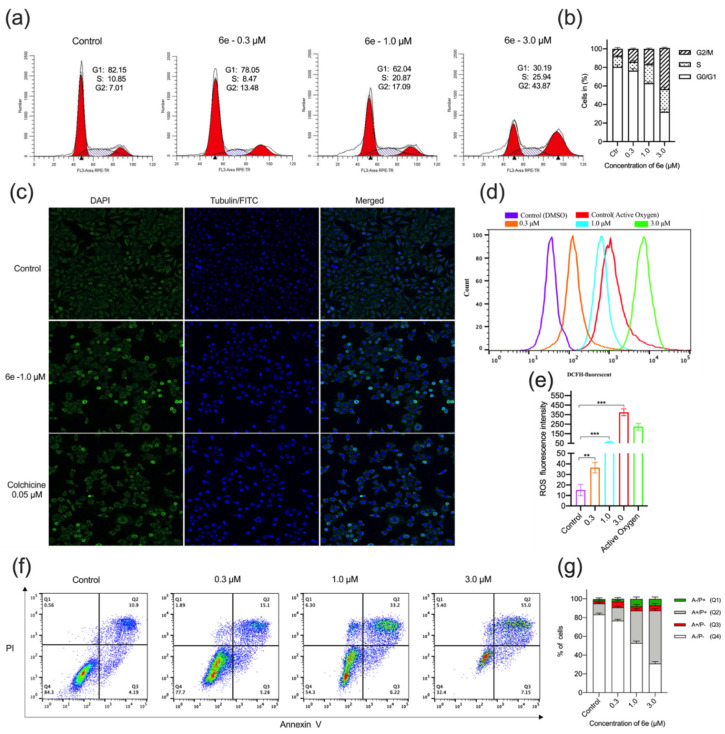
Effects of **6e** on cell cycle arrest, microtubule network, ROS accumulation, and apoptosis in HeLa cells. (**a**) Cell cycle analysis. Cells were treated with **6e** for 24 h with the indicated concentrations, then stained with propidium iodide (PI) and examined by flow cytometry. The percentage of cell phase of each group were analyzed by ModFit software (version 6.0). (**b**) Quantitative evaluation. Representative histograms depict the cell cycle distribution in HeLa cell after treated with **6e**, Data presented as mean ± SD, at least three independent experiments. (**c**) effects of **6e** on microtubule networks in HeLa cells. Cells were treated for 24 h with 6e (1.0 µM) or colchicine (0.05 µM), fixed, and stained with DAPI (blue, nuclei) followed by an anti-tubulin antibody (green, microtubules) to assess polymerization status. Images were obtained by laser scanning confocal microscopy at the same magnification. (**d**) Observation of ROS levels of 2,7-dichlorodihydrofluorescein diacetate (DCFH-DA) using flow cytometry. HeLa cells were treated with **6e** for 24 h and stained with DCFH-DA. (**e**) Flow cytometric results were quantified using the median value of ROS measurements. (**f**) Cell apoptosis effects of **6e** on Hela cells. Apoptosis was determined using flow cytometry after treatment of HeLa cells with **6e** for 24 h. Representative images are shown using FlowJO software (version 11). Cells were stained annexin V-FITC/PI, PI, propidium iodide; FITC, Fluorescein isothiocyanate; cells in quadrant Q1 are necrotic, Q2 late apoptotic, Q3 early apoptotic, and Q4 living. (**g**) Percentage of apoptotic HeLa cells. Data represent the mean ± SD of three independent experiments. ** *p* < 0.01, *** *p* < 0.001 vs. control. The highest concentration of 3 µM was selected as a sub-toxic level. This allowed reliable detection of cell-cycle and apoptosis changes without confounding effects from high-concentration cytotoxicity.

Excessive growth is a well-known trait of cancer cells [73]. Dysregulation of the cell cycle is a defining characteristic of tumor cells [74]. Therefore, agents that can halt the cycle of cancer cells are effective anticancer agents [75,76]. To explore the mechanisms behind the antiproliferative effects of **6e**, we analyzed its influence on cell cycle phase distribution using flow cytometry. HeLa cells were treated with different concentrations of **6e** (0.0, 0.3, 1.0, and 3.0 μM) and incubated for 24 h. Subsequently, we conducted DNA profile analysis using flow cytometry. The results (Figure 5a,b) indicated that compound **6e** led to a dose-dependent increase in cells in the G2/M phase, while most control cells remained in the G0/G1 phase. Following treatment with different concentrations of **6e**, the percentage of cells in the G2/M phase increased from 12.01% in the control group to 19.48%, 21.09%, and 40.87% in the 0.3, 1.0, and 3.0 μM groups, respectively. Conversely, the percentage of cells in the G0/G1 phase decreased from 82.15% in the control group to 78.05%, 62.04%, and 30.19% in the 0.3, 1.0, and 3.0 μM groups, respectively. Thus, **6e** significantly induced cell cycle arrest at the G2/M phase, ultimately delaying progression of the cell cycle and inhibiting HeLa cell proliferation.

Tubulin is essential for maintaining cell shape and function [77,78]. Since **6e** affects cell cycle parameters by causing a G2/M blockade, it may also affect tubulin assembly. To investigate whether the antiproliferative effects of **6e** are related to its interaction with the microtubule system, we examined the cellular microtubule network in HeLa cells treated with **6e** for 12 h. Colchicine was used as a reference compound, and DNA was stained blue and α-tubulin green. The experiment utilized an immunofluorescence assay. Figure 5c shows that control HeLa cells display regular microtubule arrangement and organization. In contrast, HeLa cells treated with **6e** (1.0 μM) and colchicine (0.05 μM) exhibited an incomplete microtubule network. Cell shapes varied from spindle-shaped to round. Microtubules clustered around the nucleus, preventing it from undergoing proper mitosis. Thus, **6e** inhibited microtubule assembly and disrupted the cytoskeleton, similar to colchicine. These results confirmed that **6e** selectively targeted the G2/M phase of the cell cycle, consistent with the behavior of tubulin assembly inhibitors.

Reactive oxygen species (ROS) [79] have been linked to numerous human ailments, including chronic inflammation and cancer [80]. ROS also play a crucial role in various biological processes, including cell survival, growth, proliferation, and immune response. We measured ROS levels in HeLa cells after treating them with **6e**. We used flow cytometry to assess the fluorescence intensity of DCFH-DA [81], which reflects the extent of ROS production induced by **6e**. The fluorescence intensity indicates ROS production. HeLa cells were exposed to **6e** at concentrations of 0, 0.3, 1.0, and 3.0 μM for 24 h. The fluorescent probe DCFH-DA was used to analyze the impact of **6e** on ROS levels in the cells. As illustrated in Figure 5d,e, the fluorescence intensity in cells treated with **6e** significantly exceeded that of the control group, demonstrating that **6e** promotes ROS generation in HeLa cells. Specifically, the fluorescence intensity in HeLa cells treated with **6e** at concentrations of 0, 0.3, 1.0, and 3.0 μM for 24 h was 2.2-, 4.9-, and 25-fold greater than that of the control group, respectively. These results suggest that **6e** increased ROS production in cells.

To investigate the mechanism of its antiproliferative effect, **6e** underwent apoptosis analysis via flow cytometry [82,83]. Cell death was assessed using two-dimensional cell sorting with propidium iodide (PI) staining of DNA and labeling of phosphatidylserine (PS) with a fluorescent annexin V derivative. This dual staining approach distinguishes between four types of cells: unaffected (annexin V−/PI−), early apoptotic (annexin V+/PI−), late apoptotic (annexin V+/PI+), and necrotic (annexin V−/PI+). HeLa cells were treated with **6e** concentrations of 0.0, 0.3, 1.0, and 3.0 μM for 24 h. Untreated cells served as the control. Prior to analysis, the cells were stained with annexin V-fluorescein isothiocyanate (FITC) and PI. The resulting two-parameter histograms illustrate the effects of various concentrations of **6e** on HeLa cells after 24 h. Compared with the control group, **6e** treatment significantly increased the percentage of annexin V-positive cells in a dose-dependent manner. In the experiment presented in Figure 5f,g, the rate of early apoptotic cells increased from 4.19% in the control group to 5.28%, 6.22%, and 7.15% in the treatment groups after 24 h of treatment with 6e at 0.3, 0.3, 1.0, and 3.0 μM, respectively. Similarly, the percentage of late apoptotic cells increased from 10.9% in the control group to 15.1%, 33.2%, and 55% in the treatment groups, respectively. The total percentage of apoptotic cells (the sum of early and late apoptotic cells) in the control group was 15.09%. In the groups treated with **6e** at concentrations of 0.3, 1.0, and 3.0 μM, the percentages increased to 20.38%, 39.42%, and 62.15%, respectively.

The HeLa and HCT-116 cell lines were chosen as models for cervical and colorectal carcinoma, respectively, both of which have known abnormalities in cell-cycle regulation. HeLa cells, with alterations in the CDK2/cyclin E axis, served as a suitable system to test the hypothesized actions of the synthesized compounds. Although their complex genetic background is a limitation, it allows reproducible screening of anti-proliferative effects and cell-cycle arrest. HT-29 cells were used in preliminary screening but were excluded from later assays due to lower sensitivity to the lead compound **6e**, suggesting potential tumor-type selectivity for further study.

### 2.3. Molecular Docking

In this study, we analyzed the interaction patterns of compound **6e** with potential targets via molecular docking to investigate its antitumor mechanism. The targets selected for this study were CDK2, CDK3, CDK5-p25/p35 complexes, CK1γ1, and CSK, as they play critical roles in tumor biology [84,85]. CDK2/3 regulates the cell cycle, and the abnormal activation of these kinases directly causes the uncontrolled proliferation of tumor cells [86]. The abnormal activation of CDK5-p25/p35 complexes promotes tumor cell invasion and metastasis [87]. CK1γ1 is involved in the abnormal activation of the Wnt/β-catenin signaling pathway, which drives tumorigenesis. CSK negatively regulates Src family kinase activity, and its dysfunction is closely linked to tumor cell proliferation and survival [88,89]. These targets are associated with tumor cell cycle dysregulation [90], malignant phenotype transformation, abnormal activation of signaling pathways, and pro-cancer signal transduction, respectively.

Table 2 shows the results of the molecular docking of compound **6e** with various kinase targets. It includes the PDBIDs of each target, the CDOCKER binding energies (which reflect binding strength; larger absolute negative values indicate more stable binding), and the types of binding interactions, such as hydrogen bonds and hydrophobic interactions. The table is used to evaluate the binding potential of **6e** with different targets. Compound 6e exhibits strong binding affinity and diverse interaction modes with kinase targets, including CDK2 and CDK5-p35. In particular, its binding to CDK2 is the most stable, making it a promising candidate for kinase inhibitors.

#### 2.3.1. Analysis of Protein–Protein Interactions

The complexes formed by the docked compounds and proteins were visualized using PyMOL 2.1 software. The four targets with the most negative scores were selected. The binding modes of the compounds to the proteins were obtained. Based on these modes, the amino acid residues at the interface where the compounds bind to the protein pockets can be observed.

**Figure 6:**
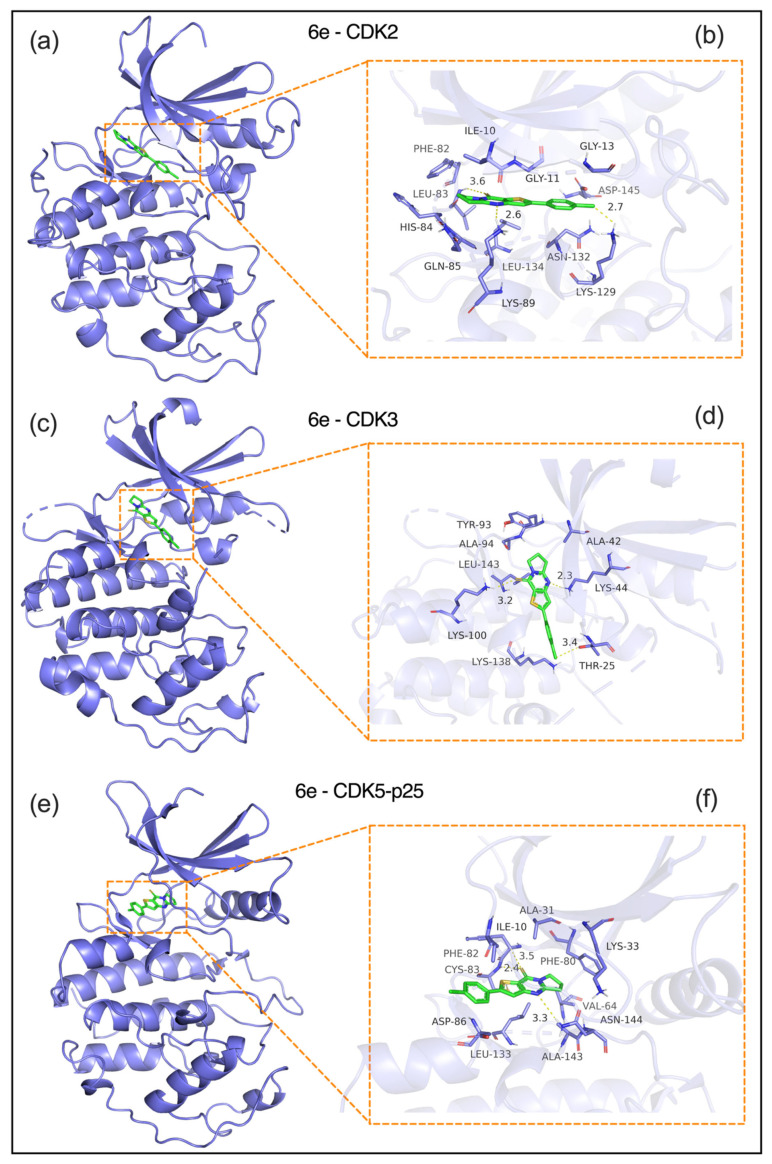
Compound **6e** is docked with CDK2, CDK3, and CDK5—p25; (**a**,**c**,**e**) show the overall docking structures of compound **6e** (green) within CDK2 (blue), CDK3 (blue), and CDK5—p25 (blue) proteins, respectively. (**b**,**d**,**f**) are enlarged views of the binding pockets, highlighting the key amino acid residues (labeled) interacting with compound **6e**. The dashed lines and numbers indicate the distances (in Å) between specific atoms of the compound and the interacting amino acids, which help to illustrate the binding mode and interaction strength.

**Figure 7:**
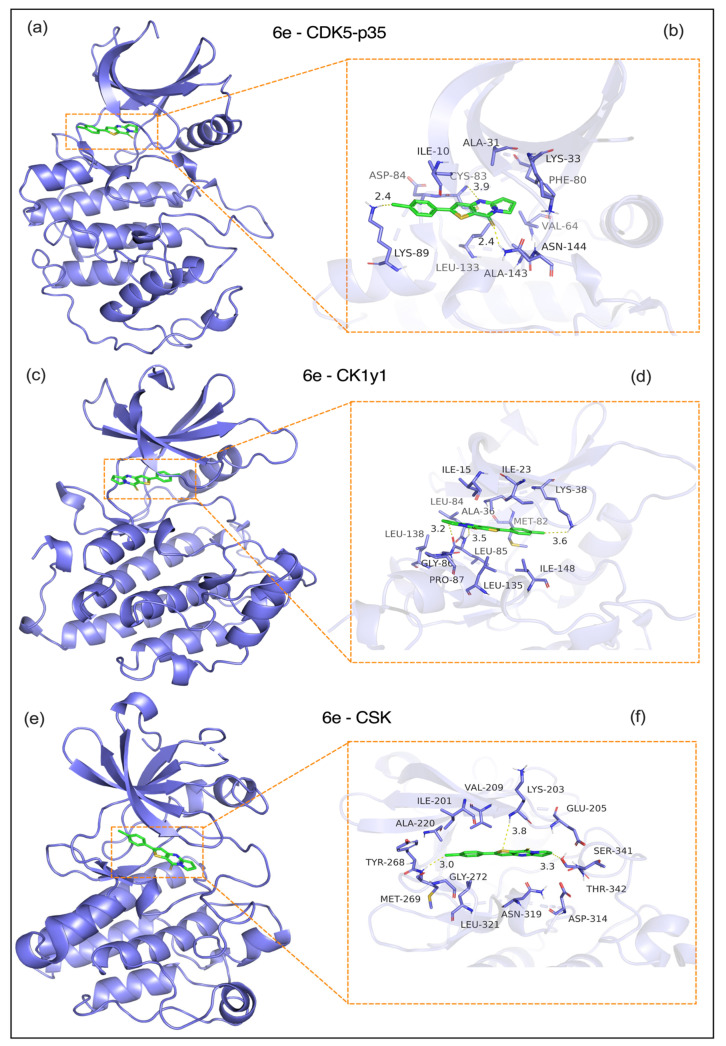
Compound **6e** is docked with CDK5—p35, CK1γ1, and CSK; (**a**,**c**,**e**) show the overall docking structures of compound **6e** (green) within CDK5—p35 (blue), CK1γ1 (blue), and CSK (blue) proteins, respectively. (**b**,**d**,**f**) are enlarged views of the binding pockets, highlighting the key amino acid residues (labeled) interacting with compound **6e**. Dashed lines and numbers indicate the distances (in Å) between specific atoms of the compound and the interacting amino acids, helping to illustrate the binding mode and interaction strength.

The molecular docking study revealed that compound **6e** exhibits distinct binding modes with key tumor-related kinases, providing structural insights into its antitumor mechanism (see Figure 6 and Figure 7):

a) CDK2 (PDB: 1H15, Figure 6a,b): **6e** was embedded in the ATP-binding pocket of CDK2, forming critical interactions and a CDOCKER energy of -8.2 kcal/mol. Hydrogen bond: A 2.6-Å bond with the ILE10 backbone carbonyl anchors the ligand. Hydrophobic interactions: Close contacts with PHE82 (2.7 Å), LEU83 (3.1 Å), and VAL104 (3.6 Å) via van der Waals forces. Electrostatic interaction: A 3.2 Å interaction with ASN132 stabilizes the ligand’s pyrimidine moiety. These interactions block CDK2-mediated phosphorylation of the Rb protein, which is consistent with the G1/S phase arrest observed in cellular assays.
b) CDK3 (PDB: 1O37, Figure 6c,d): The ligand is bound to a conserved hydrophobic pocket in CDK3 (CDOCKER energy: -7.8 kcal/mol) through the following interactions. Hydrogen bond: A 2.3 Å bond between the ligand’s purine ring and the LYS44 side chain. Hydrophobic interactions: A 3.2-Å interaction with ALA42, a 3.4-Å interaction with LEU143, and π-π stacking with TYR93 at 3.6 Å. Electrostatic interaction: A 3.1 Å interaction with THR25 enhances binding specificity. This binding mode disrupts the formation of the CDK3-cyclin E complex, thereby inhibiting G1 phase progression.
c) CDK5-p25 complex (PDB: 1UGS, Figure 6e,f): **6e** engages the ATP-binding site of the CDK5-p25 complex (CDOCKER energy: −7.6 kcal/mol) via: Hydrogen bond: A 2.4-Å bond with the ALA31 backbone amide. Hydrophobic interactions: PHE80 (3.3 Å), ILE10 (3.5 Å), and CYS82 (3.4 Å); and π-π stacking with PHE80 (3.8 Å). An interaction with PHE80’s aromatic ring (3.8 Å) is critical for ligand orientation. These interactions may interfere with CDK5-p25-mediated phosphorylation of the tau protein, which is associated with reduced metastatic potential.
d) CDK5-p35 complex (PDB: 2PH1, Figure 7a,b): **6e** showed weaker binding to the CDK5-p35 complex (CDOCKER energy: -7.2 kcal/mol) through a hydrogen bond with the backbone carbonyl of GLY30. A 2.4 Å bond with the GLY30 backbone carbonyl. Hydrophobic interactions: Hydrophobic interactions with VAL32 (3.5 Å), ALA31 (3.5 Å), ILE10 (3.3 Å), and LEU83 (3.6 Å). Notably, the p35-specific loop (residues 140–150) showed minimal contact with **6e**, suggesting a lower risk of off-target effects in neurons compared to p25.
e) CK1γ1 (PDB: 3P70, Figure 7c,d): **6e** bound to the CK1γ1 kinase domain (CDOCKER energy: -6.8 kcal/mol) via the following: Hydrogen bond: A 2.9-Å bond with the SER36 side chain, which is critical for ATP binding. Hydrophobic interactions: LEU85 (3.5 Å), ILE148 (3.6 Å), and VAL116 (3.4 Å); and electrostatic interactions: A 3.3 Å interaction with GLU63 stabilizes the ligand’s charged group. This binding mode may disrupt CK1γ1-mediated β-catenin phosphorylation and inhibit Wnt/β-catenin signaling.
f) CSK (PDB: 1A1Q, Figure 7e,f): **6e** interacts with the CSK SH2-kinase interface (CDOCKER energy: -6.5 kcal/mol) through the following. Hydrogen bond: A 2.8 Å bond with Tyr268, a key phosphorylation site. Hydrophobic interactions: ALA220 (3.3 Å), MET269 (3.8 Å), and LEU273 (3.5 Å). Electrostatic interaction: A 3.1 Å interaction with GLU222 enhances the binding affinity. These interactions may enhance CSK-mediated suppression of Src kinases, which aligns with reduced tumor cell survival.

Together, these docking results show that **6e** targets multiple cell cycle regulators and signaling kinases. This provides a structural basis for its dose-dependent antiproliferative, anti-migratory, and pro-apoptotic effects in HeLa cells. Lead compound **6e** showed strong antiproliferative effects, causing clear cell cycle arrest at both the G1/S and G2/M phases. Based on this, we thought cyclin-dependent kinases (CDKs) could be key targets, since CDK1, CDK2, CDK4, and CDK6 control these checkpoints. Cell cycle arrest often points to CDK inhibition. To support this idea, molecular docking revealed that the compound binds well to CDK2’s ATP-binding pocket, making important hydrogen bonds with Leu83 and Glu81 in the hinge region. The compound also forms hydrophobic contacts with Asp145 in the gatekeeper area. Its flat core and polar groups fit the kinase pocket, similar to other known kinase inhibitors. Molecular dynamics simulations lasting over 100 nanoseconds also demonstrated that the complex is stable. While these findings do not fully confirm CDK inhibition, they suggest a CDK-focused mechanism, which we are testing further with kinase activity assays and knockdown experiments.

#### 2.3.2. Molecular Dynamics Analysis

**Figure 8:**
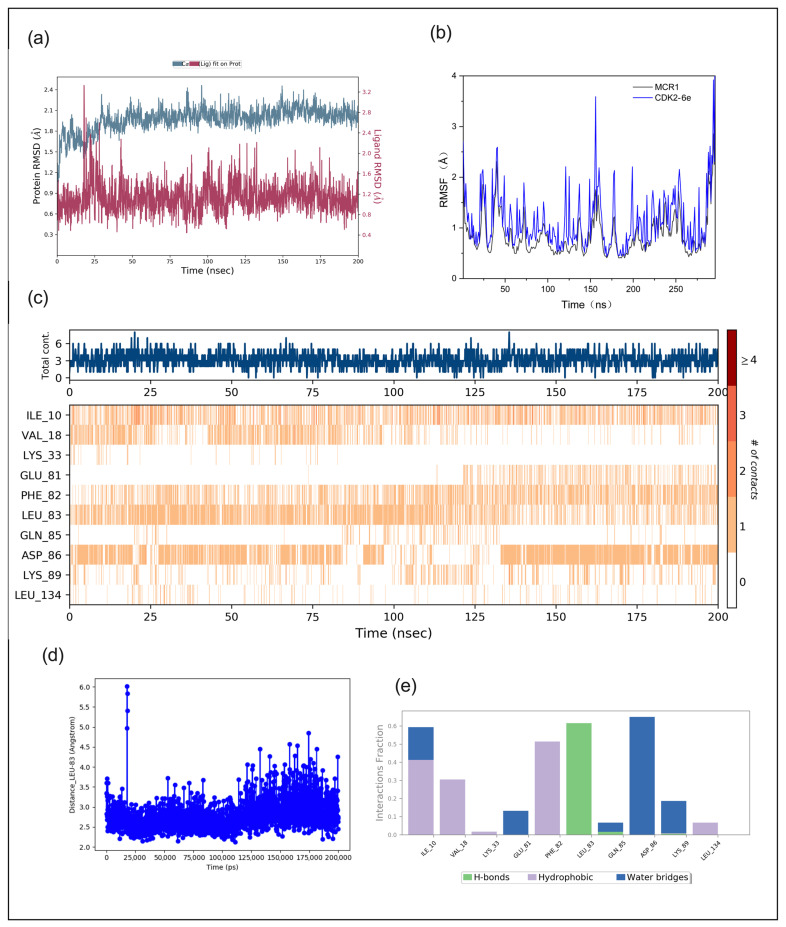
Molecular dynamics analysis of compound **6e** with CDK2: (**a**) The root mean square deviation (RMSD) plot shows fluctuations in the protein backbone (Cα, blue) and the ligand (red) over time. (**b**) The root mean square fluctuation (RMSF) plot compares RMSF values for the MCR1 system (gray) and the CDK2-**6e** complex (blue). (**c**) The total contact number curve represents variations in contact over time. The residue contacts map shows the frequency of interactions between specific amino acids (e.g., ILE_10 and VAL_18) and the ligand. (**d**) The distance variation plot shows changes in distance related to LEU_83 (in angstroms over time). (**e**) The interaction proportion bar chart categorizes interactions as hydrogen bonds (green), hydrophobic interactions (purple), or water bridges (blue).

We performed a dynamic analysis to investigate the changes in dynamic stability and interaction that occur after compound **6e** binds to CDK2. According to the results of the molecular dynamics simulation, the complex formed by compound **6e** and CDK2 has a stable structure and favorable interactions. The RMSD curves (Figure 8a) show that the CDK2 protein undergoes conformational adjustment in the early stage, after which it reaches a dynamic equilibrium. Meanwhile, ligand compound **6e** completes positional and orientational adjustments in the early binding stage. Both show stable fluctuations in the later stage, indicating the complex’s stable structure. The RMSF curves (Figure 8b) show that the CDK2–**6e** system exhibits drastic atomic fluctuations; the flexible regions may affect the sustainability of ligand binding. The total contact number curve (Figure 8c) and the residue contact matrix (Figure 8c) indicate dynamic changes in interactions within the system. Hydrophobic residues, such as ILE_10, VAL_18, and LEU_83, are critical for binding and maintain strong interactions with the ligand. In contrast, GLN_85 shows weak and discontinuous interactions. The distance-related curve (Figure 8d) for LEU_83 remains stable for most of the time; however, fluctuations during specific periods suggest changes in the binding state. In terms of interaction proportions (Figure 8e), hydrophobic interactions dominate, followed by hydrogen bonds; water bridges play a minor role. Overall, this complex is suitable for optimization as a lead compound. It is worth noting that we selected compound **6e** for molecular dynamics simulation because it exhibited vigorous anti-proliferative activity and appeared to have an effect on cell cycle arrest. To demonstrate its potential, we evaluated its stability and main interactions within the CDK2 binding pocket, offering early structural support for our proposed target. We plan to run similar molecular dynamics simulations with other analogs or reference inhibitors in future work.

## 3. Materials and Methods

### 3.1. Chemistry

#### 3.1.1. General Materials and Methods

Melting points were determined using a BUCHI Melting Point B-540 apparatus (Büchi Labortechnik, AG, Flawil, Switzerland) and were uncorrected. The NMR spectra were recorded on a Varian 400 NMR spectrometer (Varian, Inc., CA, USA), (400 MHz for ^1^H and 100 MHz for ^13^;C) using CDCl_3_; as the solvent and TMS as the internal standard in the ^1^H NMR spectra. All reagents and solvents were purchased from Tokyo Chemical Industry Co., Ltd. (Tokyo, Japan), Shaanxi United Energy Chemical Technology Co. (Shaanxi, China), Shanghai Qinghang Chemical Co., Ltd. (Shanghai, China), and Sigma-Aldrich (Shanghai, China), and were used without further purification. High-resolution mass spectra (HRMS) were recorded using a UHPLC-Q-Orbitrap-MS (Thermo Fisher Scientific, Shanghai, China).

#### 3.1.2. General Procedure for the Synthesis of Compounds **5a–5o**

POCl_3_ (2.3 mL, 25 mmol) was added dropwise to a solution of 3-amino-thiophene-2-carboxylates (**3a–3e**) (10 mM) and the appropriate lactam (12 mM) in DCE (20 mL). The reaction mixture was then refluxed at 80 °C for 2 h. The solvent and excess POCl_3_ were evaporated under reduced pressure, and the yellow solid was suspended in DCM (100 mL); NH_4_OH (10%) was added to achieve a pH of 9. The mixture was extracted with DCM (2 × 30 mL), and the organic phase was washed with brine. The organic layer was separated, dried over anhydrous Na_2_SO_4_, filtered, and concentrated under reduced pressure to yield the crude product, which was purified by silica gel chromatography to produce the pure corresponding compounds (**5a–5o**).

**6,7-Dihydropyrrolo[1,2-*a*]thieno[3,2-*d*]pyrimidin-9(5*H*)-one (5a)**. Yield 92%, white solid, mp 154–155 °C; ^1^H NMR (400 MHz, CDCl_3_) δ 7.76 (d, *J* = 5.2 Hz, 1H), 7.26 (d, *J* = 5.2 Hz, 1H), 4.23 (t, *J* = 7.3 Hz, 2H), 3.19 (t, *J* = 7.9 Hz, 2H), 2.39–2.27 (m, 2H). ^13^C NMR (100 MHz, CDCl_3_) δ 161.46, 158.40, 157.40, 134.14, 124.80, 121.46, 46.68, 32.33, 20.10. HRMS (ESI) calcd for C_9_H_8_N_2_OS[M+H]^+^ 193.0421, found 193.0426.
**3-Methyl-6,7-dihydropyrrolo[1,2-*a*]thieno[3,2-*d*]pyrimidin-9(5*H*)-one (5b)**. Yield 97%, white solid, mp 179–180 °C; ^1^H NMR (400 MHz, CDCl_3_) δ 7.40 (q, *J* = 1.1 Hz, 1H), 4.22 (t, *J* = 7.3 Hz, 2H), 3.21 (t, *J* = 8.0 Hz, 2H), 2.38 (s, 3H), 2.37–2.25 (m, 2H). ^13^C NMR (100 MHz, CDCl_3_) δ 161.30, 158.78, 157.00, 149.85, 122.93, 119.96, 46.70, 32.39, 20.03, 16.92. ^13^C NMR (100 MHz, CDCl_3_) δ 161.08, 157.62, 157.35, 133.72, 129.34, 121.57, 46.60, 32.47, 20.22, 13.26. HRMS (ESI) calcd for C_10_H_10_N_2_OS[M+H]^+^ 207.0578, found 207.0582.
**2-Methyl-6,7-dihydropyrrolo[1,2-*a*]thieno[3,2-*d*]pyrimidin-9(5*H*)-one (5c)**. Yield 91%, white solid, mp 172–174 °C; ^1^H NMR (400 MHz, CDCl_3_) δ 6.92 (q, *J* = 1.1 Hz, 1H), 4.20 (t, *J* = 7.3 Hz, 2H), 3.16 (t, *J* = 7.9 Hz, 2H), 2.60 (d, *J* = 1.1 Hz, 3H), 2.36–2.24 (m, 2H). ^13^C NMR (100 MHz, CDCl_3_) δ 161.09, 158.58, 156.80, 149.65, 122.72, 119.76, 46.50, 32.18, 19.82, 16.71. HRMS (ESI) calcd for C_10_H_10_N_2_OS[M+H]^+^ 207.0575, found 207.0582.
**2-Phenyl-6,7-dihydropyrrolo[1,2-*a*]thieno[3,2-*d*]pyrimidin-9(5*H*)-one (5d)**. Yield 90%, light yellow solid, mp 205–206 °C; ^1^H NMR (400 MHz, CDCl_3_) δ 7.73–7.67 (m, 2H), 7.48–7.37 (m, 4H), 4.24 (t, *J* = 7.3 Hz, 2H), 3.19 (t, *J* = 8.0 Hz, 2H), 2.38–2.28 (m, 2H). ^13^C NMR (100 MHz, CDCl_3_) δ 161.72, 158.96, 157.23, 152.51, 133.48, 129.63, 129.38, 126.60, 120.70, 120.23, 46.83, 32.48, 20.07. HRMS (ESI) calcd for C_15_H_12_N_2_OS[M+H]^+^ 269.0743, found 269.0745.
**2-(4-Chlorophenyl)-6,7-dihydropyrrolo[1,2-*a*]thieno[3,2-*d*]pyrimidin-9(5*H*)-one (5e)**. Yield 89%, light yellow solid, mp 242–244 °C; ^1^H NMR (400 MHz, CDCl_3_) δ 7.66–7.58 (m, 2H), 7.45–7.38 (m, 3H), 4.24 (t, *J* = 7.3 Hz, 2H), 3.19 (t, *J* = 8.0 Hz, 2H), 2.38–2.27 (m, 2H). ^13^C NMR (100 MHz, CDCl_3_) δ 161.89, 158.91, 157.12, 150.95, 135.59, 131.99, 129.57, 127.77, 120.89, 120.57, 46.86, 32.47, 20.04. HRMS (ESI) calcd for C_15_H_11_ClN_2_OS[M+H]^+^ 303.0353, found 303.0357.
**7,8-Dihydro-5*H*-pyrido[1,2-*a*]thieno[3,2-*d*]pyrimidin-10(6*H*)-one (5f)**. Yield 88%, white solid, mp 103–105 °C; ^1^H NMR (400 MHz, CDCl_3_) δ 7.74 (d, *J* = 5.3 Hz, 1H), 7.24 (d, *J* = 5.2 Hz, 1H), 4.11 (t, *J* = 6.1 Hz, 2H), 3.01 (t, *J* = 6.7 Hz, 2H), 2.08–1.90 (m, 4H). ^13^C NMR (100 MHz, CDCl_3_) δ 158.64, 156.59, 156.38, 134.31, 124.75, 121.14, 42.41, 31.87, 22.26, 19.48. HRMS (ESI) calcd for C_10_H_10_N_2_OS[M+H]^+^ 207.0587, found 207.0591.
**3-Methyl-7,8-dihydro-5*H*-pyrido[1,2-*a*]thieno[3,2-*d*]pyrimidin-10(6*H*)-one (5g)**. Yield 93%, white solid, mp 99 °C; ^1^H NMR (400 MHz, CDCl_3_) δ 7.37 (d, *J* = 1.2 Hz, 1H), 4.11 (t, *J* = 6.1 Hz, 2H), 3.03 (t, *J* = 6.7 Hz, 2H), 2.38 (d, *J* = 1.1 Hz, 3H), 2.05–1.91 (m, 4H). ^13^C NMR (100 MHz, CDCl_3_) δ 158.89, 156.23, 155.37, 133.75, 129.35, 121.24, 42.23, 31.99, 22.29, 19.53, 13.11. HRMS (ESI) calcd for C_11_H_12_N_2_OS[M+H]^+^ 221.0743, found 221.0745.
**2-Methyl-7,8-dihydro-5*H*-pyrido[1,2-*a*]thieno[3,2-*d*]pyrimidin-10(6*H*)-one (5h)**. Yield 92%, white solid, mp 97–98 °C; ^1^H NMR (400 MHz, CDCl_3_) δ 6.89 (d, *J* = 1.1 Hz, 1H), 4.08 (t, *J* = 6.1 Hz, 2H), 2.98 (t, *J* = 6.7 Hz, 2H), 2.59 (d, *J* = 1.2 Hz, 3H), 2.06–1.88 (m, 4H). ^13^C NMR (100 MHz, CDCl_3_) δ 158.13, 156.89, 156.51, 150.02, 122.80, 119.81, 42.34, 31.84, 22.29, 19.52, 16.90. HRMS (ESI) calcd for C_11_H_12_N_2_OS[M+H]^+^ 221.0743, found 221.0745.
**2-Phenyl-7,8-dihydro-5*H*-pyrido[1,2-*a*]thieno[3,2-*d*]pyrimidin-10(6*H*)-one (5i)**. Yield 88%, light yellow solid, mp 166–167 °C; ^1^H NMR (400 MHz, CDCl_3_) δ 7.74–7.66 (m, 2H), 7.48–7.37 (m, 4H), 4.11 (t, *J* = 6.1 Hz, 2H), 3.01 (t, *J* = 6.7 Hz, 2H), 2.08–1.90 (m, 4H). ^13^C NMR (100 MHz, CDCl_3_) δ 158.35, 156.99, 156.94, 152.60, 133.52, 129.62, 129.37, 126.64, 120.44, 120.09, 42.46, 31.91, 22.29, 19.51. HRMS (ESI) calcd for C_16_H_14_N_2_OS[M+H]^+^ 283.0900, found 283.0903.
**2-(4-Chlorophenyl)-7,8-dihydro-5*H*-pyrido[1,2-*a*]thieno[3,2-*d*]pyrimidin-10(6*H*)-one (5j)**. Yield 87%, light yellow solid, mp 190–192 °C; ^1^H NMR (400 MHz, CDCl_3_) δ 7.66–7.58 (m, 2H), 7.45–7.35 (m, 3H), 4.11 (t, *J* = 6.1 Hz, 2H), 3.01 (t, *J* = 6.7 Hz, 2H), 2.08–1.91 (m, 4H). ^13^C NMR (100 MHz, CDCl_3_) δ 158.25, 157.11, 156.95, 151.05, 135.58, 132.03, 129.56, 127.81, 120.62, 120.45, 42.50, 31.90, 22.26, 19.48. HRMS (ESI) calcd for C_16_H_13_ClN_2_OS[M+H]^+^ 317.0510, found 317.0515.
**6,7,8,9-Tetrahydrothieno[3′,2′:4,5]pyrimido[1,2-*a*]azepin-11(5*H*)-one (5k)**. Yield 86%, white solid, mp 111–112 °C; ^1^H NMR (400 MHz, CDCl_3_) δ 7.73 (d, *J* = 5.2 Hz, 1H), 7.24 (d, *J* = 5.3 Hz, 1H), 4.42 (t, *J* = 4.7 Hz, 2H), 3.08 (t, *J* = 5.8 Hz, 2H), 1.90–1.76 (m, 6H). ^13^C NMR (100 MHz, CDCl_3_) δ 161.51, 158.41, 156.26, 134.36, 125.04, 121.53, 42.85, 37.65, 29.76, 27.98, 25.38. HRMS (ESI) calcd for C_11_H_12_N_2_OS[M+H]^+^ 221.0743, found 221.0748.
**3-Methyl-6,7,8,9-tetrahydrothieno[3′,2′:4,5]pyrimid[1,2-*a*]azepin-11(5*H*)-one (5l)**. Yield 95%, white solid, mp 113 °C; ^1^H NMR (400 MHz, CDCl_3_) δ 7.36 (q, *J* = 1.0 Hz, 1H), 4.41 (t, *J* = 4.9 Hz, 2H), 3.10 (t, *J* = 5.8 Hz, 2H), 2.37 (d, *J* = 1.1 Hz, 3H), 1.91–1.76 (m, 6H). ^13^C NMR (100 MHz, CDCl_3_) δ 161.02, 158.66, 155.22, 134.04, 129.35, 121.53, 42.76, 37.65, 29.80, 28.02, 25.53, 13.01. HRMS (ESI) calcd for C_12_H_14_N_2_OS[M+H]^+^ 235.0900, found 235.0911.
**2-Methyl-6,7,8,9-tetrahydrothieno[3′,2′:4,5]pyrimid[1,2-*a*]azepin-11(5*H*)-one (5m)**. Yield 90%, white solid, mp 114–115 °C; ^1^H NMR (400 MHz, CDCl_3_) δ 6.92–6.87 (m, 1H), 4.39 (t, *J* = 4.7 Hz, 2H), 3.05 (t, *J* = 4.3 Hz, 2H), 2.59 (s, 3H), 1.90–1.75 (m, 6H). ^13^C NMR (100 MHz, CDCl_3_) δ 161.45, 157.91, 156.76, 150.07, 123.08, 120.16, 42.77, 37.63, 29.79, 27.99, 25.39, 16.87. HRMS (ESI) calcd for C_12_H_14_N_2_OS[M+H]^+^ 235.0900, found 235.0909.
**2-Phenyl-6,7,8,9-tetrahydrothieno[3′,2′:4,5]pyrimid[1,2-*a*]azepin-11(5*H*)-one (5n)**. Yield 89%, light yellow solid, mp 134–135 °C; ^1^H NMR (400 MHz, CDCl_3_) δ 7.69 (dt, *J* = 8.0, 1.4 Hz, 2H), 7.51–7.35 (m, 4H), 4.42 (t, *J* = 4.3 Hz, 2H), 3.08 (t, *J* = 4.3 Hz, 2H), 1.91–1.79 (m, 6H). ^13^C NMR (100 MHz, CDCl_3_) δ 161.87, 158.13, 156.88, 152.69, 133.52, 129.60, 129.37, 126.63, 120.82, 120.40, 42.91, 37.71, 29.80, 28.01, 25.40. HRMS (ESI) calcd for C_17_H_16_N_2_OS[M+H]^+^ 297.1056, found 297.1060.
**2-(4-Chlorophenyl)-6,7,8,9-tetrahydrothieno[3′,2′:4,5]pyrimido[1,2-*a*]azepin-11(5*H*)-one (5o)**. Yield 94%, light yellow solid, mp 165–166 °C; ^1^H NMR (400 MHz, CDCl_3_) δ 7.65–7.57 (m, 2H), 7.45–7.36 (m, 3H), 4.42 (t, *J* = 4.7 Hz, 2H), 3.08 (t, *J* = 4.6 Hz, 2H), 1.93–1.80 (m, 6H). ^13^C NMR (100 MHz, CDCl_3_) δ 162.04, 158.05, 156.85, 151.16, 135.59, 132.04, 129.58, 127.81, 121.03, 120.76, 42.95, 37.70, 29.79, 27.99, 25.38. HRMS (ESI) calcd for C_17_H_15_ClN_2_OS[M+H]^+^ 331.0666, found 331.0671.

#### 3.1.3. General Procedure for the Synthesis of Compounds **6a–6o**

a) The solution of 10 mM thieno[3,2-d]pyrimidinones (**5a–5o**) in 100 mL of dried toluene or dioxane was stirred under reflux with 2.0 g (5 mmol) of Lawesson’s reagent. After 4 h (or 6–12 h for dioxane), the reaction mixture was cooled to 20–25 °C, the solvent was removed under reduced pressure, and the residue was subjected to column chromatography using DCM as eluent to yield solid thiones **6a–6o**.

b) One-pot general procedure for the synthesis of compounds **6a–6o**.

A mixture of the appropriate 3-amino-thiophene-2-carboxylate (**3a–3e**, 10 mmol) and the corresponding lactam (12 mmol) was dissolved in 20 mL of DCE, and POCl_3_; (2.3 mL, 25 mmol) was added dropwise under stirring. The reaction mixture was refluxed at 80 °C from 10 min. to 3 h, after which the solvent and excess POCl_3_; were removed under reduced pressure. Without isolation of the intermediate thieno[3,2-*d*]pyrimidinones, the residue was dissolved in 100 mL of anhydrous dioxane, and Lawesson’s reagent (2.0 g, 5 mmol) was added. The mixture was refluxed for 12 h in dioxane under anhydrous conditions, cooled to room temperature, and the solvent was removed under reduced pressure. The crude product was purified by silica gel column chromatography using DCM as the eluent, affording the pure thiones **6a–6o** as solids.

**6,7-Dihydropyrrolo[1,2-*a*]thien[3,2-*d*]pyrimidin-9(5*H*)-thione (6a)**. Yield 93%, white solid, mp 148–150 °C; ^1^H NMR (400 MHz, CDCl_3_) δ 7.83 (d, *J* = 5.4 Hz, 1H), 7.28 (d, *J* = 5.4 Hz, 1H), 4.53 (t, *J* = 7.4 Hz, 2H), 3.33 (t, *J* = 8.0 Hz, 2H), 2.39 (p, *J* = 7.8 Hz, 2H). ^13^C NMR (100 MHz, CDCl_3_) δ 176.79, 160.21, 151.91, 137.31, 136.34, 125.03, 52.48, 32.96, 19.53. HRMS (ESI) calcd for C_9_H_8_N_2_S_2_[M+H]^+^ 209.0202, found 209.0206.
**3-Methyl-6,7-dihydropyrrolo[1,2-*a*]thieno[3,2-*d*]pyrimidin-9(5*H*)-thione (6b)**. Yield 95%, white solid, mp 166–167 °C; ^1^H NMR (400 MHz, CDCl_3_) δ 7.49 (d, *J* = 1.2 Hz, 1H), 4.55 (t, *J* = 7.4 Hz, 2H), 3.35 (t, *J* = 8.0 Hz, 2H), 2.44–2.32 (m, 5H). ^13^C NMR (100 MHz, CDCl_3_) δ 176.70, 159.94, 151.12, 136.53, 133.97, 132.65, 52.36, 33.04, 19.64, 13.22. HRMS (ESI) calcd for C_10_H_10_N_2_S_2_[M+H]^+^ 223.0358, found 223.0363.
**2-Methyl-6,7-dihydropyrrolo[1,2-*a*]thieno[3,2-*d*]pyrimidin-9(5*H*)-thione (6c)**. Yield 99%, white solid, mp 251–253 °C; ^1^H NMR (400 MHz, CDCl_3_) δ 6.96 (q, *J* = 1.1 Hz, 1H), 4.53 (t, *J* = 7.4 Hz, 2H), 3.30 (t, *J* = 8.0 Hz, 2H), 2.60 (d, *J* = 1.1 Hz, 3H), 2.42–2.29 (m, 2H). ^13^C NMR (100 MHz, CDCl_3_) δ 175.42, 160.17, 153.63, 152.57, 135.39, 122.99, 52.46, 33.04, 19.49, 17.39. HRMS (ESI) calcd for C_10_H_10_N_2_S_2_[M+H]^+^ 223.0358, found 223.0361.
**2-Phenyl-6,7-dihydropyrrolo[1,2-*a*]thieno[3,2-*d*]pyrimidin-9(5*H*)-thione (6d)**. Yield 90%, white solid, mp 145–146 °C; ^1^H NMR (400 MHz, CDCl_3_) δ 7.75–7.68 (m, 2H), 7.50–7.37 (m, 4H), 4.54 (t, *J* = 7.4 Hz, 2H), 3.32 (t, *J* = 8.0 Hz, 2H), 2.44–2.31 (m, 2H).^13^C NMR (100 MHz, CDCl_3_) δ 175.81, 160.52, 155.36, 152.61, 135.74, 133.27, 130.03, 129.44, 126.61, 120.07, 52.52, 33.08, 19.48.HRMS (ESI) calcd for C_15_H_12_N_2_S_2_[M+H]^+^ 285.0515, found 285.0519
**2-(4-Chlorophenyl)-6,7-dihydropyrrolo[1,2-*a*]thieno[3,2-*d*]pyrimidin-9(5*H*)-thione (6e)**. Yield 89%, white solid, mp 161–162 °C; ^1^H NMR (400 MHz, CDCl_3_) δ 7.65 (d, *J* = 8.2 Hz, 2H), 7.48–7.37 (m, 3H), 4.54 (t, *J* = 7.4 Hz, 2H), 3.33 (t, *J* = 8.0 Hz, 2H), 2.39 (p, *J* = 7.8 Hz, 2H). ^13^C NMR (100 MHz, CDC l_3_) δ 175.94, 160.67, 153.80, 152.55, 136.06, 135.98, 131.82, 129.70, 127.81, 120.46, 52.54, 33.10, 19.50. HRMS (ESI) calcd for C_15_H_11_ClN_2_S_2_[M+H]^+^ 319.0125, found 319.0132.
**7,8-Dihydro-5*H*-pyrido[1,2-*a*]thieno[3,2-*d*]pyrimidin-10(6*H*)-thione (6f)**. Yield 90%, white solid, mp 131–132 °C; ^1^H NMR (400 MHz, CDCl_3_) δ 7.83 (d, *J* = 5.4 Hz, 1H), 7.27 (d, *J* = 5.4 Hz, 1H), 4.62 (t, *J* = 6.2 Hz, 2H), 3.11 (t, *J* = 6.8 Hz, 2H), 2.15–1.94 (m, 4H). ^13^C NMR (100 MHz, CDCl_3_) δ 179.62, 156.42, 149.56, 138.21, 137.38, 124.94, 49.16, 32.67, 22.65, 19.17. HRMS (ESI) calcd for C_10_H_10_N_2_S_2_[M+H]^+^ 223.0358, found 223.0367.
**3-Methyl-7,8-dihydro-5*H*-pyrido[1,2-*a*]thieno[3,2-*d*]pyrimidin-10(6*H*)-thione (6g)**. Yield 94%, white solid, mp 163–164 °C; ^1^H NMR (400 MHz, CDCl_3_) δ 7.48 (q, *J* = 1.1 Hz, 1H), 4.65 (t, *J* = 6.2 Hz, 2H), 3.13 (t, *J* = 6.8 Hz, 2H), 2.38 (d, *J* = 1.1 Hz, 3H), 2.13–2.04 (m, 2H), 2.04–1.95 (m, 2H). ^13^C NMR (100 MHz, CDCl_3_) δ 179.44, 156.15, 148.88, 137.47, 133.95, 133.35, 48.88, 32.73, 22.62, 19.19, 13.07. HRMS (ESI) calcd for C_11_H_12_N_2_S_2_[M+H]^+^ 237.0515, found 237.0521.
**2-Methyl-7,8-dihydro-5*H*-pyrido[1,2-*a*]thieno[3,2-*d*]pyrimidin-10(6*H*)-thione (6h)**. Yield 91%, white solid, mp 240–241 °C; ^1^H NMR (400 MHz, CDCl_3_) δ 6.93 (s, 1H), 4.60 (t, *J* = 6.2 Hz, 2H), 3.08 (t, *J* = 6.8 Hz, 2H), 2.60 (d, *J* = 0.9 Hz, 3H), 2.08 (p, *J* = 6.4 Hz, 2H), 1.98 (p, *J* = 6.6 Hz, 2H). ^13^C NMR (100 MHz, CDCl_3_) δ 178.09, 156.49, 154.46, 150.36, 136.54, 122.77, 49.02, 32.63, 22.65, 19.19, 17.33.
**2-Phenyl-7,8-dihydro-5*H*-pyrido[1,2-*a*]thieno[3,2-*d*]pyrimidin-10(6*H*)-thione (6i)**. Yield 93%, white solid, mp 195–196 °C; ^1^H NMR (400 MHz, CDCl_3_) δ 7.77–7.69 (m, 2H), 7.50–7.38 (m, 4H), 4.62 (t, *J* = 6.2 Hz, 2H), 3.11 (t, *J* = 6.8 Hz, 2H), 2.10 (p, *J* = 6.0 Hz, 2H), 2.01 (p, *J* = 6.7 Hz, 2H). ^13^C NMR (100 MHz, CDCl_3_) δ 178.47, 156.82, 156.14, 150.32, 136.83, 133.32, 130.04, 129.44, 126.65, 119.87, 49.11, 32.68, 22.67, 19.18. HRMS (ESI) calcd for C_16_H_14_N_2_S_2_[M+H]^+^ 299.0671, found 299.0675.
**2-(4-Chlorophenyl)-7,8-dihydro-5*H*-pyrido[1,2-*a*]thieno[3,2-*d*]pyrimidin-10(6*H*)-thione (6j)**. Yield 89%, white solid, mp 216–217 °C; ^1^H NMR (400 MHz, cdcl_3_) δ 7.69–7.61 (m, 2H), 7.47–7.39 (m, 3H), 4.61 (t, *J* = 6.2 Hz, 2H), 3.11 (t, *J* = 6.8 Hz, 2H), 2.16–2.06 (m, 2H), 2.05–1.95 (m, 2H). ^13^C NMR (100 MHz, CDCl_3_) δ 178.58, 156.98, 154.55, 150.21, 137.02, 136.07, 131.85, 129.69, 127.83, 120.25, 49.18, 32.69, 22.67, 19.18. HRMS (ESI) calcd for C_16_H_13_ClN_2_S_2_[M+H]^+^ 333.0281, found 333.0285.
**6,7,8,9-Tetrahydrothieno[3′,2′:4,5]pyrimido[1,2-*a*]azepin-11(5*H*)-thione (6k)**. Yield 93%, white solid, mp 118–119 °C; ^1^H NMR (400 MHz, CDCl_3_) δ 7.84 (d, *J* = 5.4 Hz, 1H), 7.26 (s, 1H), 5.08 (t, *J* = 5.0 Hz, 2H), 3.23 (t, *J* = 5.2 Hz, 2H), 1.94–1.87 (m, 6H). ^13^C NMR (100 MHz, CDCl_3_) δ 179.86, 160.83, 149.49, 138.78, 137.57, 125.25, 50.29, 38.14, 29.36, 26.63, 25.30. HRMS (ESI) calcd for C_11_H_12_N_2_S_2_[M+H]^+^ 237.0515, found 237.0519.
**3-Methyl-6,7,8,9-tetrahydrothieno[3′,2′:4,5]pyrimido[1,2-*a*]azepin-11(5*H*)-thione (6l)**. Yield 94%, white solid, mp 137–138 °C; ^1^H NMR (400 MHz, CDCl_3_) δ 7.49 (d, *J* = 1.1 Hz, 1H), 5.09 (t, *J* = 5.4 Hz, 2H), 3.24 (t, *J* = 6.2 Hz, 2H), 2.37 (d, *J* = 1.1 Hz, 3H), 1.92–1.88 (m, 6H). ^13^C NMR (100 MHz, CDCl_3_) δ 179.77, 160.47, 148.84, 137.67, 134.34, 133.88, 50.13, 38.15, 29.41, 26.69, 25.45, 13.03. HRMS (ESI) calcd for C_12_H_14_N_2_S_2_[M+H]^+^ 251.0671, found 251.06776.
**2-Methyl-6,7,8,9-tetrahydrothieno[3′,2′:4,5]pyrimido[1,2-*a*]azepin-11(5*H*)-thione (6m)**. Yield 88%, white solid, mp 255–256 °C; ^1^H NMR (400 MHz, CDCl_3_) δ 6.93 (q, *J* = 1.0 Hz, 1H), 5.06 (t, *J* = 5.8 Hz, 2H), 3.19 (t, *J* = 4.6 Hz, 2H), 2.59 (d, *J* = 1.2 Hz, 3H), 1.91–1.85 (m, 6H). ^13^C NMR (100 MHz, CDCl_3_) δ 178.32, 160.90, 155.05, 150.28, 136.68, 123.08, 50.17, 38.12, 29.36, 26.65, 25.27, 17.32. HRMS (ESI) calcd for C_12_H_14_N_2_S_2_[M+H]^+^ 251.0671, found 251.0675.
**2-Phenyl-6,7,8,9-tetrahydrothieno[3′,2′:4,5]pyrimido[1,2-*a*]azepin-11(5*H*)-thione (6n)**. Yield 89%, white solid, mp 202–203 °C; ^1^H NMR (400 MHz, CDCl_3_) δ 7.76–7.68 (m, 2H), 7.50–7.38 (m, 4H), 5.07 (t, *J* = 5.5 Hz, 2H), 3.22 (t, *J* = 4.7 Hz, 2H), 1.93–1.88 (m, 6H). ^13^C NMR (100 MHz, CDCl_3_) δ 178.75, 161.24, 156.72, 150.27, 137.03, 133.32, 130.05, 129.46, 126.64, 120.20, 50.23, 38.16, 29.37, 26.69, 25.30. HRMS (ESI) calcd for C_17_H_16_N_2_S_2_[M+H]^+^ 313.0828, found 313.0833.
**2-(4-Chlorophenyl)-6,7,8,9-tetrahydrothieno[3′,2′:4,5]pyrimido[1,2-*a*]azepin-11(5*H*)-thione (6o)**. Yield 87%, white solid, mp 230–231 °C; ^1^H NMR (400 MHz, cdcl_3_) δ 7.68–7.60 (m, 2H), 7.46–7.38 (m, 3H), 5.06 (t, *J* = 5.8 Hz, 2H), 3.21 (t, *J* = 6.0 Hz, 2H), 1.93–1.88 (m, 6H). ^13^C NMR (100 MHz, CDCl_3_) δ 178.83, 161.39, 155.10, 150.15, 137.21, 136.07, 131.82, 129.69, 127.80, 120.56, 50.26, 38.15, 29.36, 26.67, 25.29. HRMS (ESI) calcd for C_17_H_15_ClN_2_S_2_[M+H]^+^ 347.0438, found 347.0443.

### 3.2. Biology

#### 3.2.1. Cell Lines and Culture Conditions

The human HeLa cervical cancer and the human HT-29colon cancer cell lines were obtained from the Shanghai Cell Bank of the Chinese Academy of Sciences (Shanghai, China). The cells were cultured in DMEM (Dulbecco’s Modified Eagle Medium) supplemented with 10% FBS (fetal bovine serum), 100 U/mL penicillin, and 100 μg/mL streptomycin at 37 °C in a humidified atmosphere with 5% CO_2_.

#### 3.2.2. MTT Assay

The antiproliferative effects of compounds **5a-o** and **6a-o** towards HeLa and HT-29 cells were assessed using an MTT assay. Briefly, cells were seeded in 96-well plates at a density of 5 × 10^3^ cells per well. The cells were seeded in 96 well plates (Corning Inc., Corning, NY, USA) at a density of 1 ×10^4^ –4 × 10^4^ cells/well at 37◦C for 24 h. After overnight incubation, the cells were treated with different concentrations of the compound, using DMSO as a vehicle control, and then incubated for an additional 72 h. Doxorubicin (DOX) was included as a positive control at equivalent concentrations. Following incubation, 20 μL of MTT solution (5 mg/mL in PBS) was added to each well. After incubating for 4 h at 37 °C, the medium was removed and 150 μL of DMSO was added to dissolve the formazan crystals. Absorbance was measured at 570 nm using a microplate reader, and the inhibition rate was calculated [91].

#### 3.2.3. Effect of Compound **6e** on Morphological Changes in HeLa Cells

HeLa cells were cultured and treated with varying concentrations of compound **6e** (0.1, 0.3, 1.0, and 3.0 μM) for 24 h. Untreated cells served as a control. After treatment, the cells were harvested and washed with phosphate-buffered saline (PBS). Morphological changes were assessed using an inverted fluorescence microscope. Images were captured to evaluate cell structure and nuclear morphology.

#### 3.2.4. Colony Formation Assay

A colony formation assay was conducted to evaluate the inhibitory effects of compound 6e on the anchorage-independent growth of HeLa cells. HeLa cells were treated with **6e** at 0, 0.1, 0.3, 1.0, and 3.0 μM concentrations for two weeks. The cells were then seeded in soft agar to facilitate colony formation. Control cells were allowed to grow without treatment. Colony formation was assessed by counting visible colonies and measuring their area after incubation. Colony formation rates were calculated as a percentage relative to the control group [36,92].

#### 3.2.5. Wound Healing Assay

HeLa cells were seeded in a 6-well cell culture plate and incubated overnight until they reached 90–95% confluence. A wound (scratch) was created using a pipette tip in each well. Images were captured using a microscope to document the initial wound width. After creating the scratch, the cells were treated with compound **6e** at 0, 0.1, 0.3, 1.0, and 3.0 μM concentrations and incubated for 0, 12, or 24 h at 37 °C in a 5% CO_2_ atmosphere. The scratches were imaged again at the specified time points. Quantitative analysis of wound healing was performed by measuring the width of the remaining wound area and calculating the wound healing rate [93].

#### 3.2.6. Transwell Migration Assay

The Transwell migration assay was conducted to evaluate the impact of compound **6e** on the migratory capacity of HeLa cells. Briefly, HeLa cells were plated in 24-well inserts and treated with compound **6e** for 24 h. After treatment, the cells were fixed, stained, and photographed. After staining, 100 μL of 10% acetic acid was added to each well to extract the dye for 10 min. Then, the solution was transferred to a 96-well plate, and the optical density (OD) at 600 nm was measured using a microplate reader [94].

#### 3.2.7. Cell Cycle Analysis by Flow Cytometry

To investigate the effect of compound **6e** on the distribution of cell cycle phases in HeLa cells, flow cytometry was used. HeLa cells were treated with varying concentrations of compound 6e (0.0, 0.3, 1.0, and 3.0 μM) and incubated for 24 h. The cells were harvested, washed, and fixed in 70% ethanol after treatment. Propidium iodide (PI) staining was then performed to analyze DNA content. The cell cycle distribution was assessed using a flow cytometer, and the data were analyzed to determine the percentage of cells in each cell cycle phase (G0/G1, S, and G2/M).

#### 3.2.8. Effect of Compound **6e** on Microtubule Network

An immunofluorescence assay was conducted to evaluate the impact of compound **6e** on microtubule networks in HeLa cells. HeLa cells were treated with **6e** at a concentration of 1.0 μM for 12 h and then compared to a colchicine control (0.05 μM). After treatment, the cells were fixed and stained for DNA (blue) and α-tubulin (green). Images were captured using a fluorescence microscope to evaluate the organization and arrangement of microtubule structures [95].

#### 3.2.9. Analysis of Reactive Oxygen Species (ROS) Levels

Flow cytometry was utilized alongside the fluorescent probe DCFH-DA to evaluate the levels of ROS in HeLa cells following treatment with compound **6e**. The cells were treated with various concentrations of compound **6e** (0.0, 0.3, 1.0, and 3.0 μM) for 24 h. After treatment, the cells were incubated with DCFH-DA to detect ROS. The resulting fluorescence intensity from DCFH-DA oxidation was measured via flow cytometry. Higher fluorescence indicates increased ROS levels [36].

#### 3.2.10. Apoptosis Analysis by Flow Cytometry

To investigate the apoptotic effects of compound **6e** on HeLa cells, a flow cytometry analysis was performed using dual staining with annexin V-FITC and PI. The HeLa cells were treated with **6e** at concentrations of 0.0, 0.3, 1.0, and 3.0 μM for 24 h. Control cells were maintained in medium without treatment. After treatment, the cells were stained with Annexin V-FITC to identify phosphatidylserine exposure and with PI to assess membrane integrity. Flow cytometry was then used to categorize the cells into four populations: unaffected (annexin V−/PI−), early apoptotic (annexin V+/PI−), late apoptotic (annexin V+/PI+), and necrosis (annexin V−/PI+) [36].

### 3.3. Molecular Docking Study of Compound **6e**

#### 3.3.1. Preparation of Target Protein Structure

All protein crystal structures were downloaded from the Protein Data Bank (https://www.rcsb.org/, accessed on 28 February 2025) [36,96]. The protein structures were processed using the DS2019.1 platform [36]. First, the proteins were imported into DS2019.1, and water molecules, solvent molecules, ions, and other irrelevant components were removed. The protein was initially processed using the Macromolecule > Prepare Protein > Clean Protein module, followed by further refinement with the Macromolecule > Prepare Protein > Automatic Preparation module (to ensure the structural and usability of the protein), using the CHARMm force field. The original ligand of the protein was selected as the docking site, and a 15 Å spherical docking box was created.

#### 3.3.2. Molecular Docking Analysis

The molecular docking process and optimization were carried out using the CDOCKER module within the DS2019.1 platform [36,97]. Initially, the function “Receptor-Ligand Interactions > Docking Optimization > Dock Ligands” was used to dock with the reference ligand, verifying the validity of the chosen docking method. This methodology was subsequently applied to screen the dataset.

#### 3.3.3. Screening and Analysis of Docking Results

The interaction modes between the compounds and proteins were analyzed to determine binding patterns with target residues, including hydrogen bonding, π-π interactions, and hydrophobic interactions. Combined with the docking scores of the compounds, we inferred whether the screened compounds exhibited potential biological activity.

#### 3.3.4. Molecular Dynamics (MD) Simulation

MD simulations of protein-ligand complexes were performed using Desmond 2020 software [36]. The OPLS3e force field was employed for the simulations, and the TIP3P water model was used to solvate the system. Charge neutralization was achieved by adding ions where necessary. A global energy minimization of the system was conducted using the OPLS3e all-atom force field. The geometric structures of water molecules, along with the bond lengths and angles of heavy atoms, were constrained using the SHAKE algorithm. Periodic boundary conditions (PBC) were applied to simulate a continuous system, and the particle-mesh Ewald (PME) method was utilized to maintain long-range electrostatic interactions. The system was equilibrated under an NPT ensemble at 300 K and 1.0 bar, with temperature and pressure coupling managed by the Berendsen algorithm. In the subsequent production run, the system was simulated for 200 ns with a 1.2 fs time step, and trajectories were recorded every 50 ps, resulting in a total of 20,000 frames. The root-mean-square deviation (RMSD) of the backbone atoms was calculated and analyzed graphically to characterize the dynamics of protein-ligand interactions.

## 4. Conclusions

In summary, the one-pot synthesis of tricyclic thieno[3,2-*d*]pyrimidinones relied on a condensation reaction involving cyclic lactams and thieno[3,2-*d*]pyrimidin-thiones, which was facilitated by Lawesson’s reagent. The study employed a scaffold-hopping strategy. The anticancer properties were evaluated against two human cancer cell lines (HeLa and HT-29). This study focused on HeLa and HCT-116 cells because of their relevance to CDK-associated pathways. Additional bioassays were conducted on HeLa cells using the most active compound, **6e**. This compound significantly inhibited HeLa cell colony formation in a dose-dependent manner, suggesting its ability to hinder the colony formation of cancer cells. Furthermore, **6e** inhibited microtubule assembly and disrupted the cytoskeleton, similarly to colchicine. The data confirm that **6e** selectively targeted the G2/M phase of the cell cycle, as it is typical of tubulin assembly inhibitors. Molecular docking analysis revealed that **6e** frequently bound to CDKs. Although direct biochemical validation (e.g., kinase inhibition assays) was not performed, the observed dose-dependent G2/M cell cycle arrest and favorable binding interactions with CDKs in molecular simulations support the plausibility of CDK-mediated activity. These correlations provide a useful framework for ongoing SAR optimization and mechanistic studies. In addition, to validate and generalize the observed activity patterns, it will be essential to evaluate a broader panel of cancer cell lines. These results imply that further structural modifications and detailed exploration of **6e**’s mechanism of action could lead to the development of promising anticancer agents based on these scaffolds.

## Figures and Tables

**Table 1:** Optimization of the conditions for the annulation of **3a**.

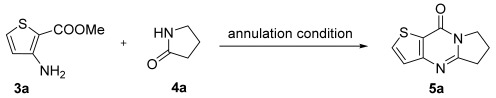				
Entry	Substrate	Cyclization conditions	Product	Yield (%)
1	**3a**	POCl_3_, DCM, 45 °C, 5 h	**5a**	42
2	**3a**	POCl_3_, DCE, 80 °C, 2 h	**5a**	79
3	**3a**	POCl_3_, Dioxane, 100 °C, 8 h	**5a**	77
4	**3a**	POCl_3_, Toluene, 120 °C, 20 h	**5a**	28
5	**3a**	POCl_3_, solvent-free, 100 °C, 3 h	**5a**	68
6	**3a**	POCl_3_, solvent-free, 140 °C, 2 h	**5a**	81

**Table 2:** Molecular Docking Results for Compound **6e** with Targeted Kinases.

Compound	Structure	Target	Pdbid	Cdocker Energy (kcal/mol)	Combination Type
**6e**	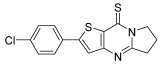	CDK2	2C5Y	−9.4959	Hydrogen bond, Hydrophobic interaction, Electrostatic interaction
		CDK3	3GC0	0.114814	Hydrogen bond, Hydrophobic interaction, Electrostatic interaction, π-interaction
		CDK5-p25	1UNG	−5.79453	Hydrogen bond, Hydrophobic interaction, Electrostatic interaction, π-interaction
		CDK5-p35	1UNH	−8.30506	Hydrogen bond, Hydrophobic interaction, Electrostatic interaction, π-interaction
		CK1y1	6F1W	−7.23817	Hydrogen bond, Hydrophobic interaction, Vander Waals forces, Electrostatic interaction
		CSK	1BYG	−2.28896	Hydrogen bond, Hydrophobic interaction, Electrostatic interaction, π-interaction

## Data Availability

Data are included in the article and Supplementary Materials.

## Supplementary Materials

The following supporting information can be downloaded at: https://www.mdpi.com/article/10.3390/ijms26178528/s1.
