# Supplementary material for: Scaffold-Hopping Design and Synthesis of Thieno[3,2-d]pyrimidines: Anticancer Activity, Apoptosis Induction, and In Silico Inhibition of CDKs
Source: Int J Mol Sci. 2025 Sep 2;26(17):8528. doi: 10.3390/ijms26178528 (PMC12429047; doi:10.3390/ijms26178528)
Supplement: Supplementary file 1 [file ijms-26-08528-s001.zip › ijms-3787083-supplementary.pdf]

Supplementary materials

^{1}H and ^{13}C NMR spectrum of compounds 5a-o and 6a-o

Table S1 and Figures S1-S60

Table S1. IC_{50} values for human embryonic kidney (HEK-293) cells and SI value of **6e** against the HeLa cell line (after 72 h.).

Compound	IC_{50} (±SD, μM)	IC_{50} (HeLa), μM	SI^{HeLa}
**6e**	148 ± 0.16	0.591 ± 0.23	250.4

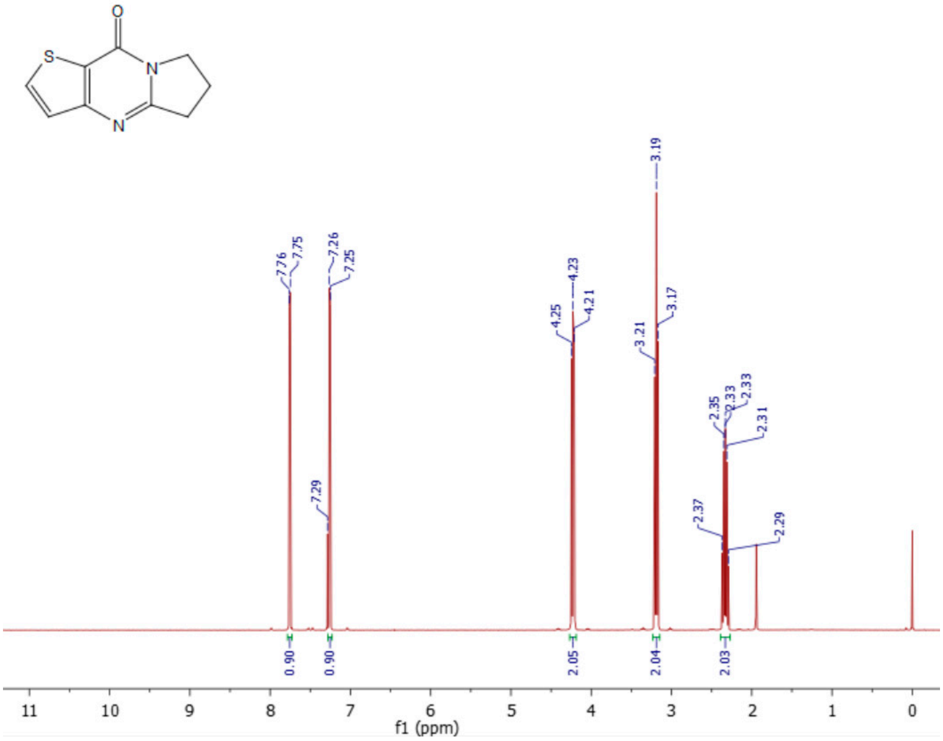

Figure S1: ^{1}H NMR spectrum of compound **5ª**

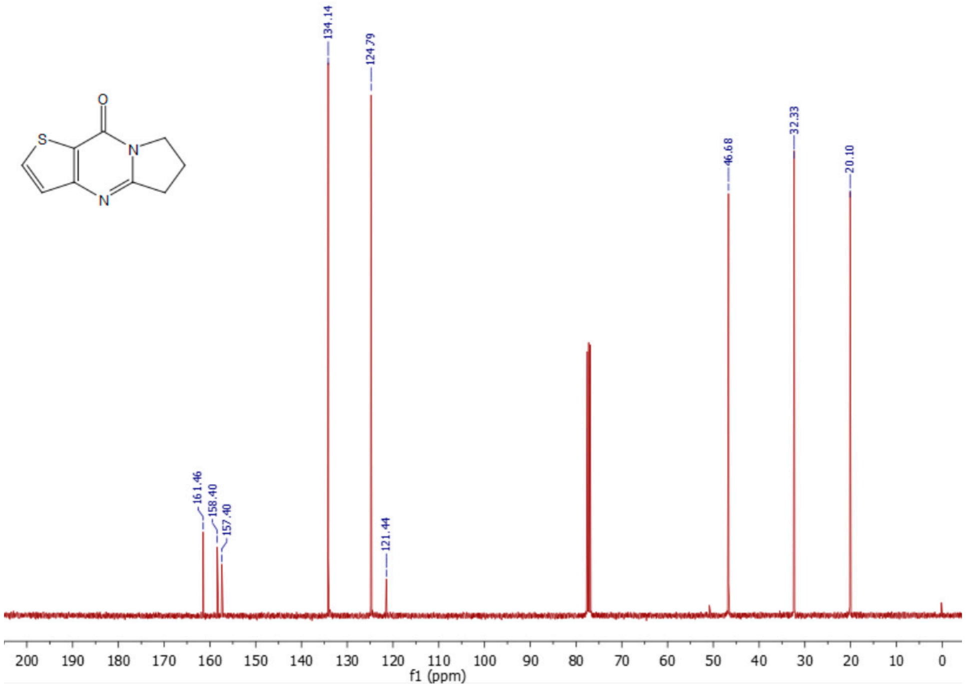

Figure S2: ^{13}C NMR spectrum of compound **5a**

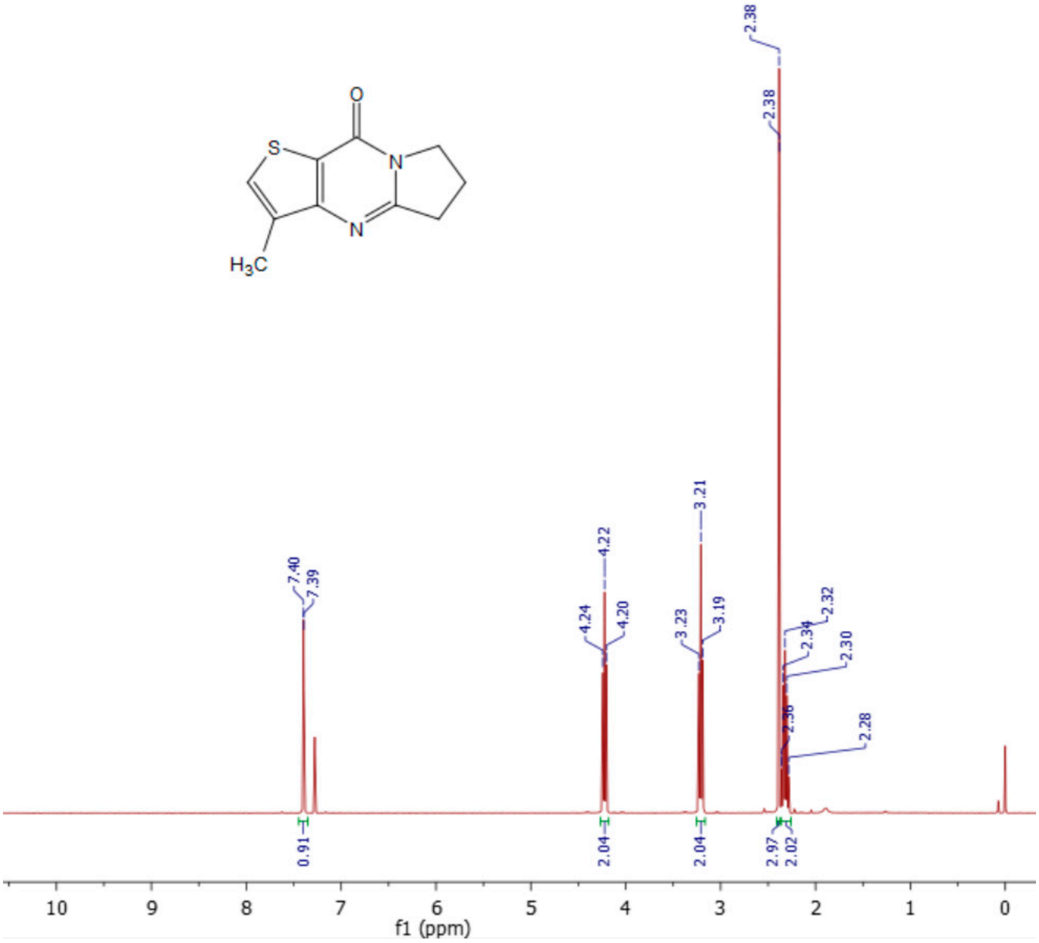

Figure S3: ^{1}H NMR spectrum of compound **5b**

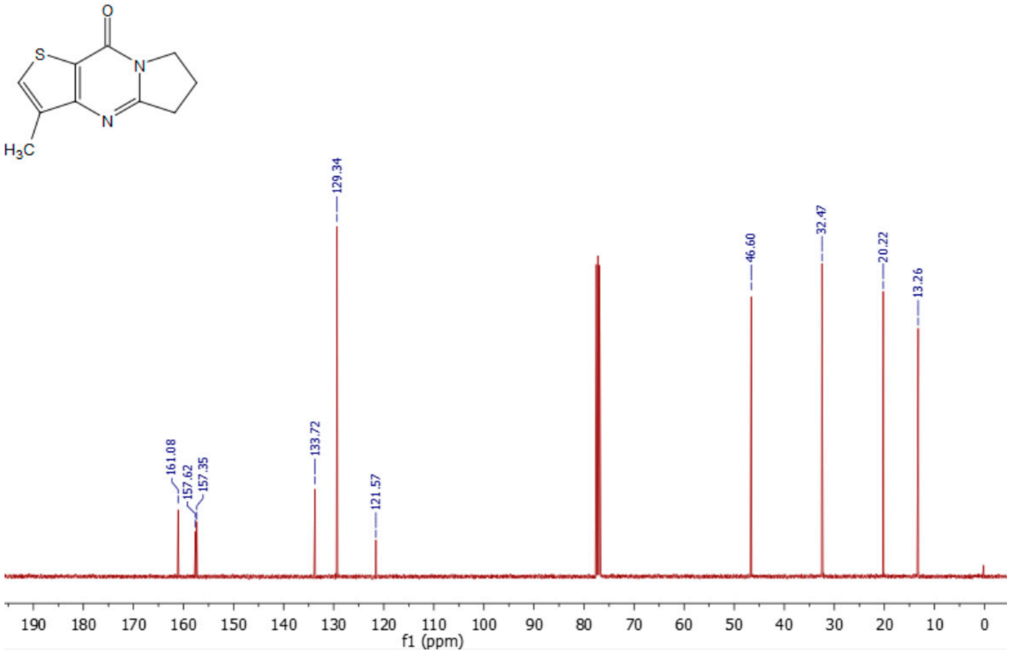

Figure S4: ^{13}C NMR spectrum of compound **5b**

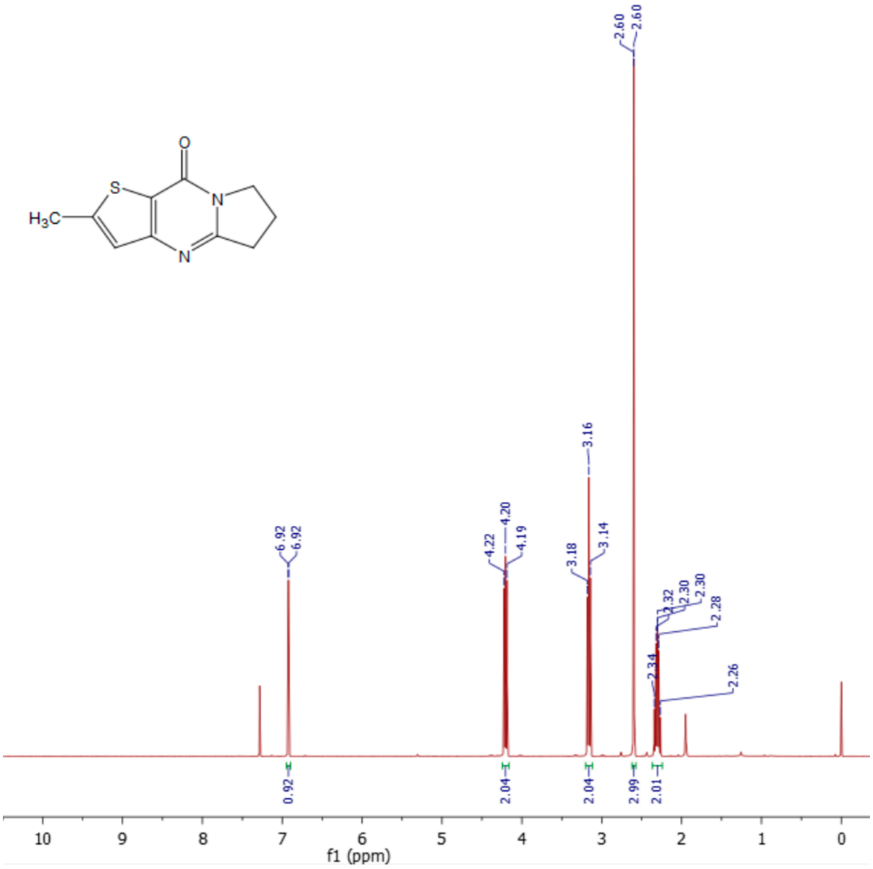

Figure S5: ^{1}H NMR spectrum of compound **5c**

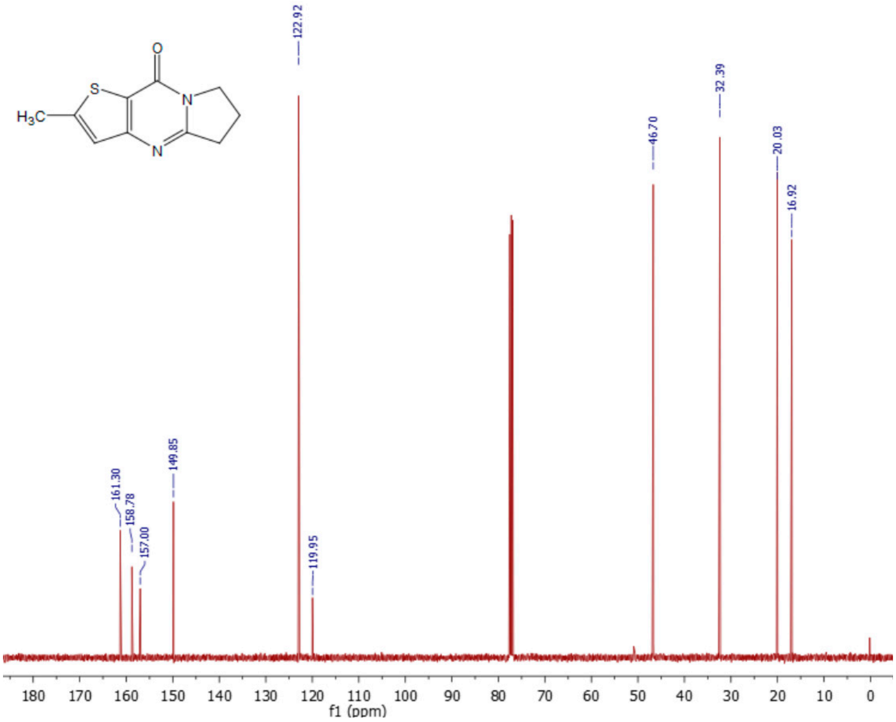

Figure S6: ^{13}C NMR spectrum of compound **5c**

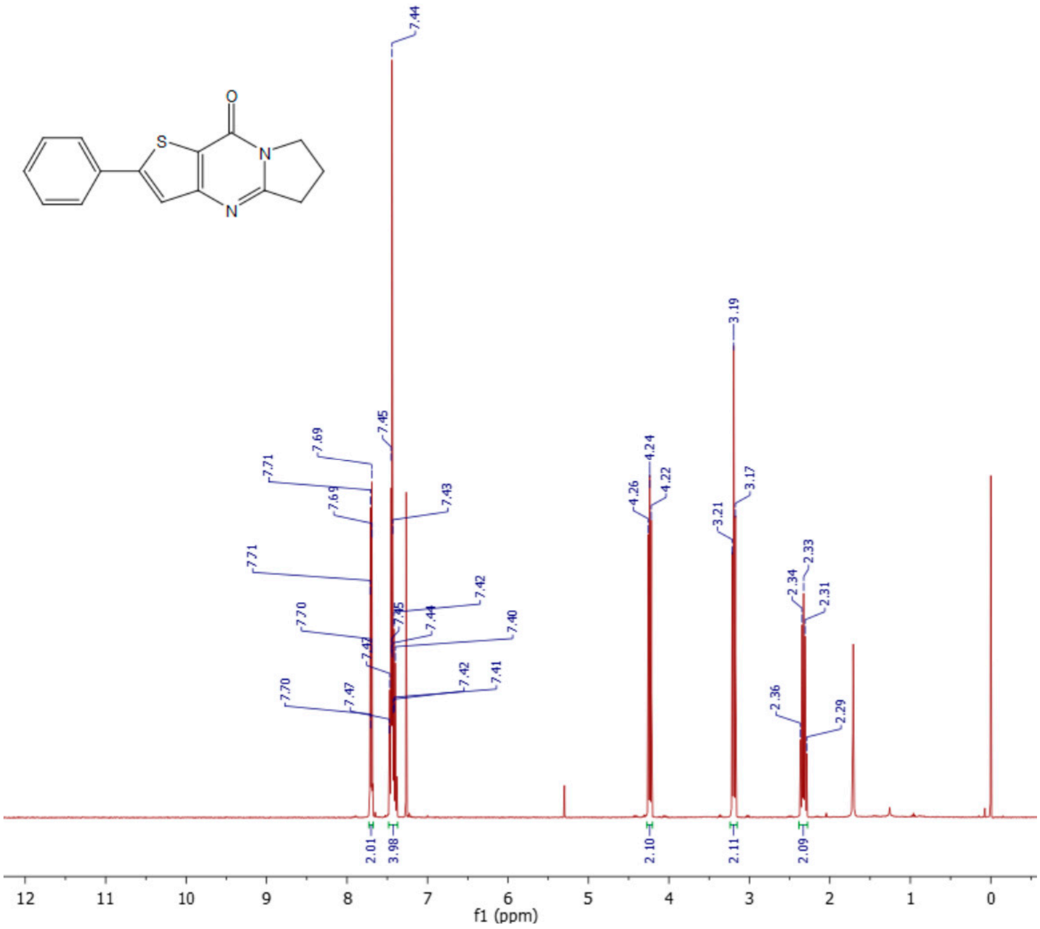

Figure S7: ^{1}H NMR spectrum of compound **5d**

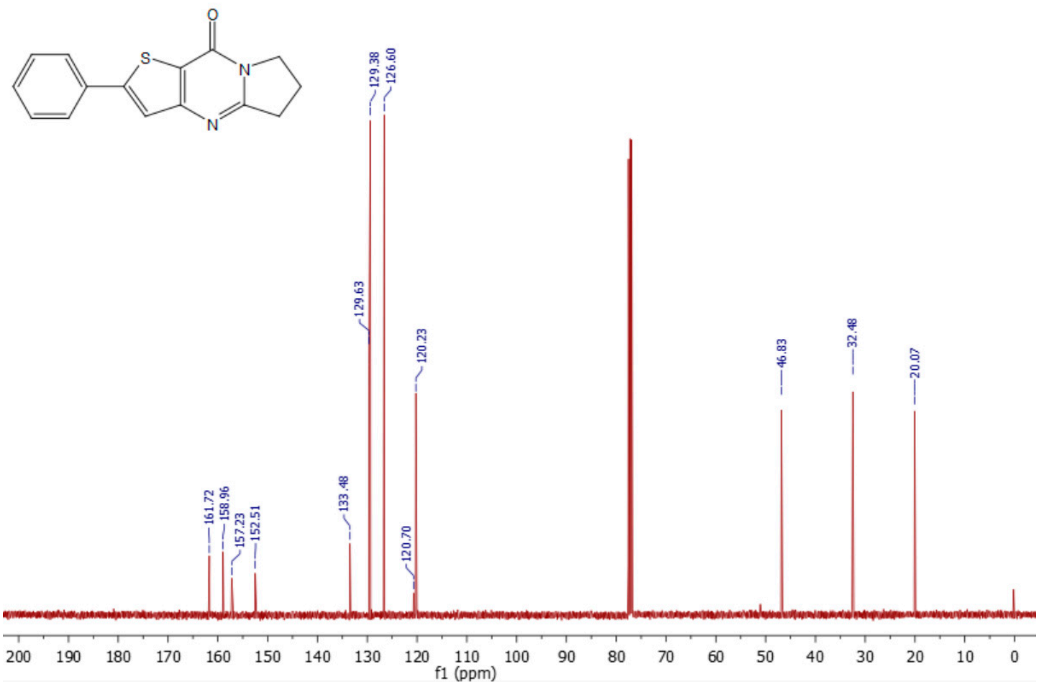

Figure S8: ^{13}C NMR spectrum of compound **5d**

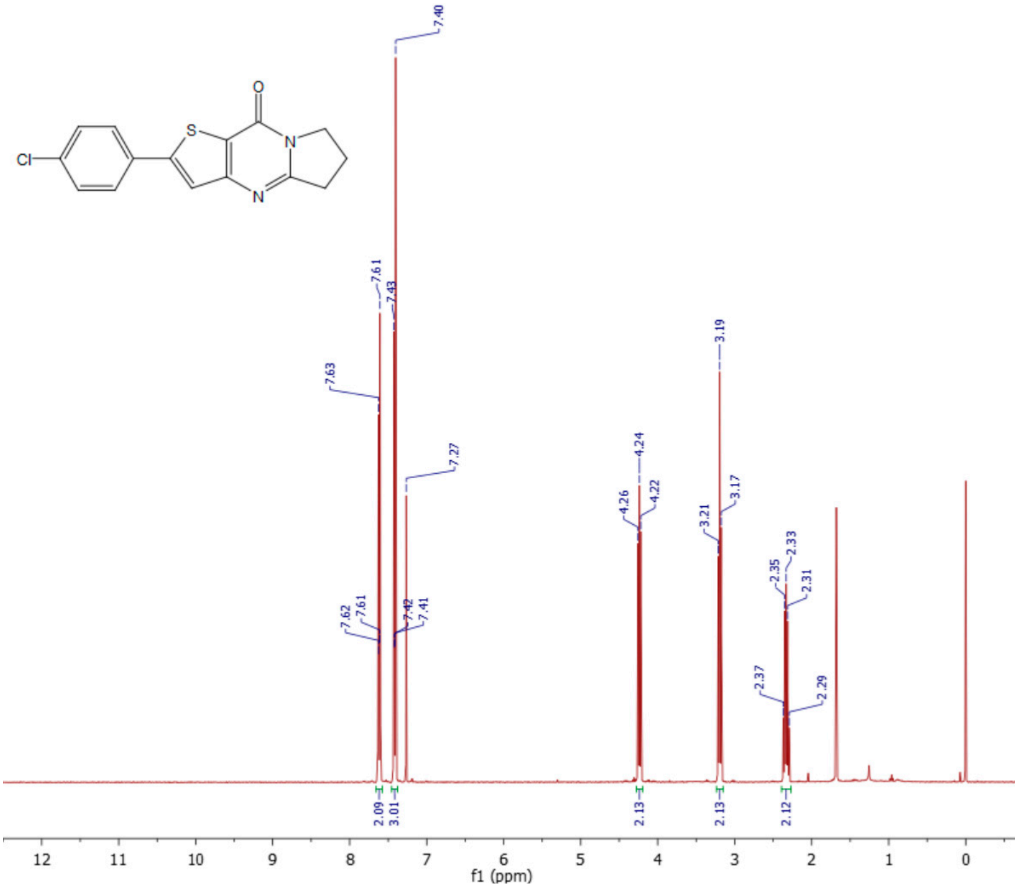

Figure S9: ^{1}H NMR spectrum of compound **5e**

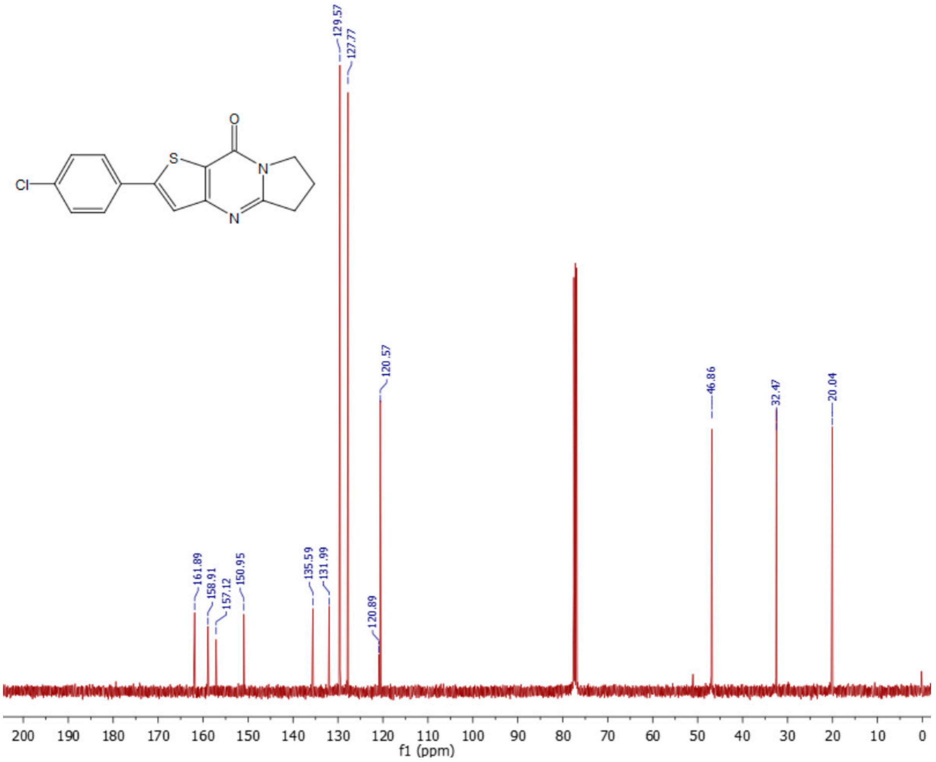

Figure S10: ^{13}C NMR spectrum of compound **5e**

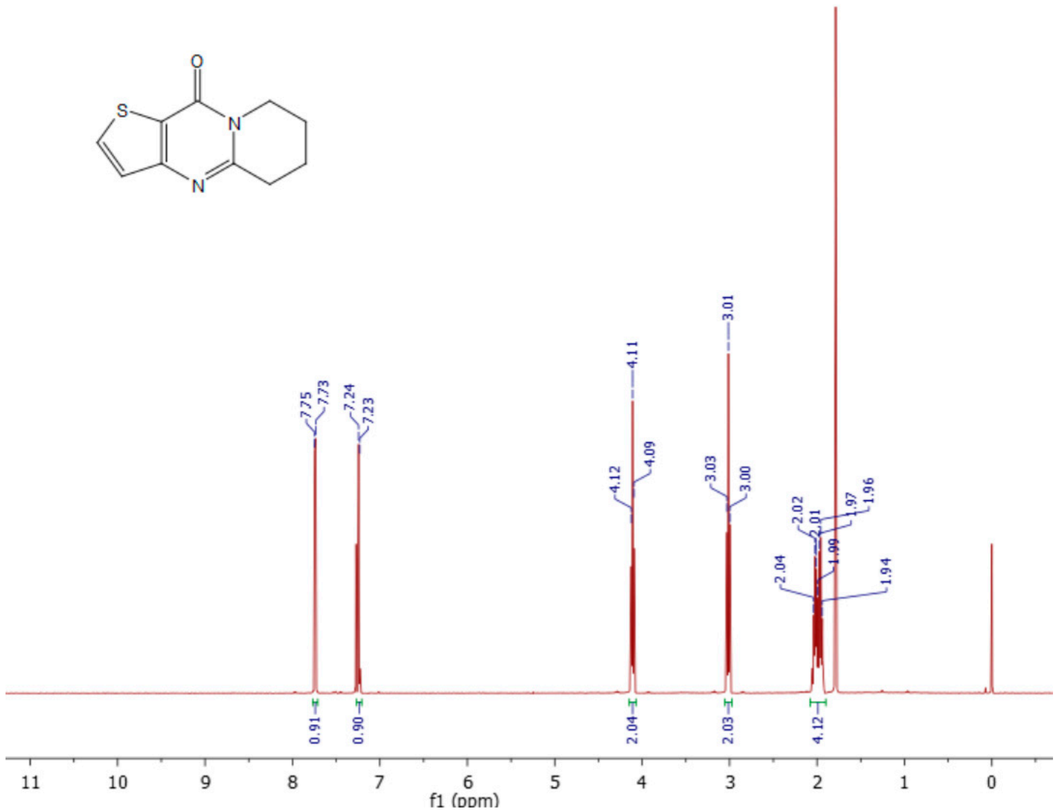

Figure S11: ^{1}H NMR spectrum of compound **5f**

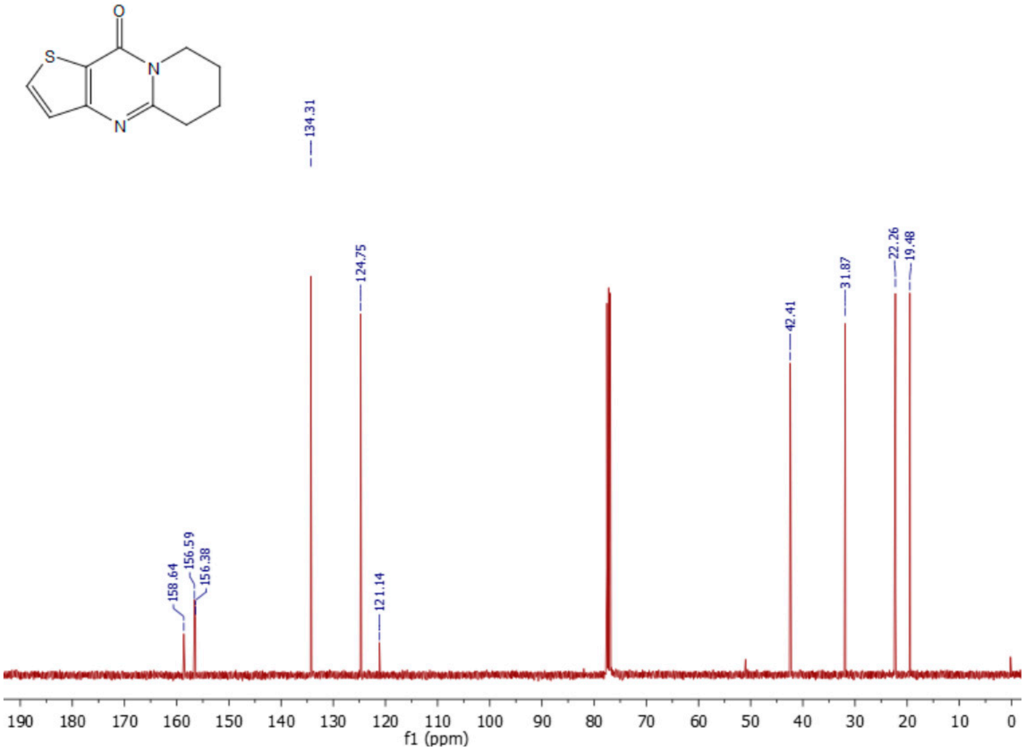

Figure S12: ^{13}C NMR spectrum of compound **5f**

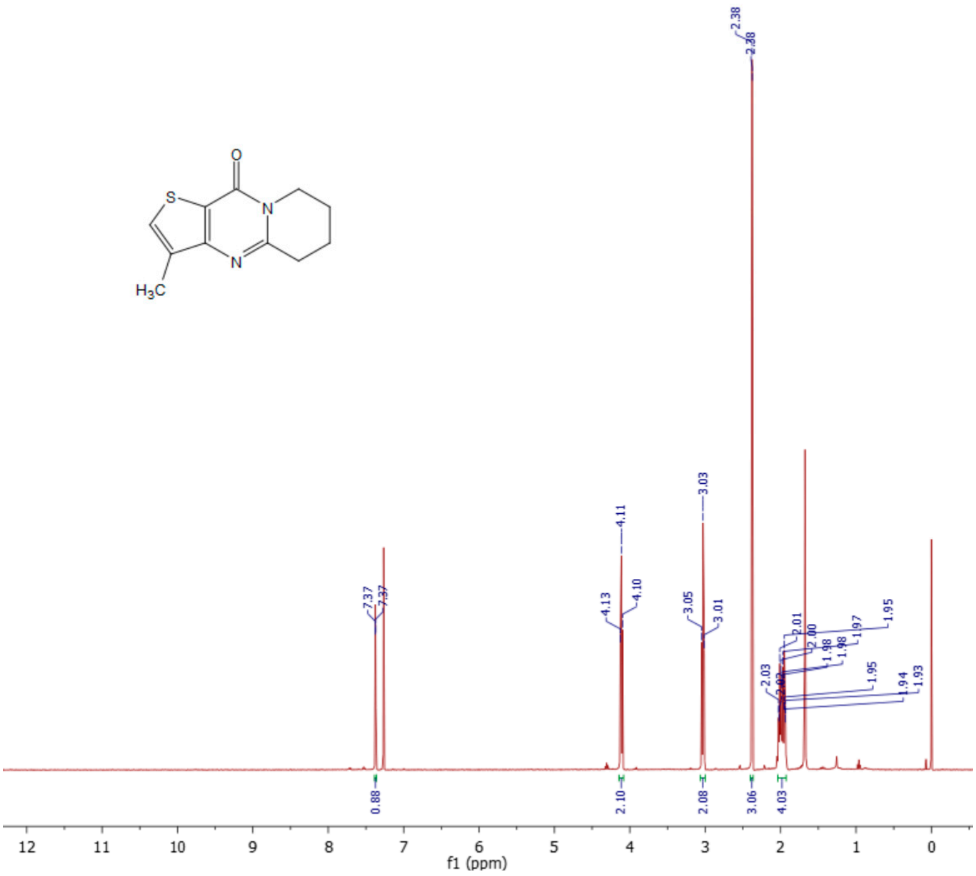

Figure S13: ^{1}H NMR spectrum of compound **5g**

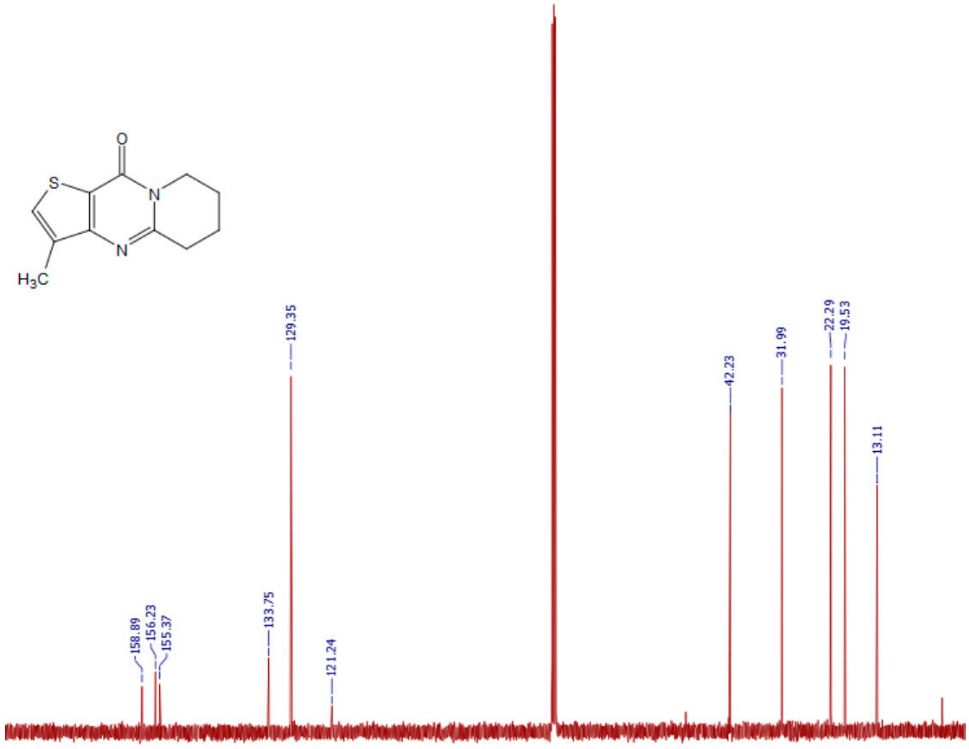

Figure S14: ^{13}C NMR spectrum of compound **5g**

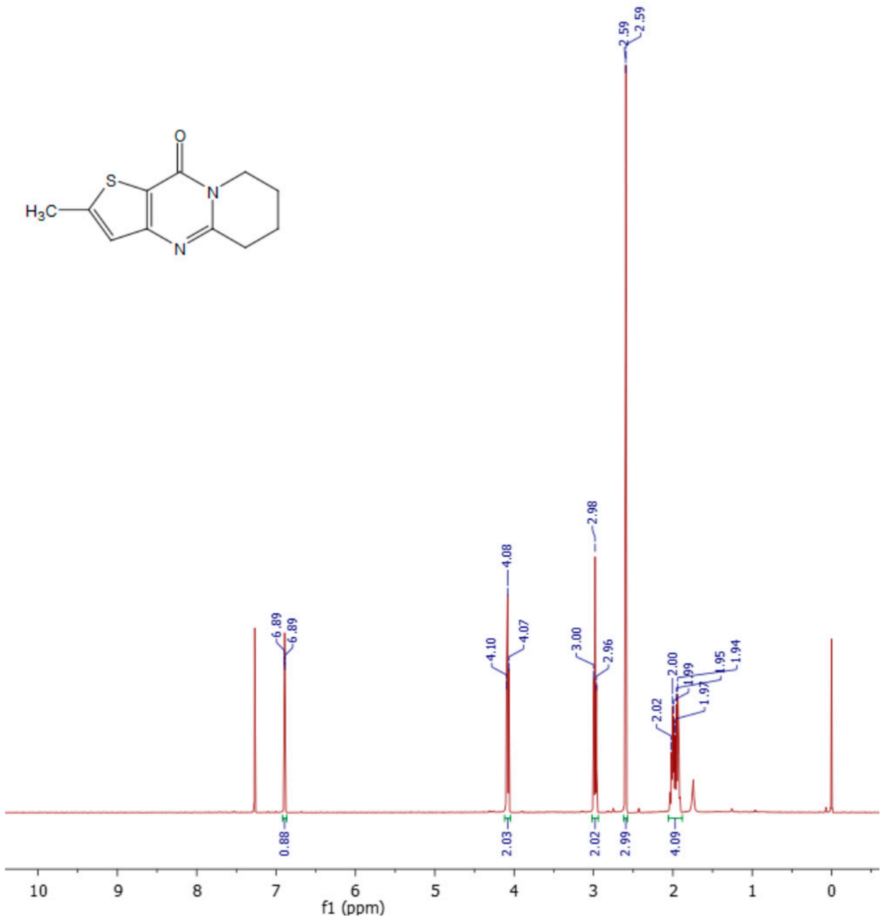

Figure S15: ^{1}H NMR spectrum of compound **5h**

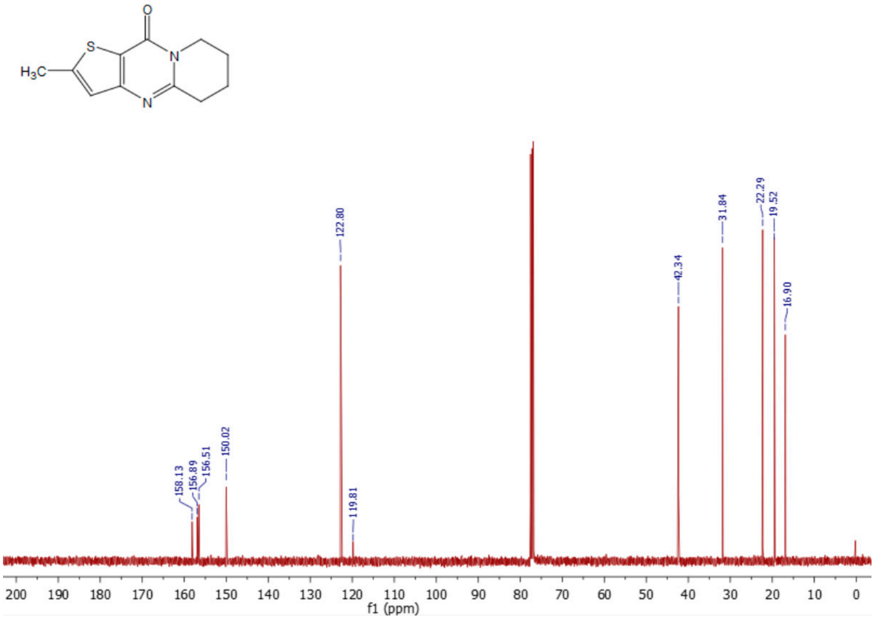

Figure S16: ^{13}C NMR spectrum of compound **5h**

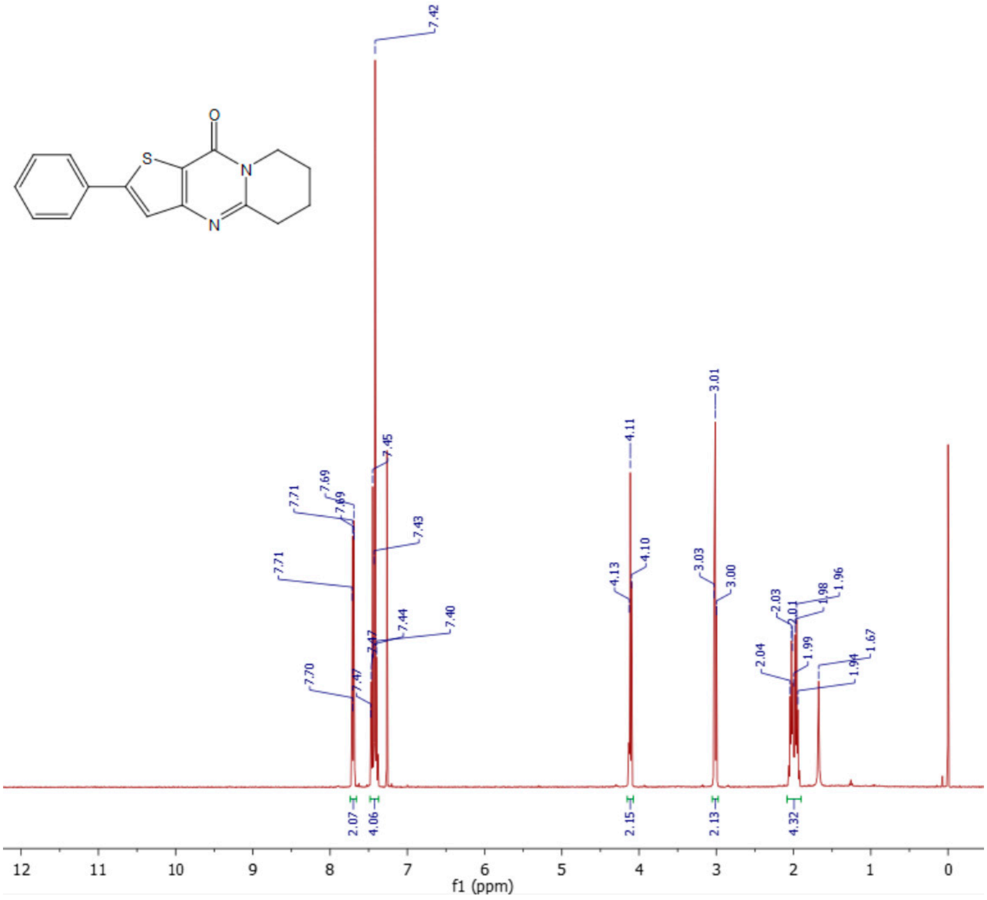

Figure S17: ^{1}H NMR spectrum of compound **5i**

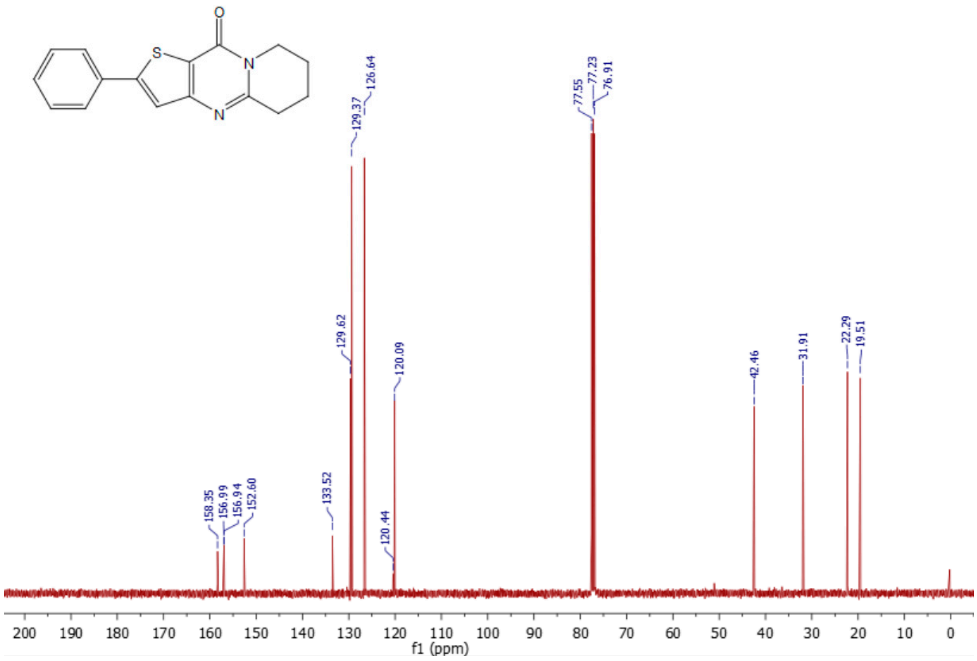

Figure S18: ^{13}C NMR spectrum of compound **5i**

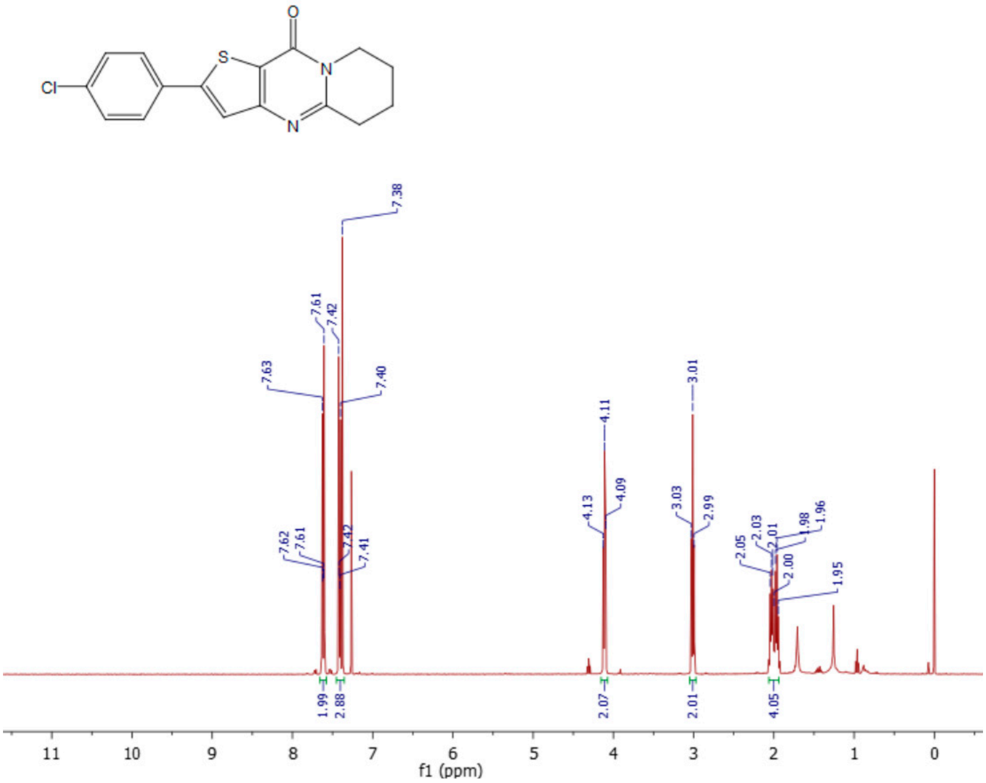

Figure S19: ^{1}H NMR spectrum of compound **5j**

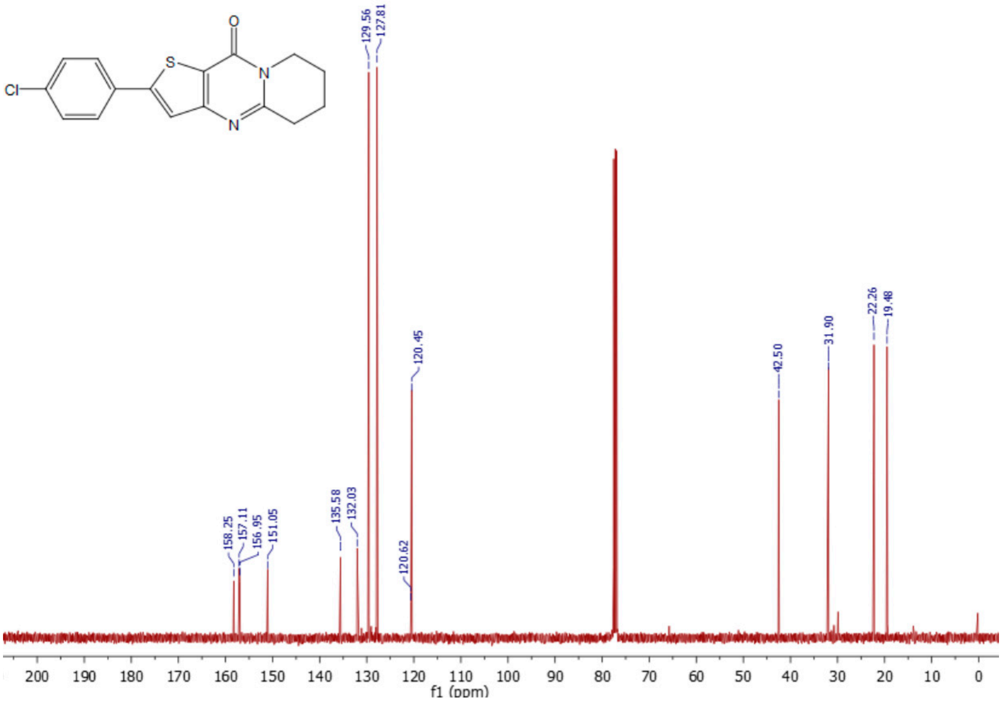

Figure S20: ^{13}C NMR spectrum of compound **5j**

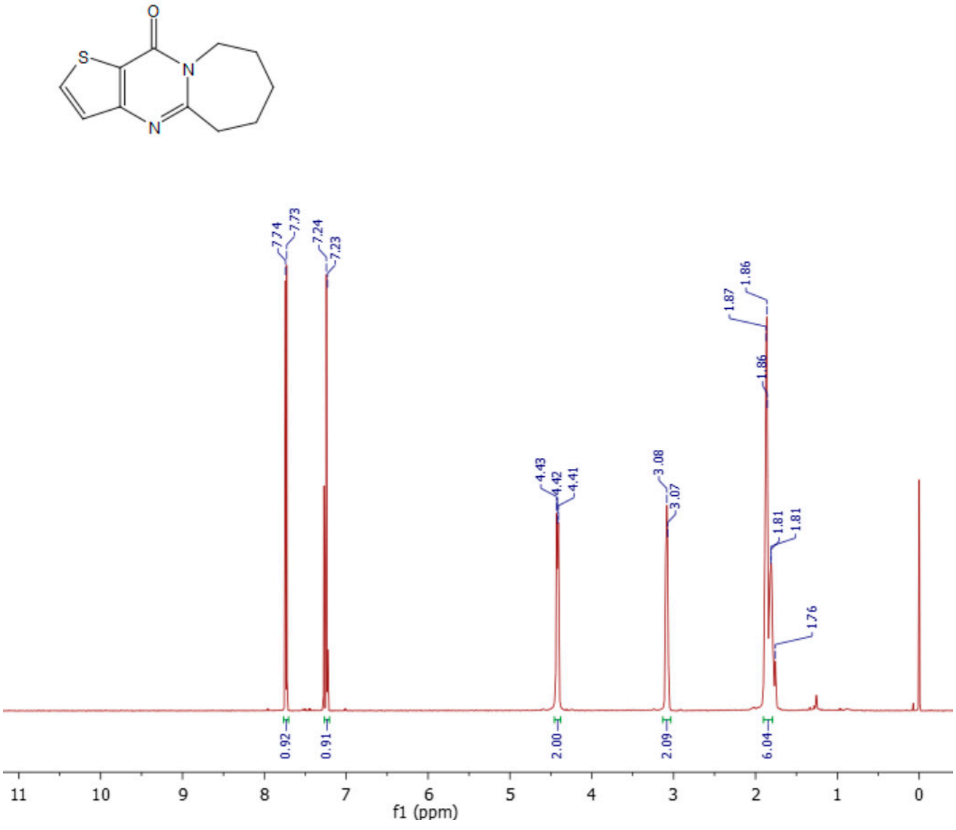

Figure S21: ^{1}H NMR spectrum of compound **5k**

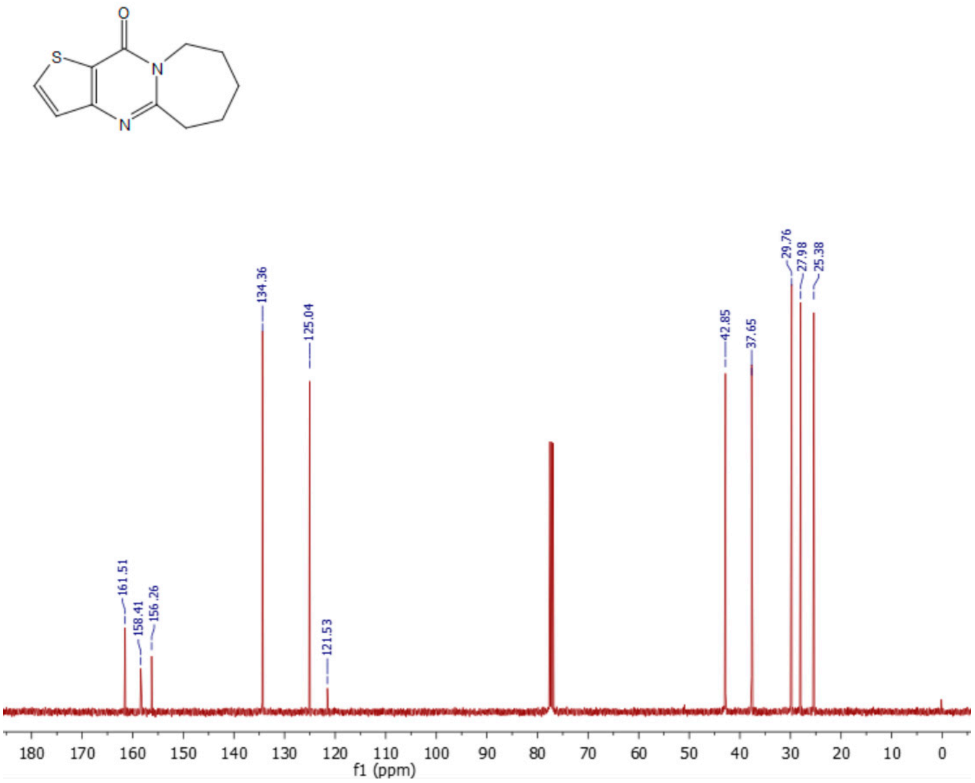

Figure S22: ^{13}C NMR spectrum of compound **5k**

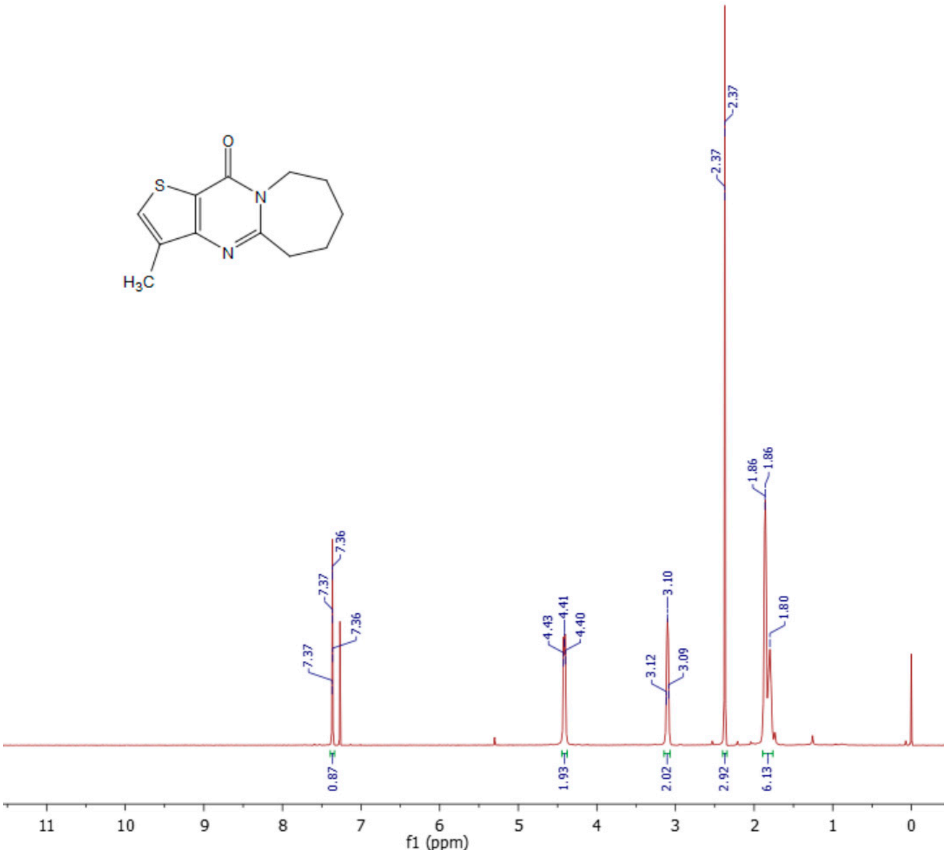

Figure S23: ^{1}H NMR spectrum of compound **5l**

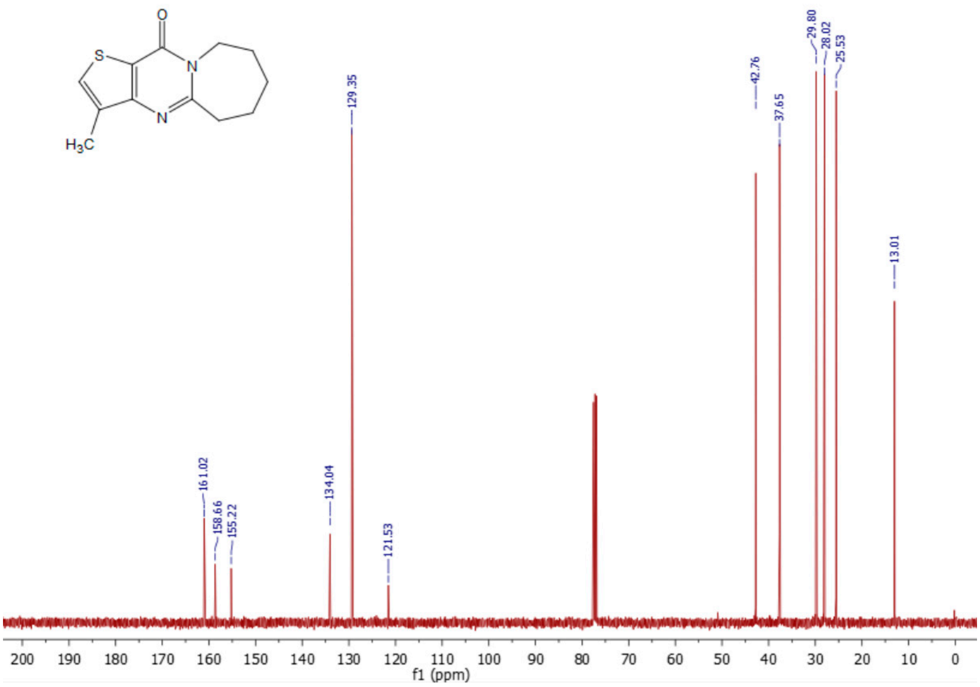

Figure S24: ^{13}C NMR spectrum of compound **5l**

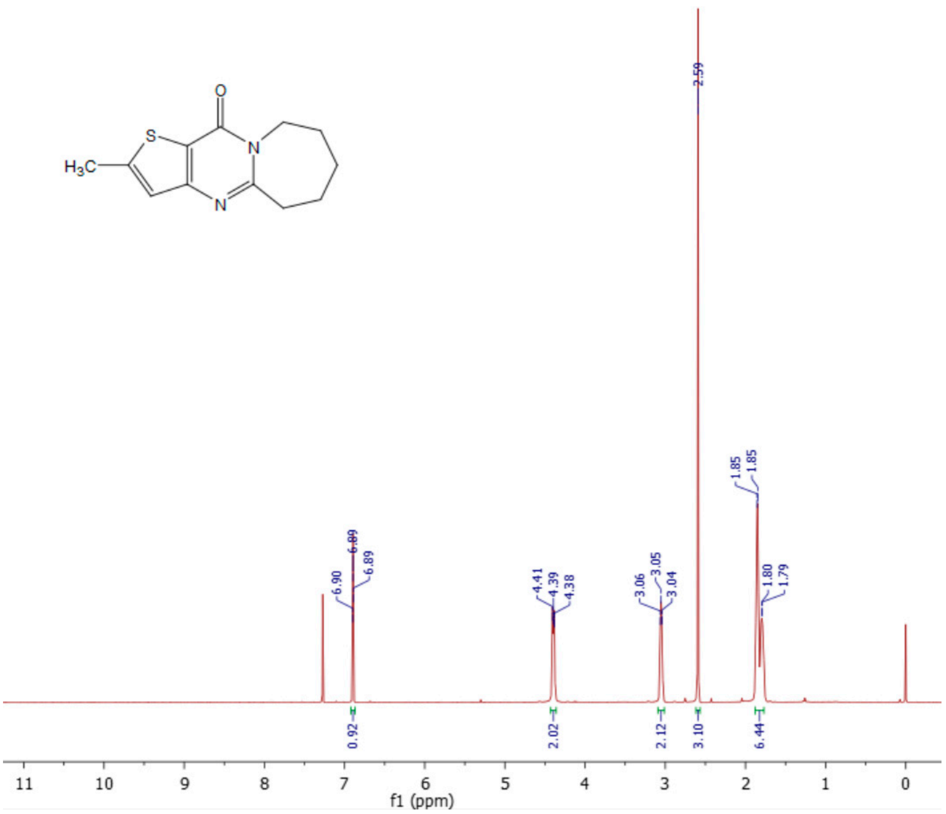

Figure S25: ^{1}H NMR spectrum of compound **5m**

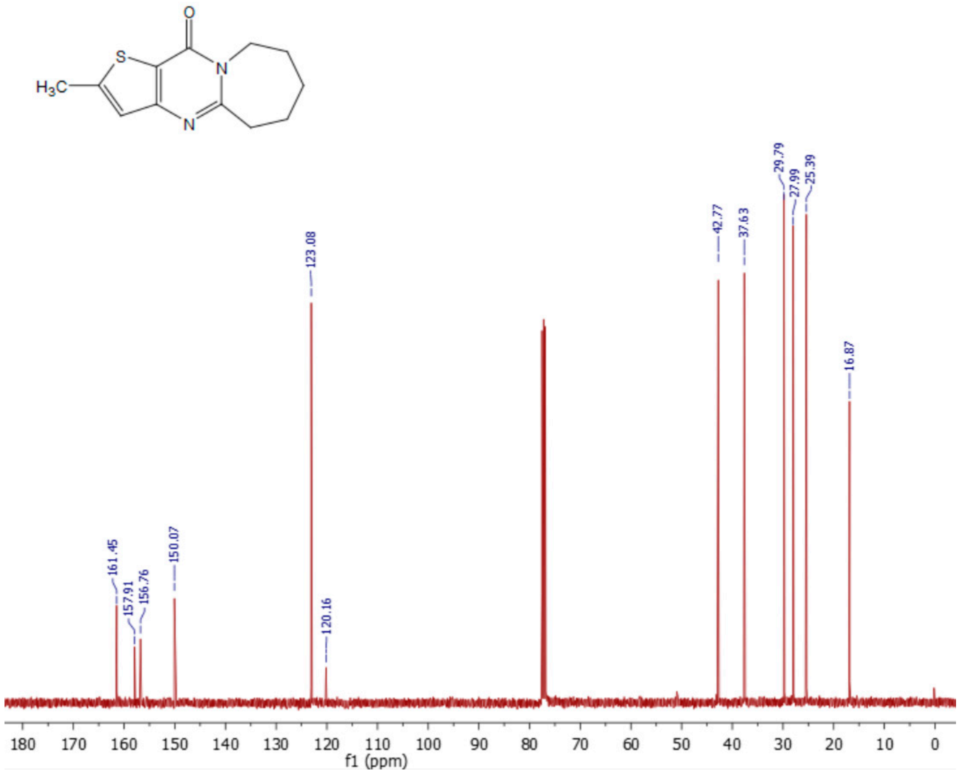

Figure S26: ^{13}C NMR spectrum of compound **5m**

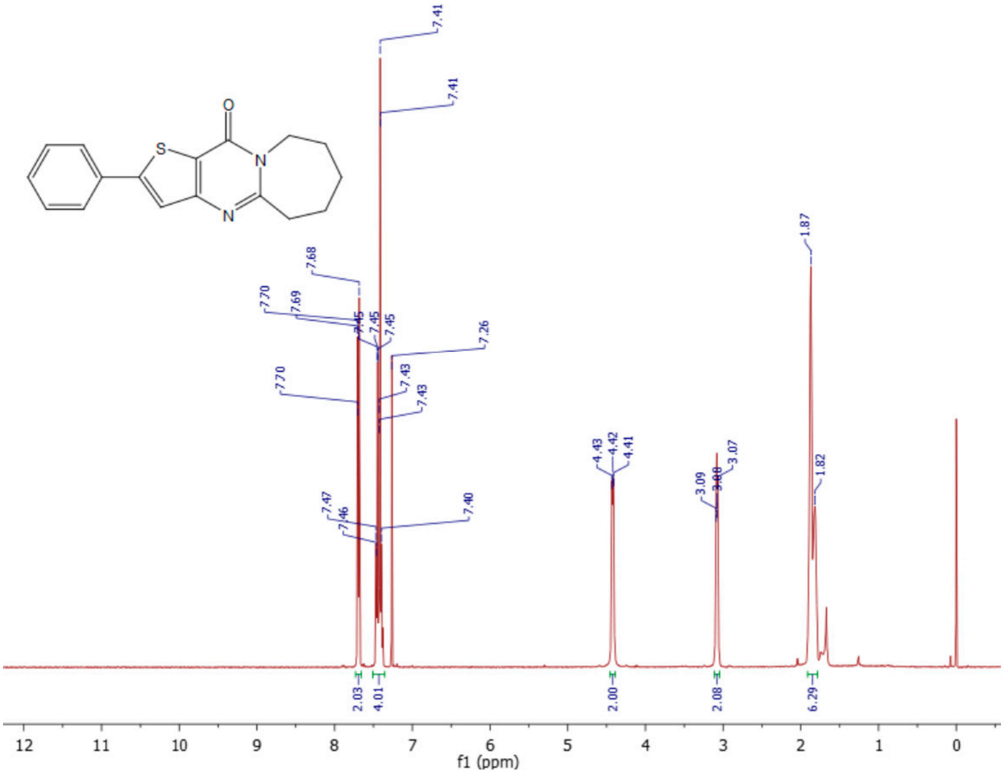

Figure S27: ^{1}H NMR spectrum of compound **5n**

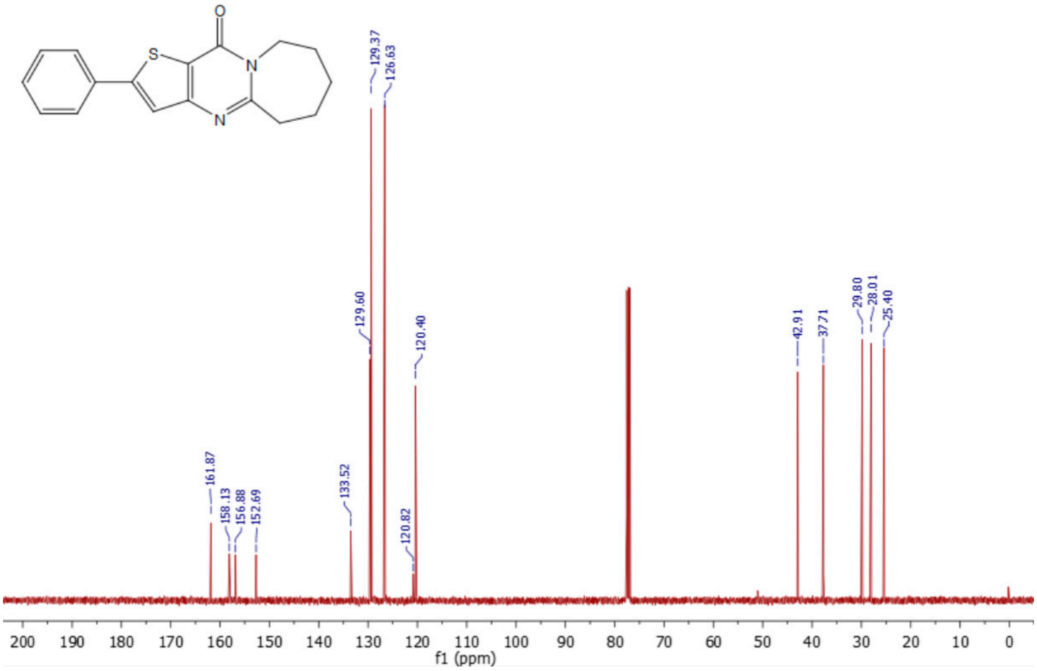

Figure S28: ^{13}C NMR spectrum of compound **5n**

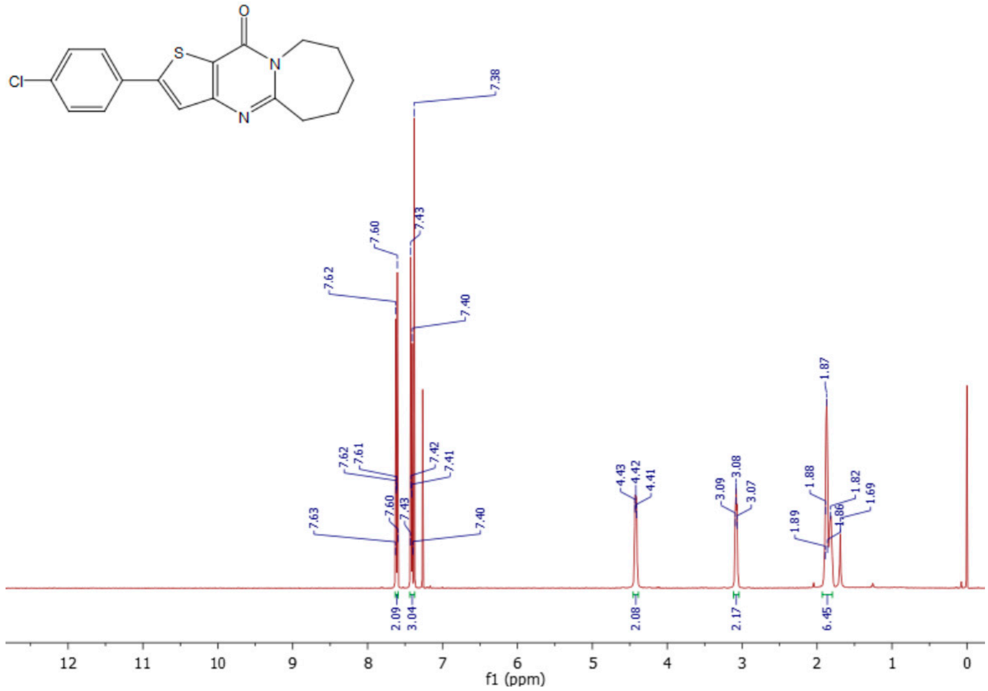

Figure S29: ^{1}H NMR spectrum of compound **5o**

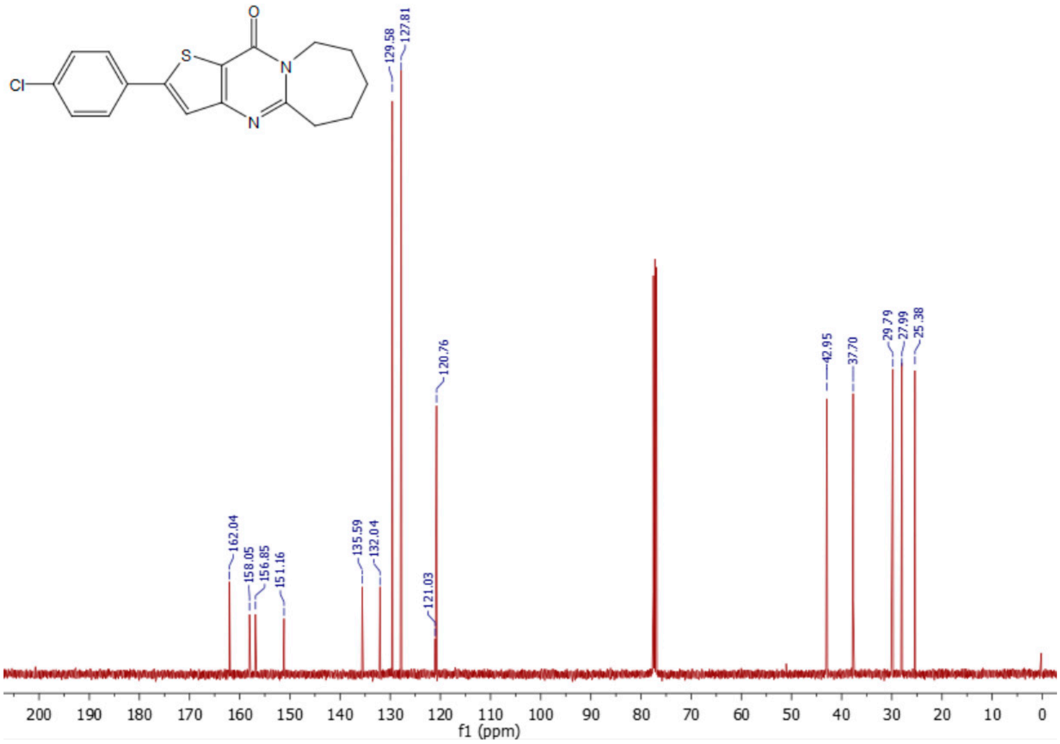

Figure S30: ^{13}C NMR spectrum of compound **5o**

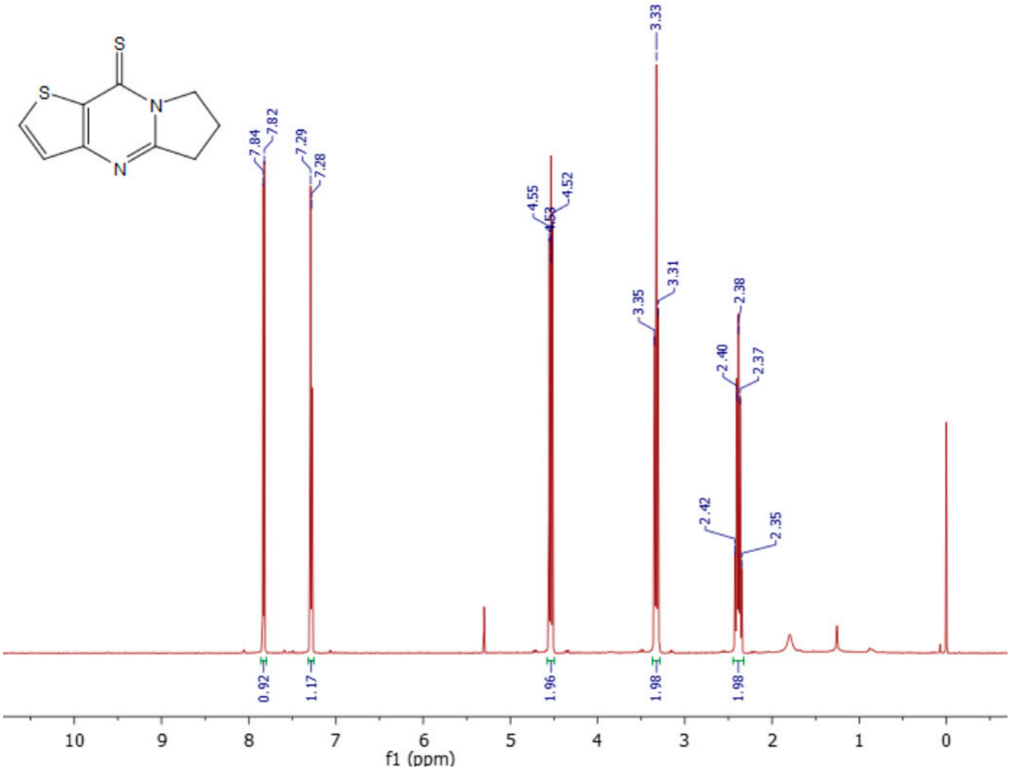

Figure S31: ^{1}H NMR spectrum of compound **6a**

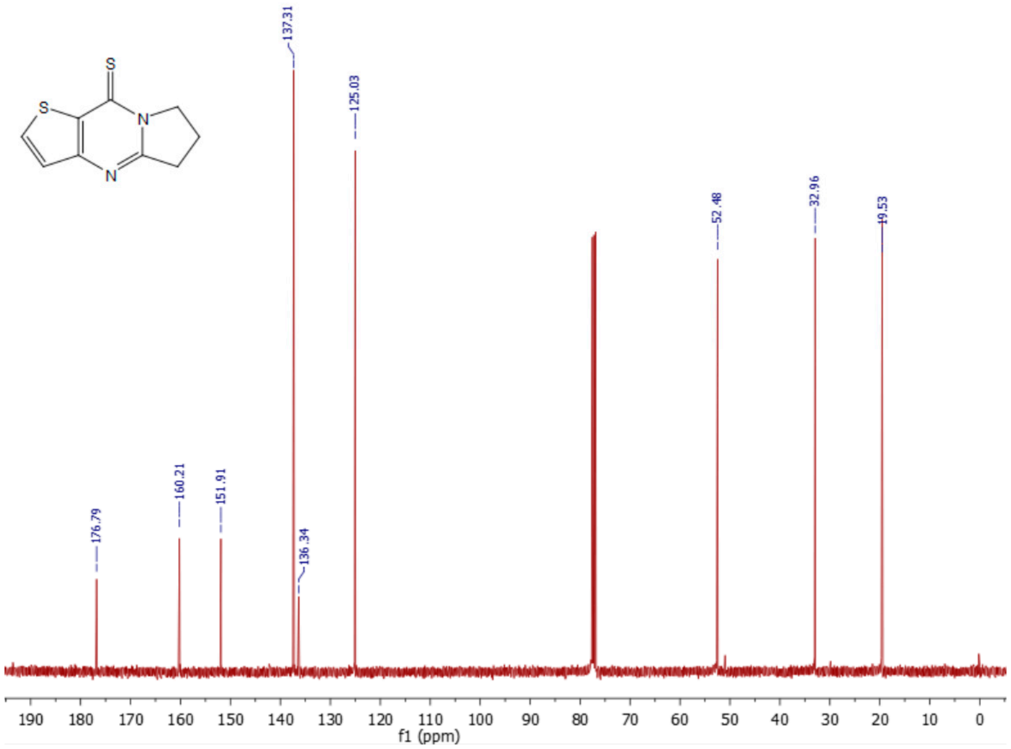

Figure S32: ^{13}C NMR spectrum of compound **6a**

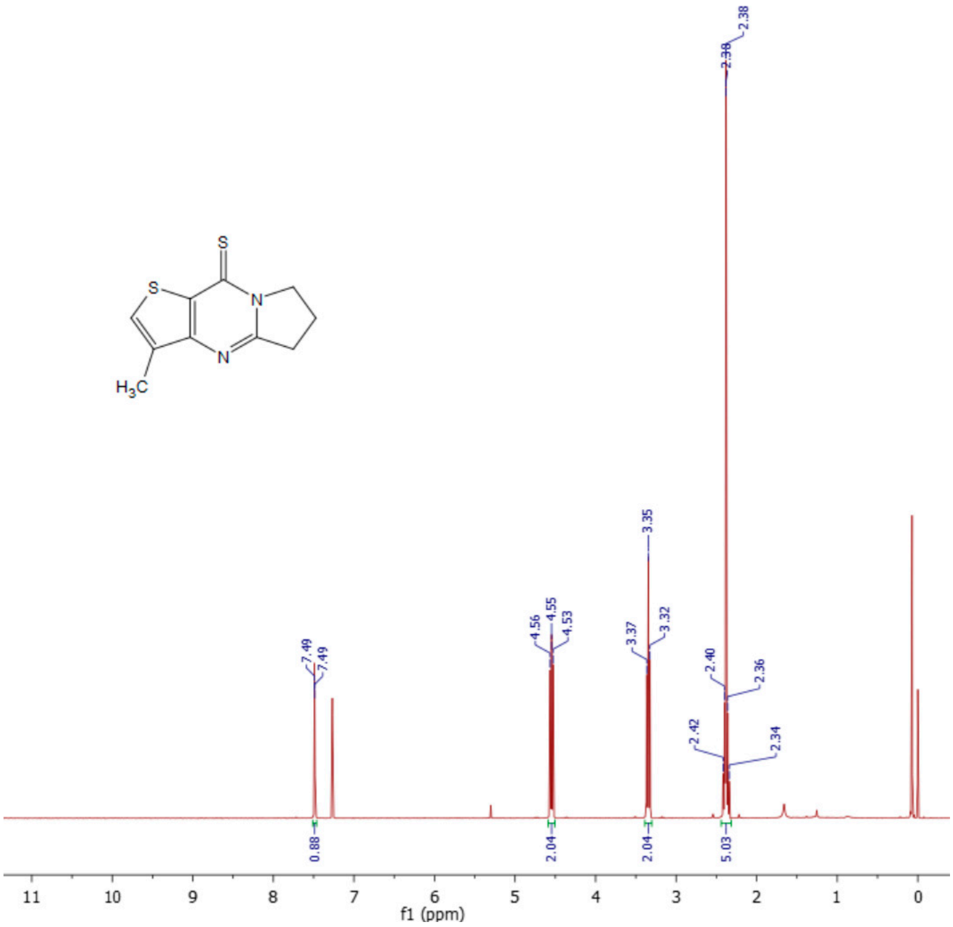

Figure S33: ^{1}H NMR spectrum of compound **6b**

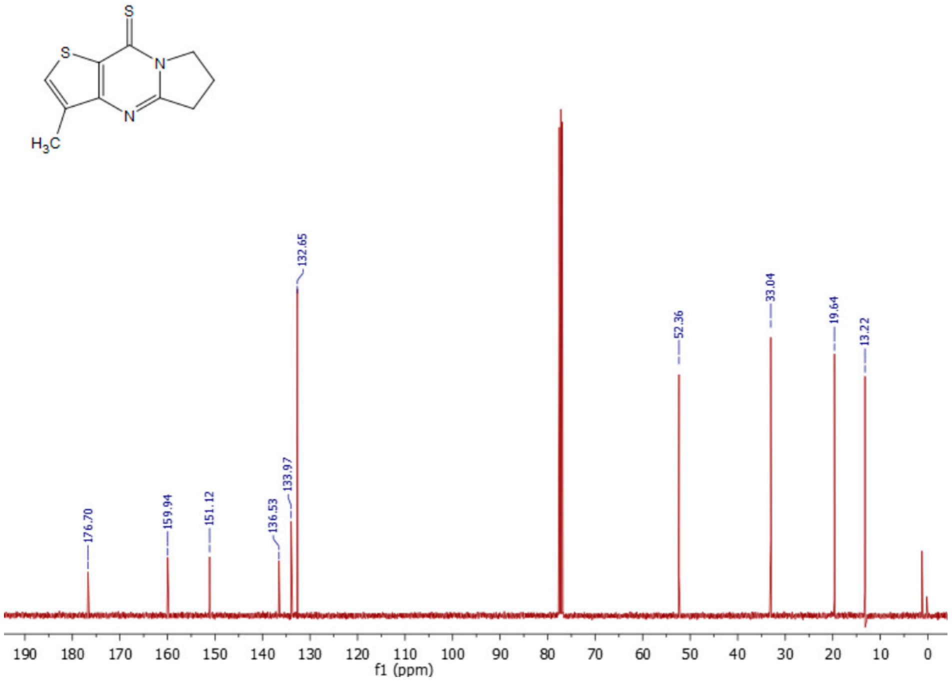

Figure S34: ^{13}C NMR spectrum of compound **6b**

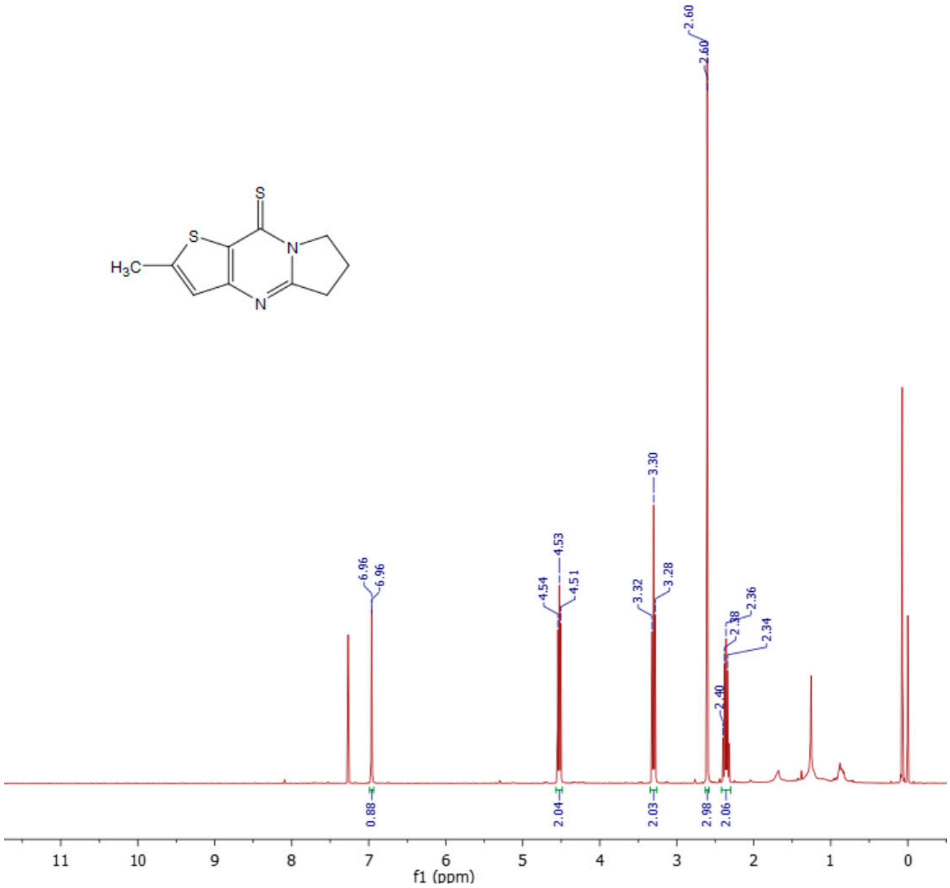

Figure S35: ^{1}H NMR spectrum of compound **6c**

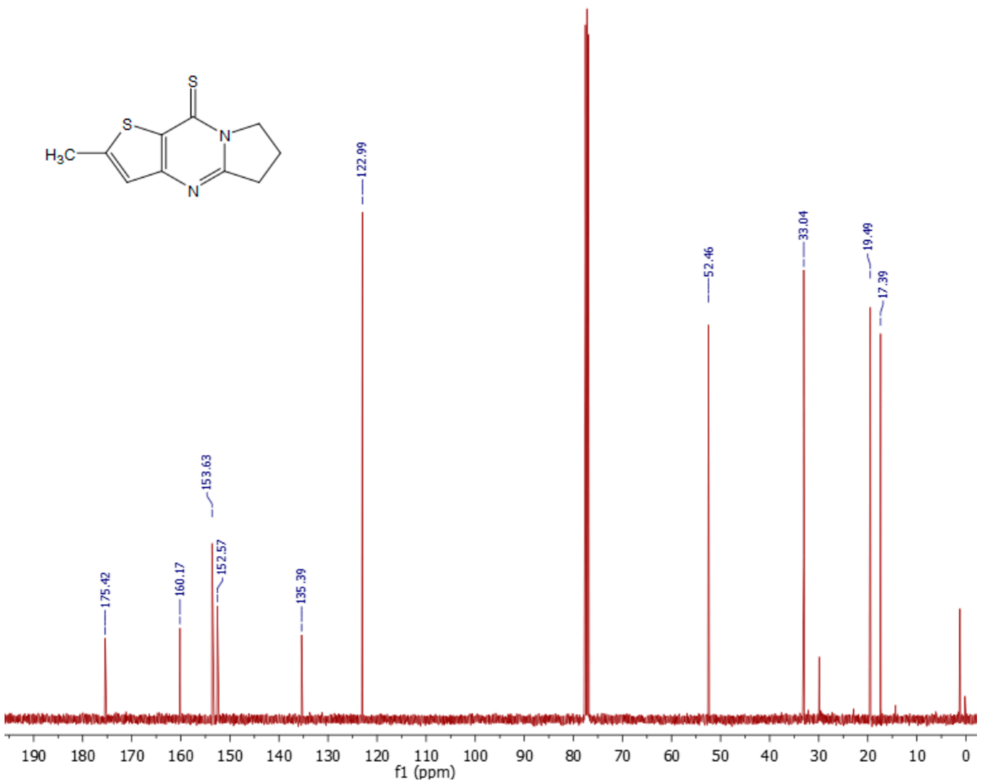

Figure S36: ^{13}C NMR spectrum of compound **6c**

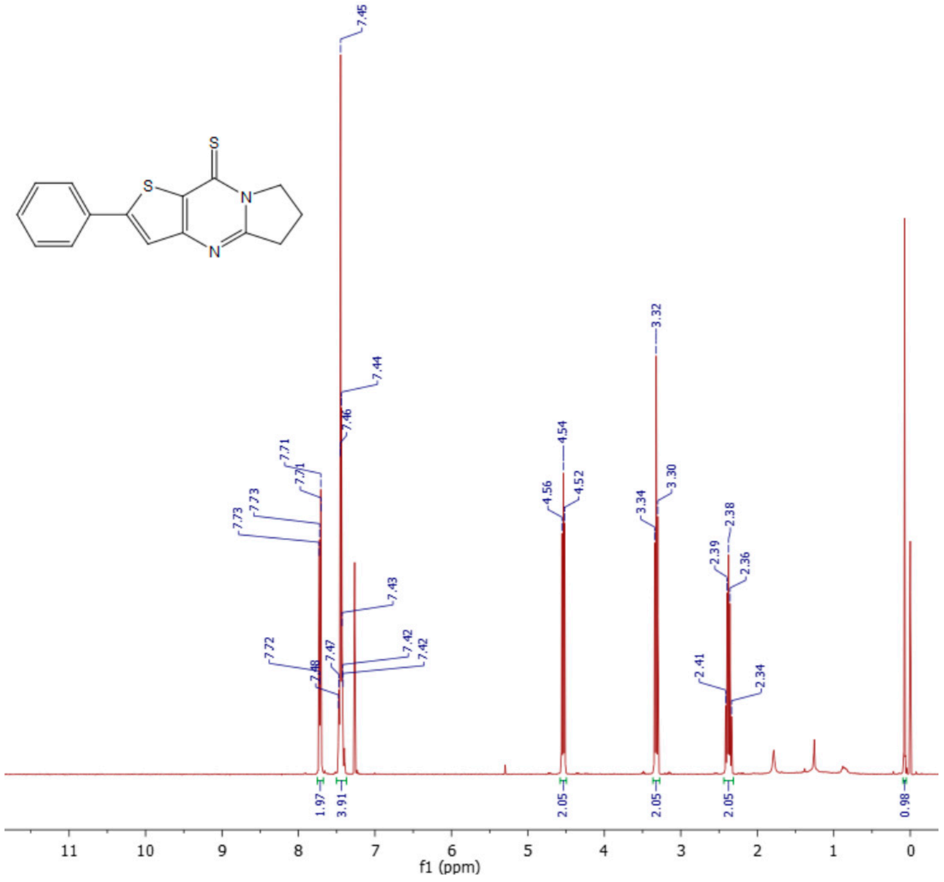

Figure S37: ^{1}H NMR spectrum of compound **6d**

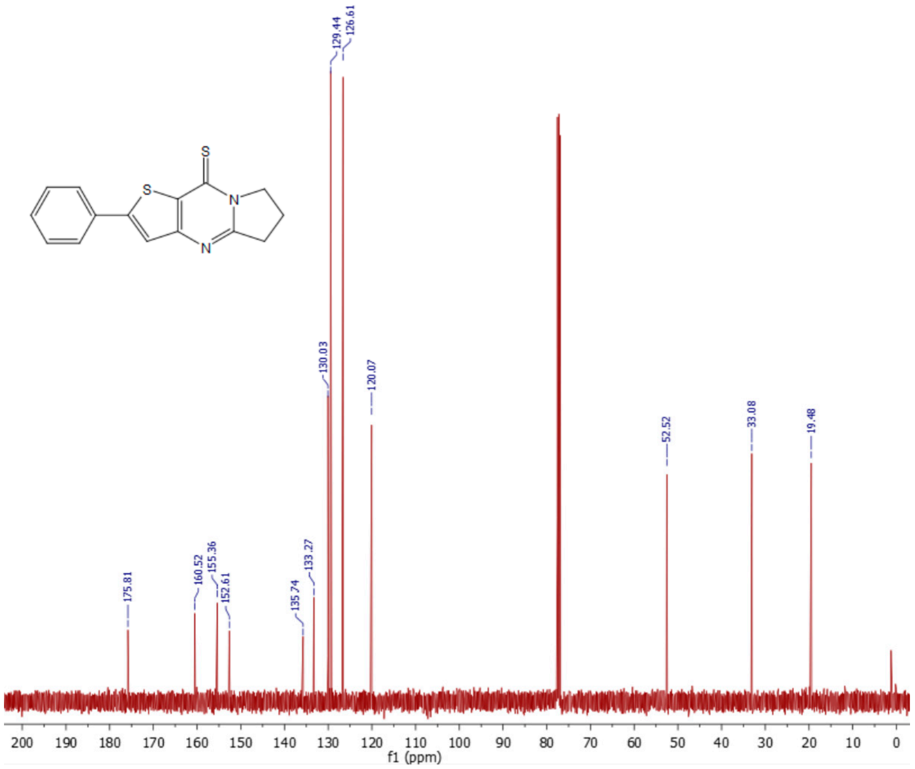

Figure S38: ^{13}C NMR spectrum of compound **6d**

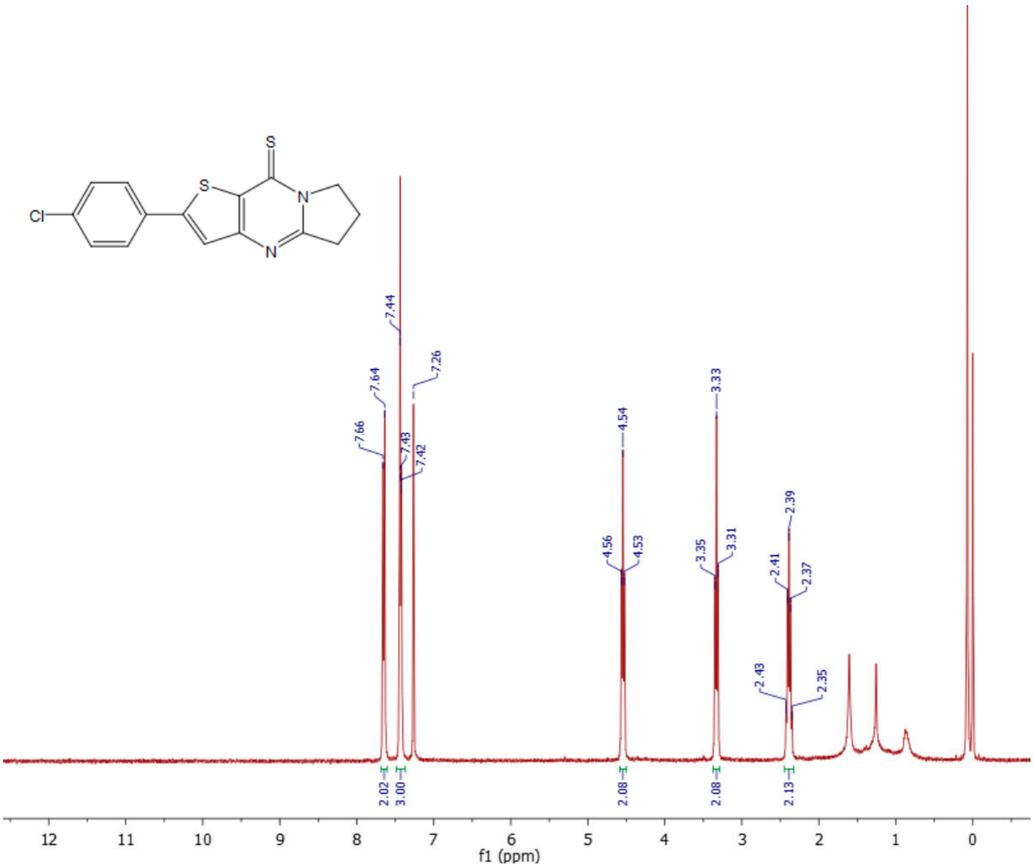

Figure S39: ^{1}H NMR spectrum of compound **6e**

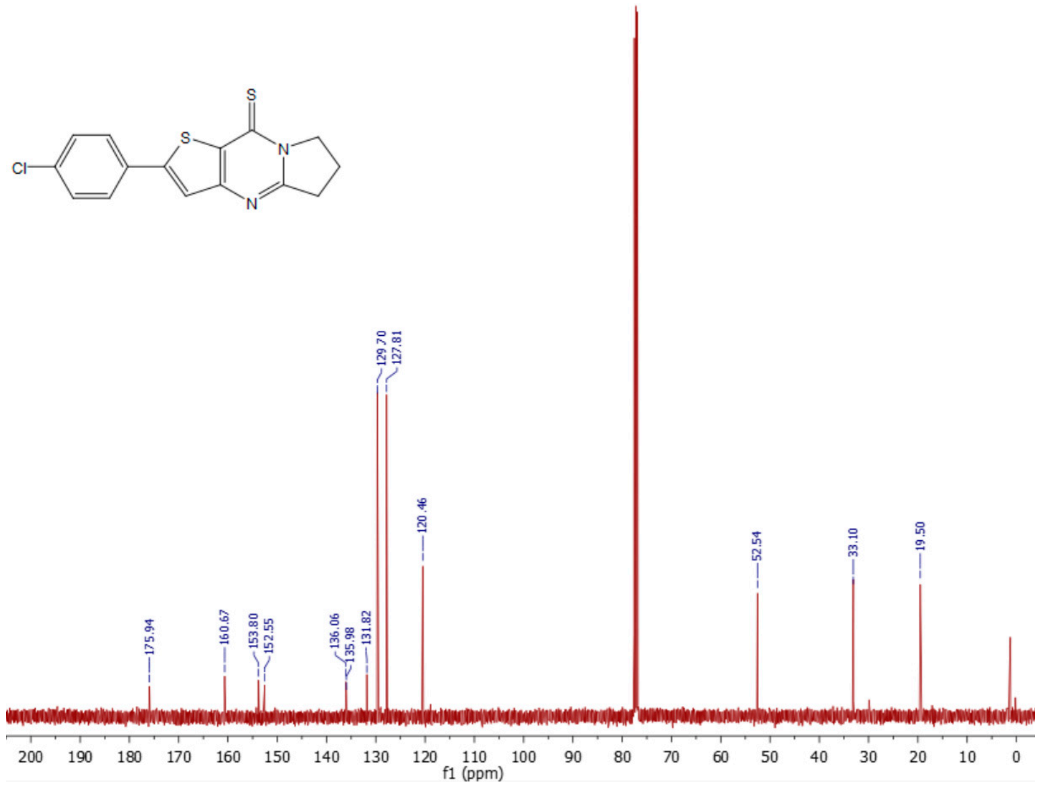

Figure S40: ^{13}C NMR spectrum of compound **6e**

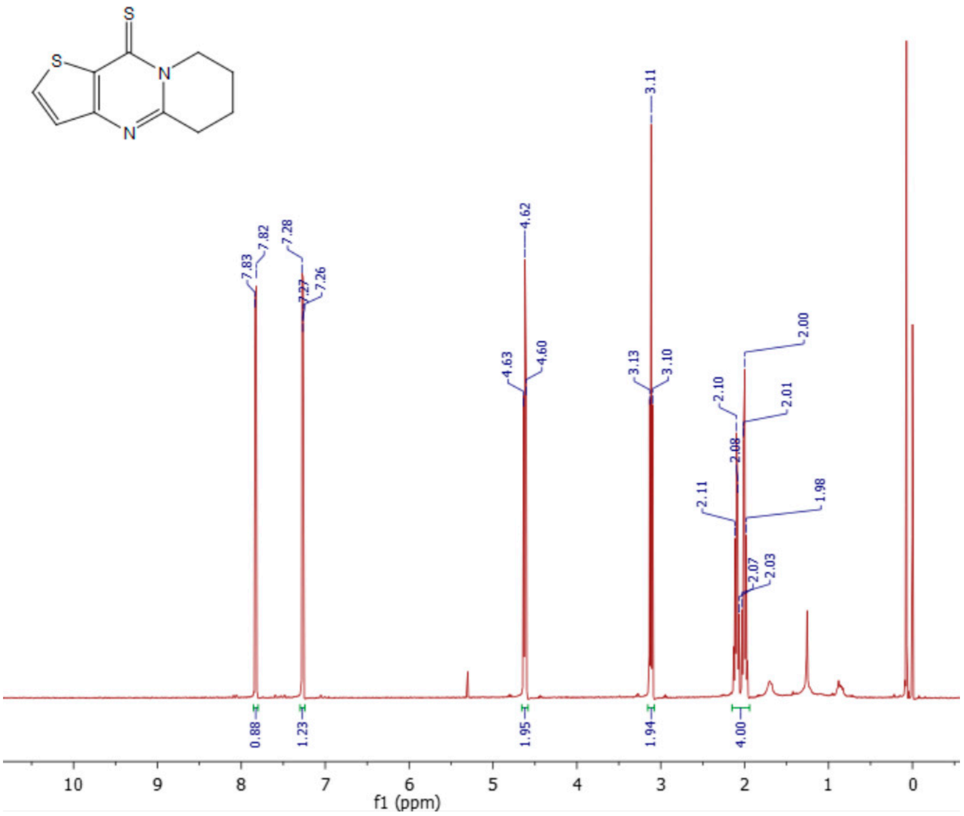

Figure S41: ^{1}H NMR spectrum of compound **6f**

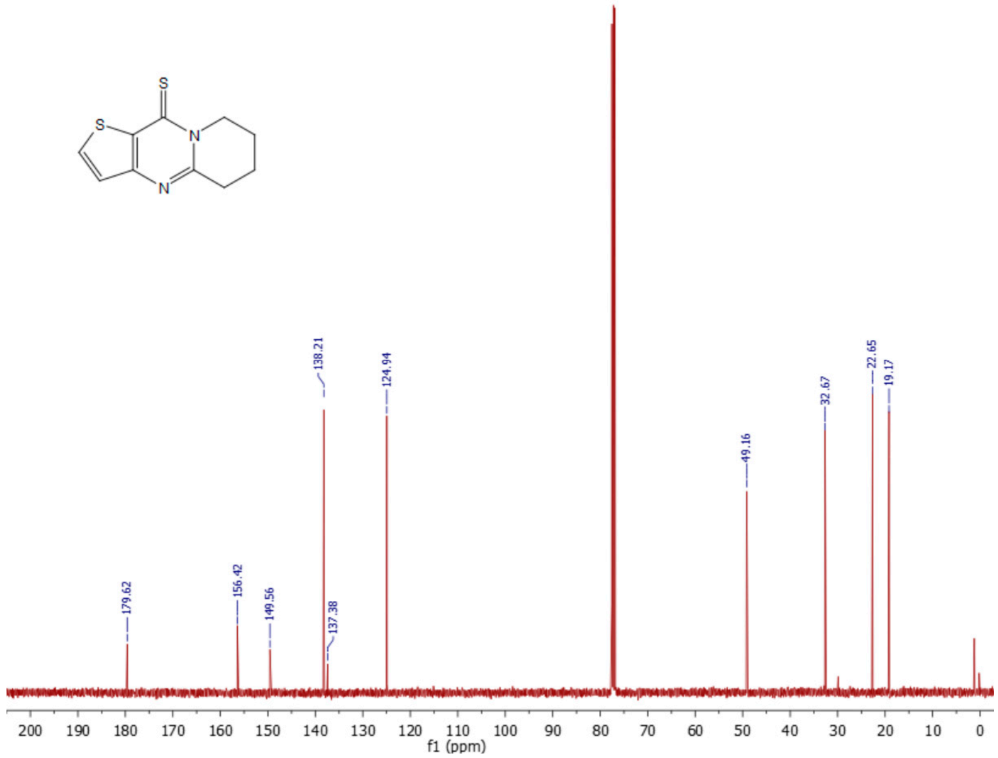

Figure S42: ^{13}C NMR spectrum of compound **6f**

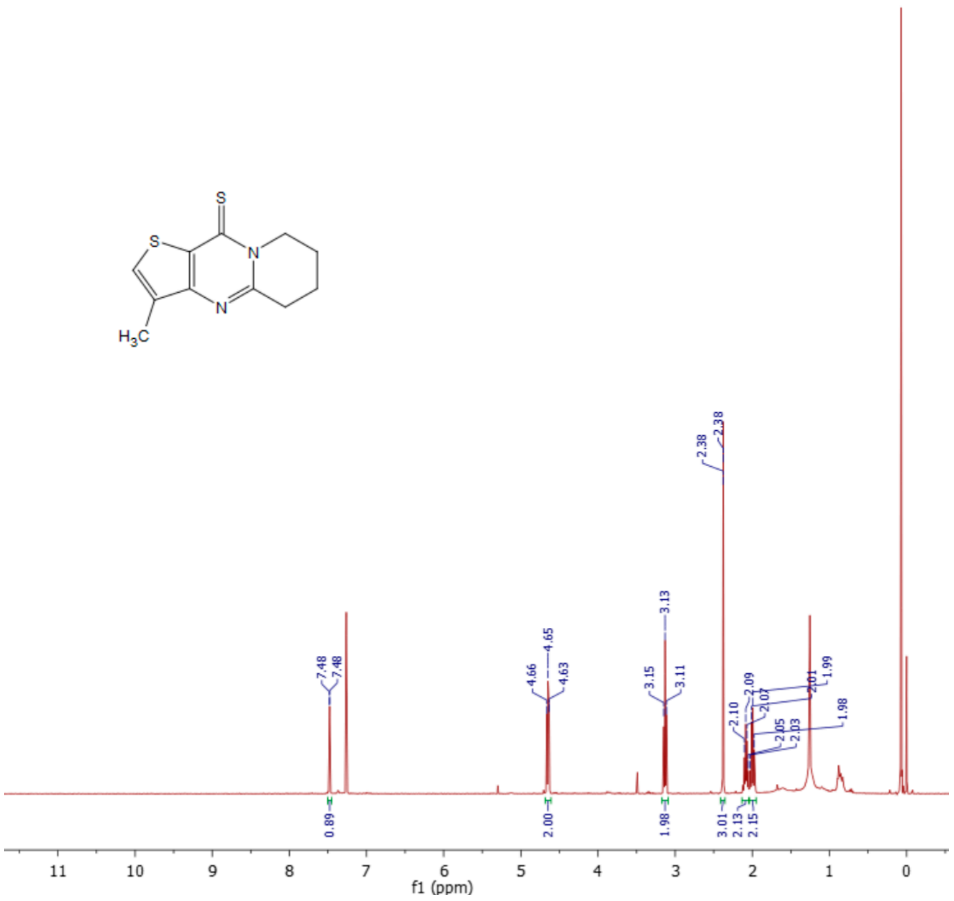

Figure S43: ^{1}H NMR spectrum of compound **6g**

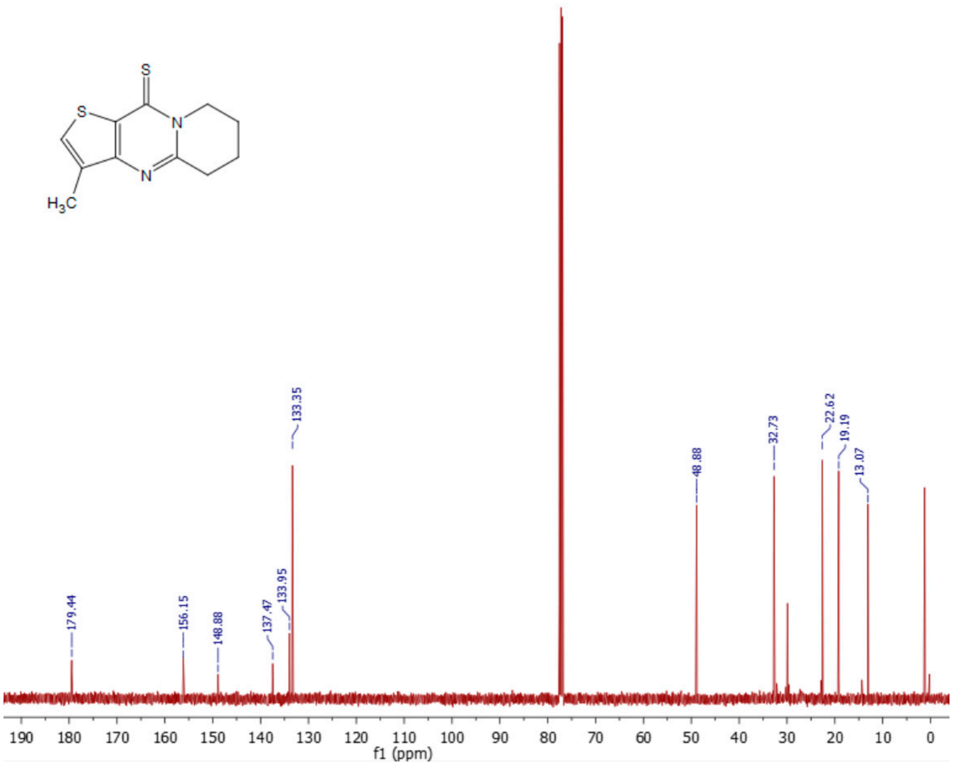

Figure S44: ^{13}C NMR spectrum of compound **6g**

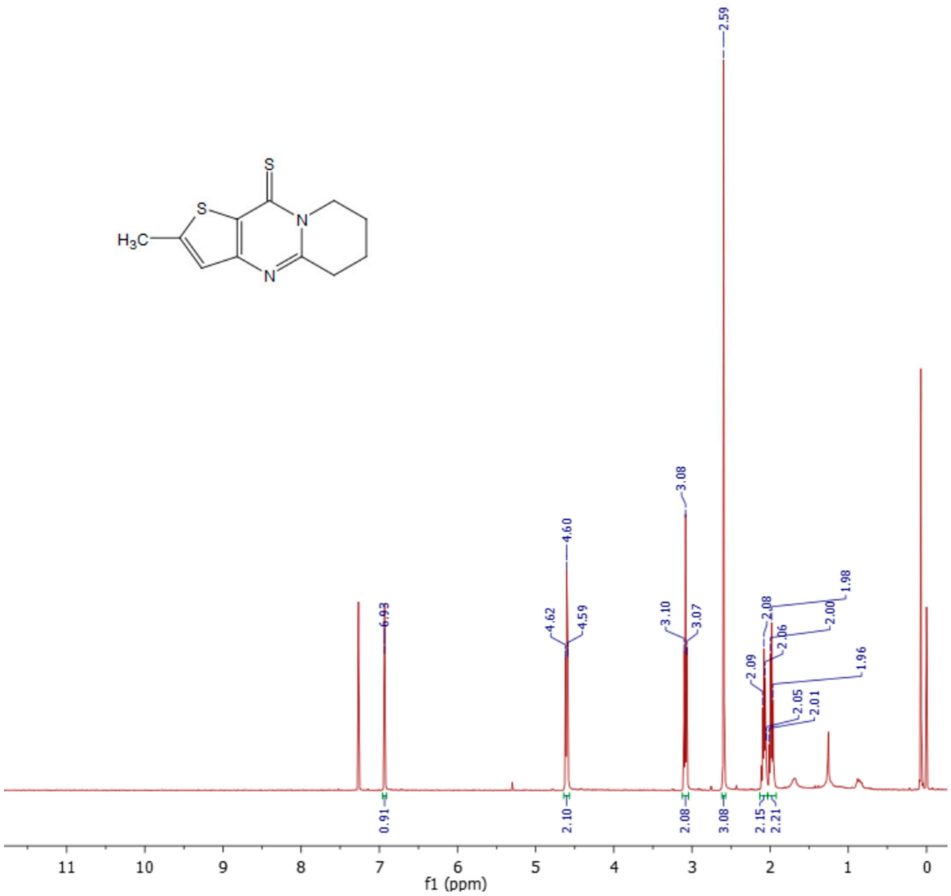

Figure S45: ^{1}H NMR spectrum of compound **6h**

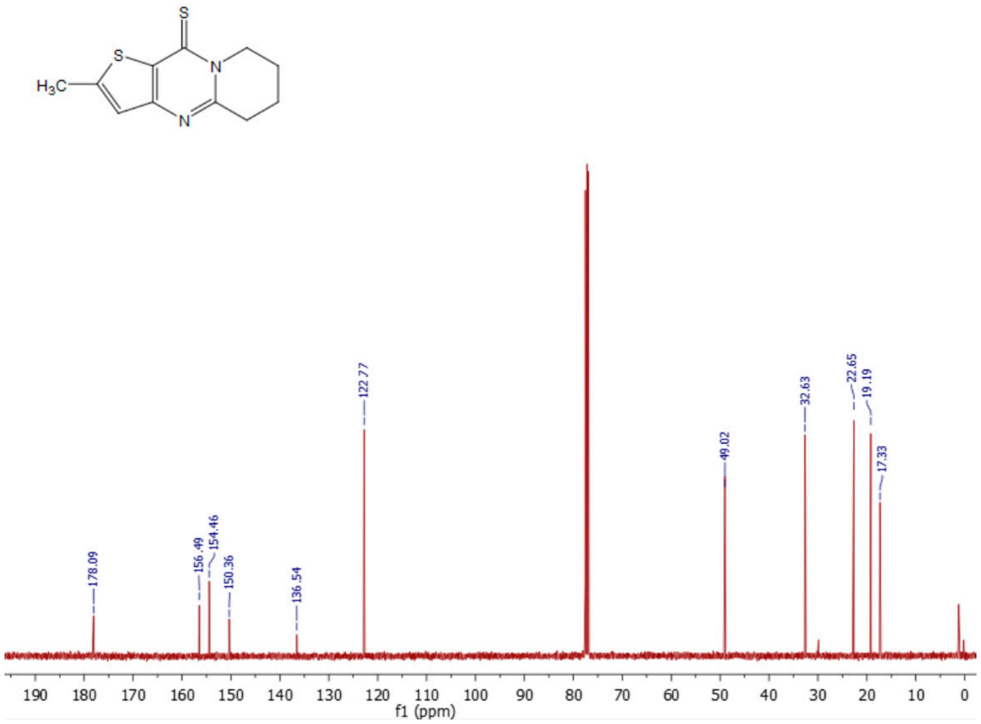

Figure S46: ^{13}C NMR spectrum of compound **6h**

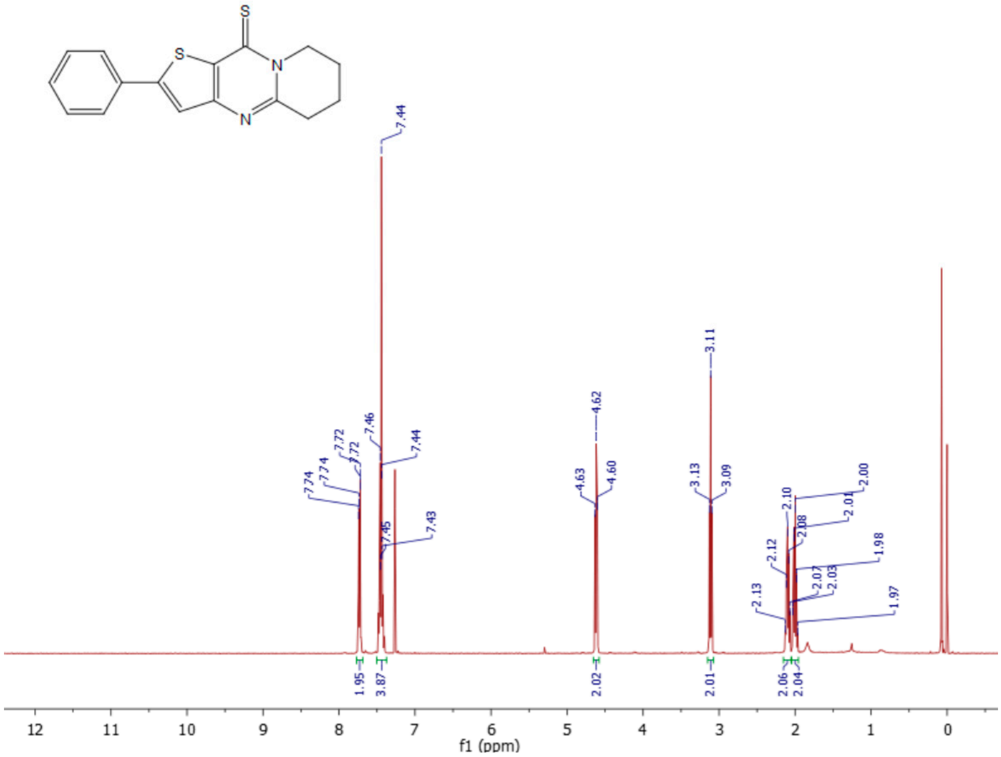

Figure S47: ^{1}H NMR spectrum of compound **6i**

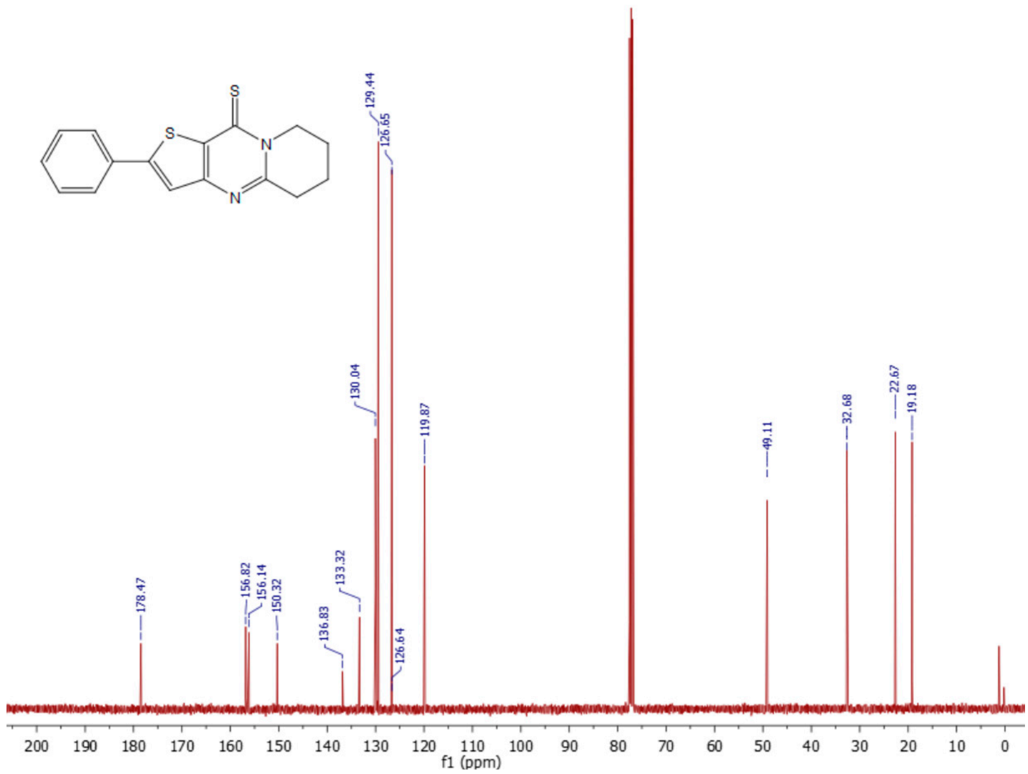

Figure S48: ^{13}C NMR spectrum of compound **6i**

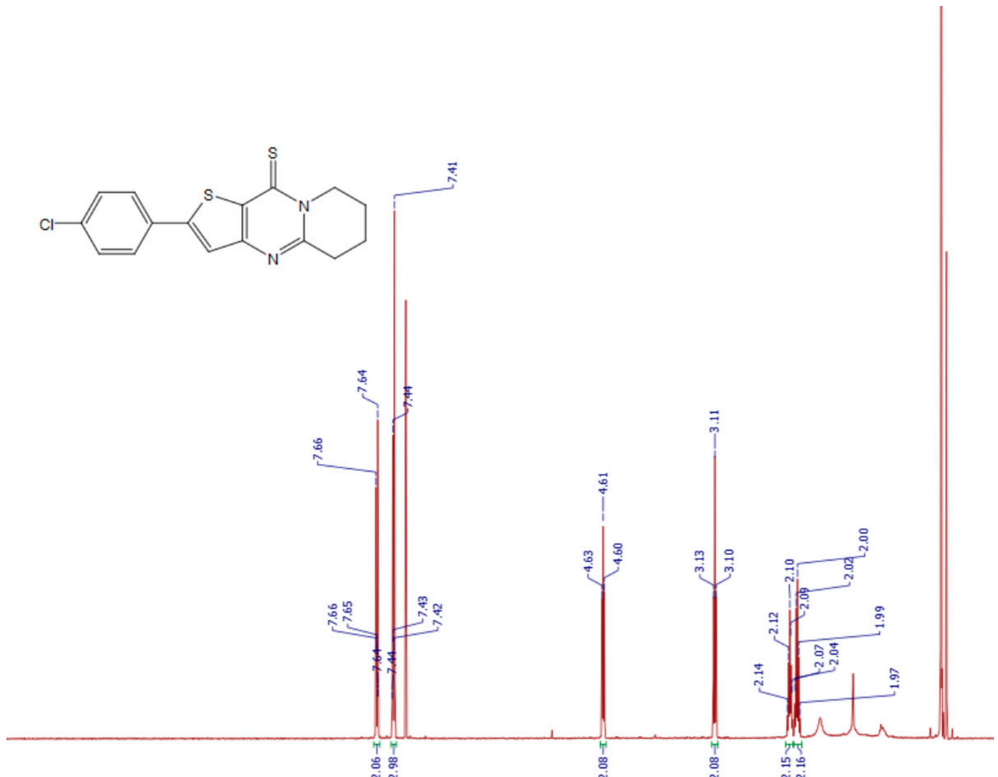

Figure S49: ^{1}H NMR spectrum of compound **6j**

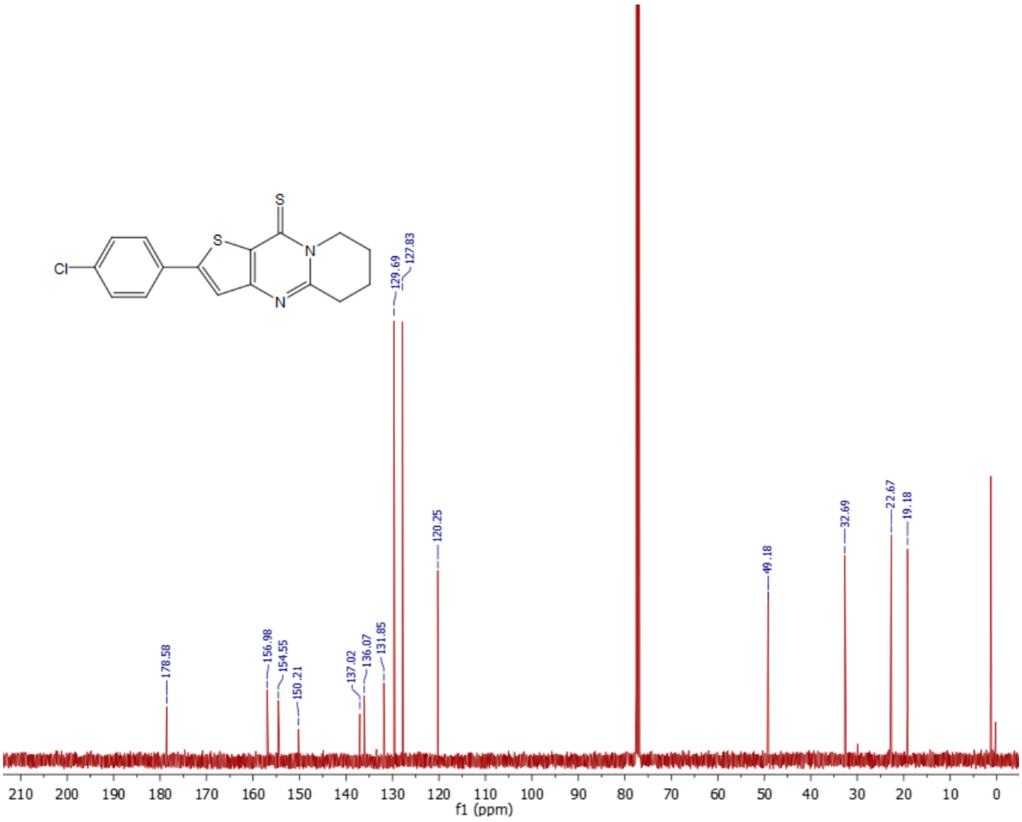

Figure S50: ^{13}C NMR spectrum of compound **6j**

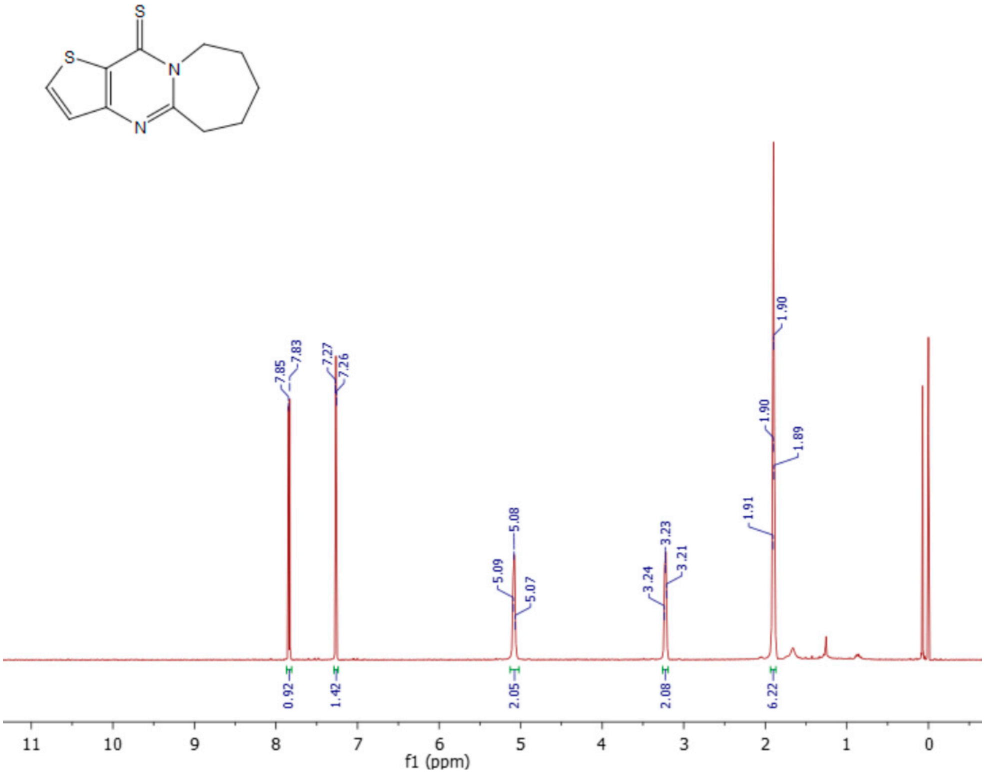

Figure S51: ^{1}H NMR spectrum of compound **6k**

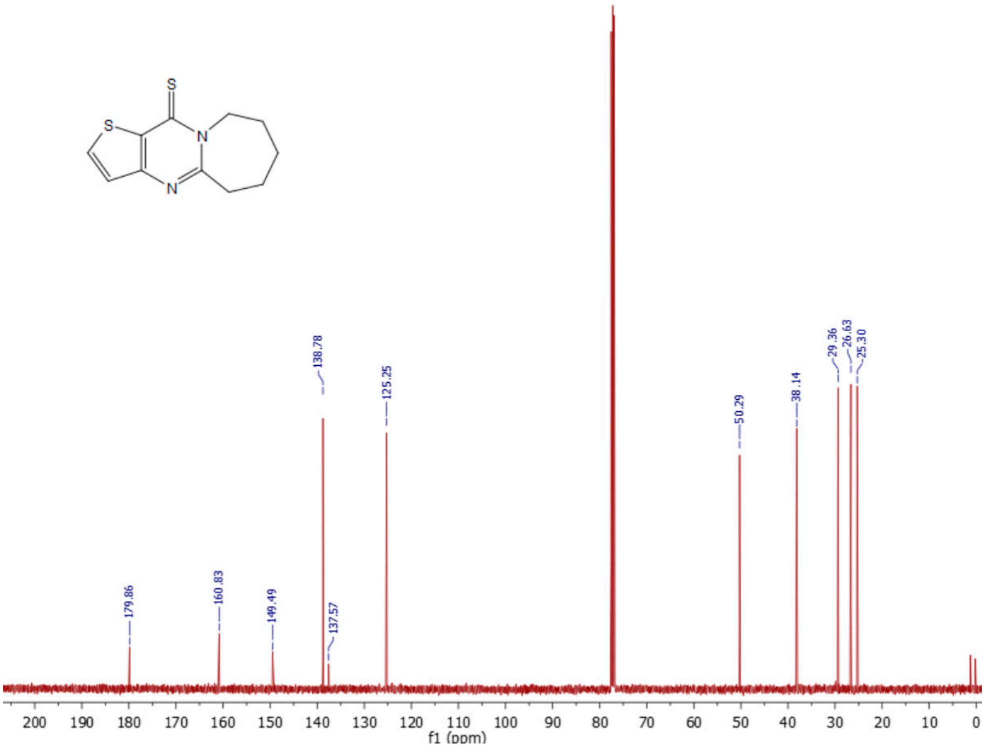

Figure S52: ^{13}C NMR spectrum of compound **6k**

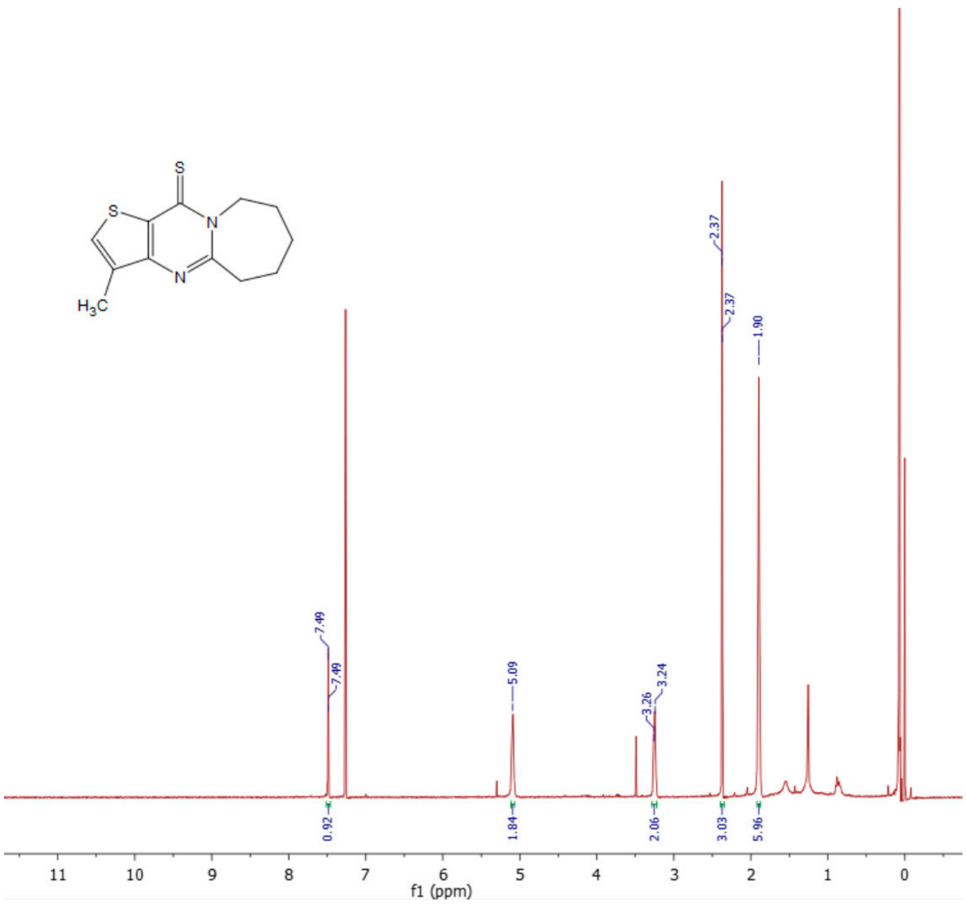

Figure S53: ^{1}H NMR spectrum of compound **6l**

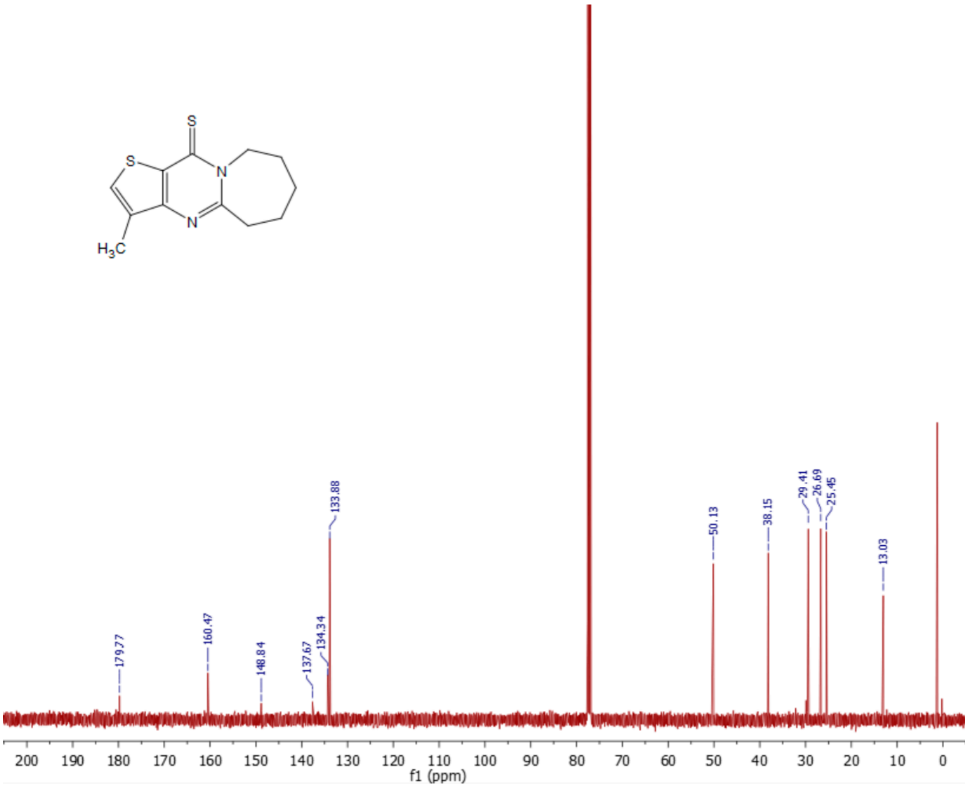

Figure S54: ^{13}C NMR spectrum of compound **6l**

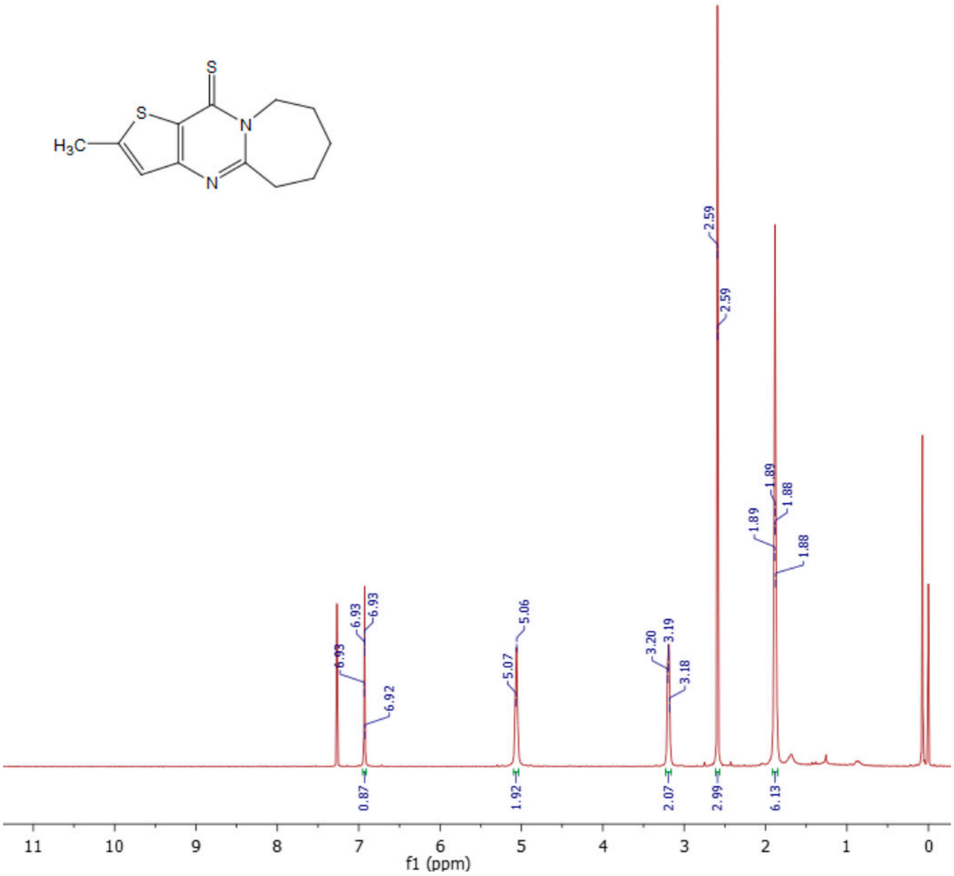

Figure S55: ^{1}H NMR spectrum of compound **6m**

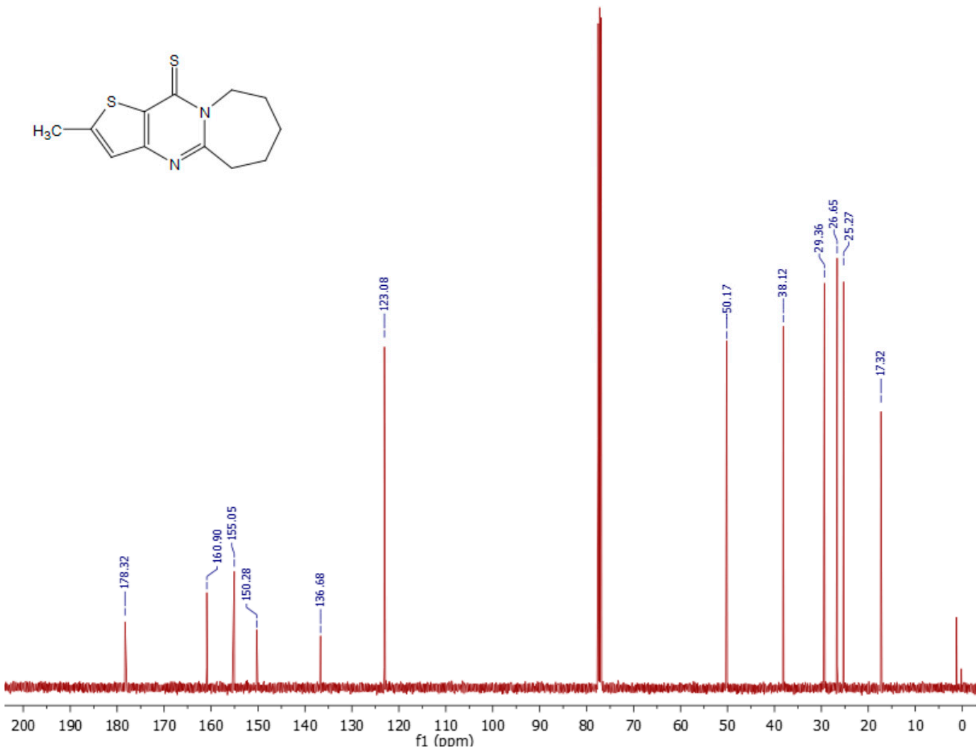

Figure S56: ^{13}C NMR spectrum of compound **6m**

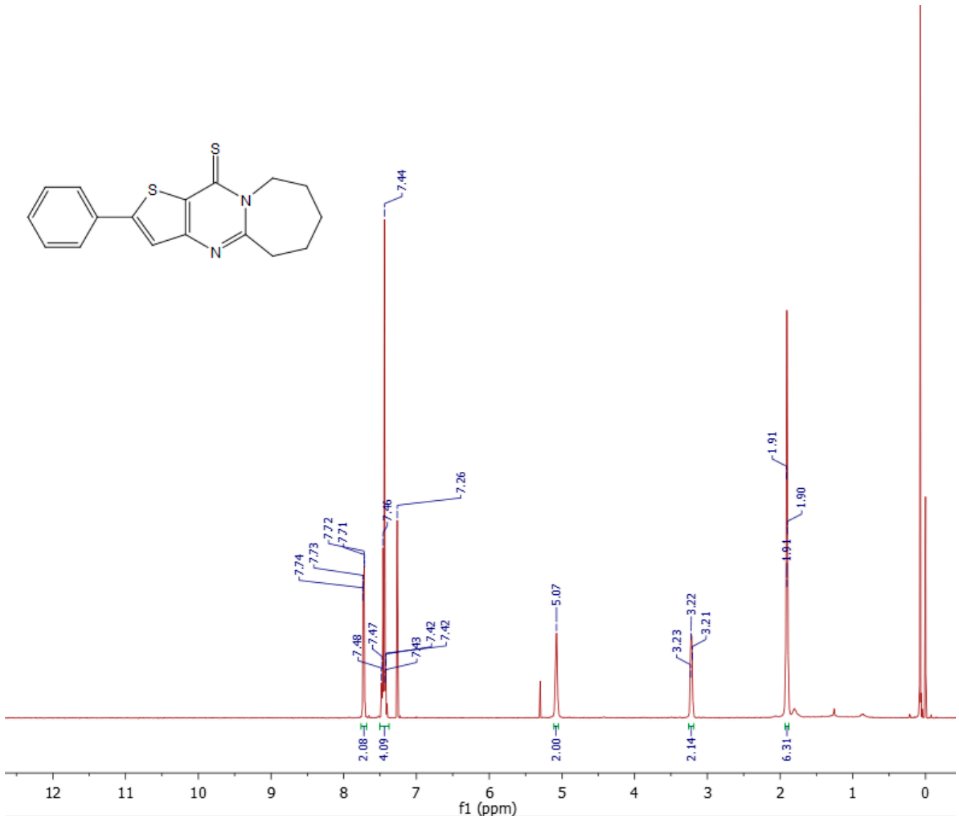

Figure S57: ^{1}H NMR spectrum of compound **6n**

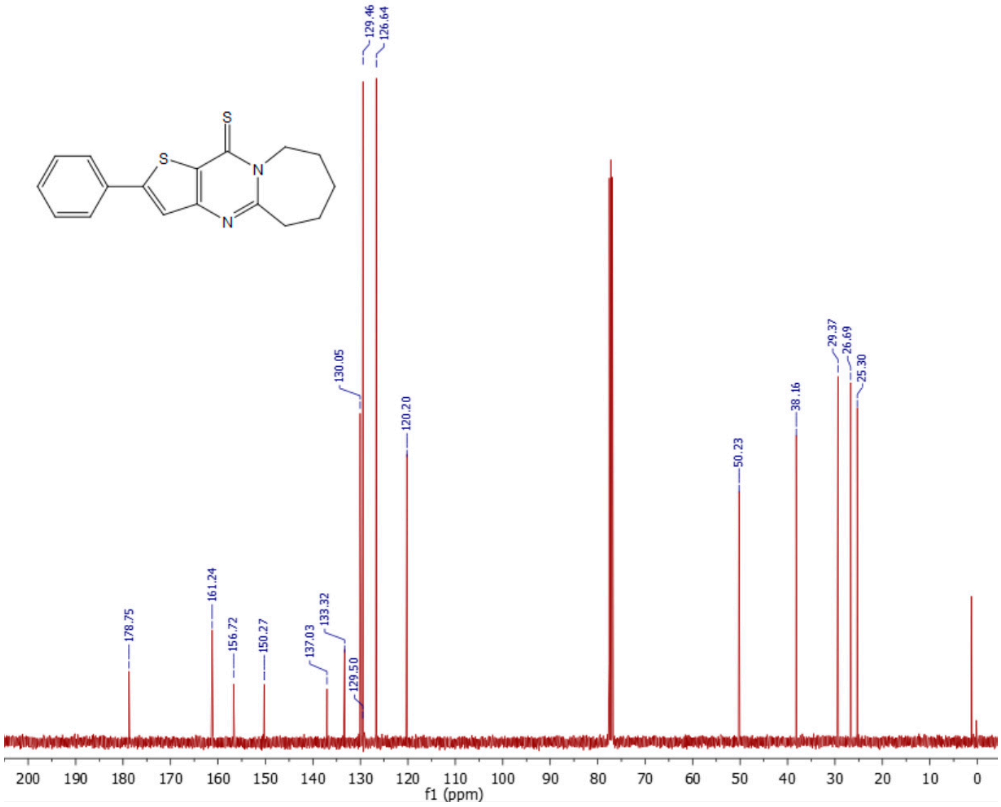

Figure S58: ^{13}C NMR spectrum of compound **6n**

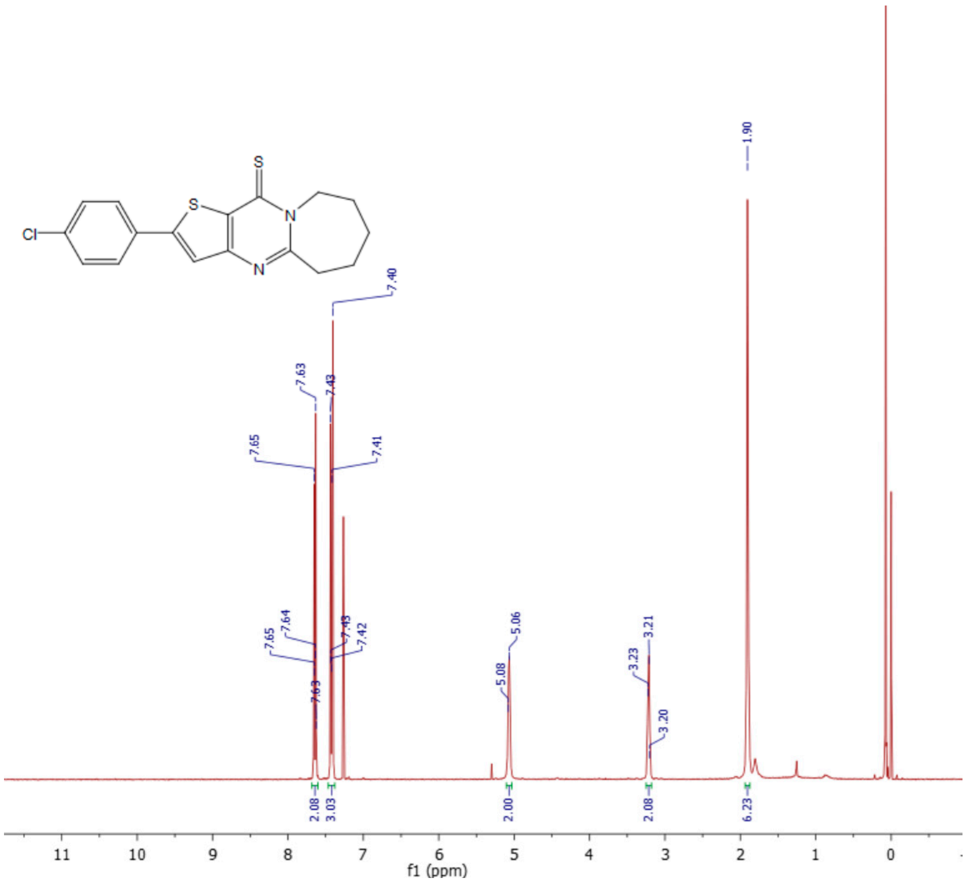

Figure S59: ^{1}H NMR spectrum of compound **6o**

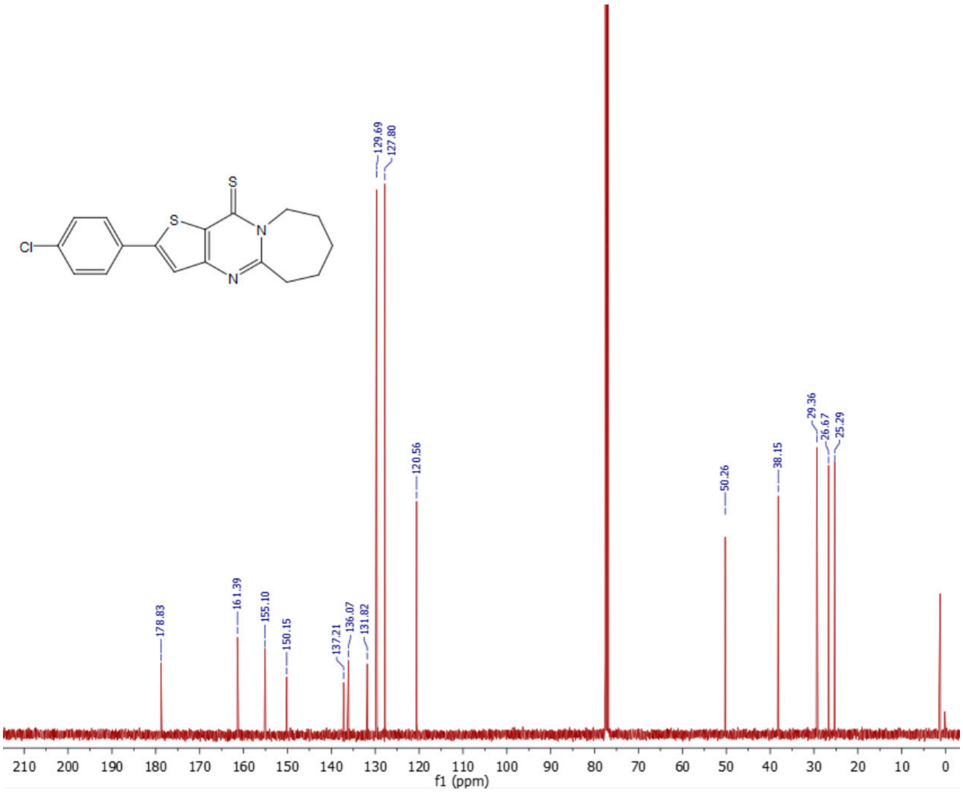

Figure S60: ^{13}C NMR spectrum of compound **6o**
